# Evolutionary diversification via modular compliance for self-reconfigurable continuum robots

**DOI:** 10.1126/sciadv.aeg9191

**Published:** 2026-08-19

**Authors:** Yilin Cai, Zhefeng Huang, Yifan Wang, Haokai Xu, Yue Chen

**Affiliations:** ^1^ Woodruff School of Mechanical Engineering, Georgia Institute of Technology, Atlanta, GA 30332, USA.; ^2^ Institute for Robotics and Intelligent Machines (IRIM), Georgia Institute of Technology, Atlanta, GA 30332, USA.; ^3^ School of Electrical and Computer Engineering, Georgia Institute of Technology, Atlanta, GA 30332, USA.; ^4^ Department of Biomedical Engineering, Georgia Institute of Technology/Emory University, Atlanta, GA 30332, USA.

## Abstract

Modular self-reconfigurable robots promise adaptability through changes in morphology, yet most existing systems remain limited by low functional density, rigid modules, and constrained docking interfaces that restrict scalable locomotion and manipulation. In contrast, biological organisms achieve rich behavioral diversity through repeated compliant segments combined with flexible articulated body architectures to support locomotion, manipulation, and environmental interaction. Here, we present a modular self-reconfigurable continuum robot that exploits modular compliance as a unifying design principle to enable cross-species bioinspired loco-manipulation within a single platform. Each module integrates a continuum backbone for compliant bending and a pair of grippers for omnidirectional, quasi-freeform docking, achieving high functional density within a compact unit. As a result, a small number of modules can assemble into diverse morphologies capable of distinct capabilities. We further develop a morphology-conditioned gait library covering rolling, undulation, crawling, quadrupedal walking, and multisegment manipulation, organized within an evolutionary diversification tree that explicitly links biological locomotion strategies to corresponding robotic assembly patterns. To enable autonomous transitions between configurations, we introduce a unified geometric-topological representation and a self-reconfiguration planner that decomposes reconfiguration into discrete grasping and releasing actions and continuous deformation actions. Hardware experiments demonstrate online self-reconfiguration, followed by integrated loco-manipulation. Together, these results show that embedding compliance at the module level unifies locomotion, manipulation, and self-reconfiguration within a single robotic platform, suggesting a pathway toward more adaptable machines that exhibit organism-like behaviors across species.

## INTRODUCTION

Modular self-reconfigurable robots (MSRRs) have long been investigated as a paradigm for adaptive and versatile machines (*1*, *2*). By combining and rearranging multiple identical modules, these systems can change the morphology to traverse different environments, perform diverse tasks, or recover from failure (*3*). This capability has motivated applications such as search and rescue in confined spaces (*4*), assistive furniture (*5*), humanitarian missions in marine environments (*6*), and terrain exploration (*7*). Although extensive research has demonstrated various forms of locomotion (*8*) and adaptable reconfiguration processes (*9*), existing MSRRs still face persistent design challenges in module mobility, docking connectivity, scalability, and robustness. First, due to the limited spatial motion ability of each single module, most systems require large numbers of modules to form functional bodies, which increases assembly complexity, cost, and operational overhead. Second, traditional one-to-one docking mechanisms impose substantial constraints on the number of modules that can be connected (*10*, *11*), reducing the variability of feasible configuration combinations and, in turn, limiting behavioral diversity. While recent work on freeform connectors (*12*–*14*) allows one-to-many connections between modules, their weak connection strength, isotropic ball geometry, and low functional-density force coordinated behaviors to rely on complex system-level synchronization. Here, functional density refers to the number of distinct capabilities integrated within each module, including locomotion, docking, reconfiguration, compliance, and manipulation. Third, most existing MSRR systems are built from rigid modules with simple geometric shapes such as balls, cylinders, or cuboids (*15*). While modular architecture naturally lends itself to forming biomimetic morphologies, the inherent rigidity of these modules prevents them from expressing the compliance required for safe interaction, terrain adaptation, and agile, biologically inspired movement in unstructured environments. Together, these limitations reveal persistent challenges in both module and connector design: improving each module’s mobility and functional density and achieving stable, flexible connections that support scalable and adaptive reconfiguration.

**Fig. 1.**
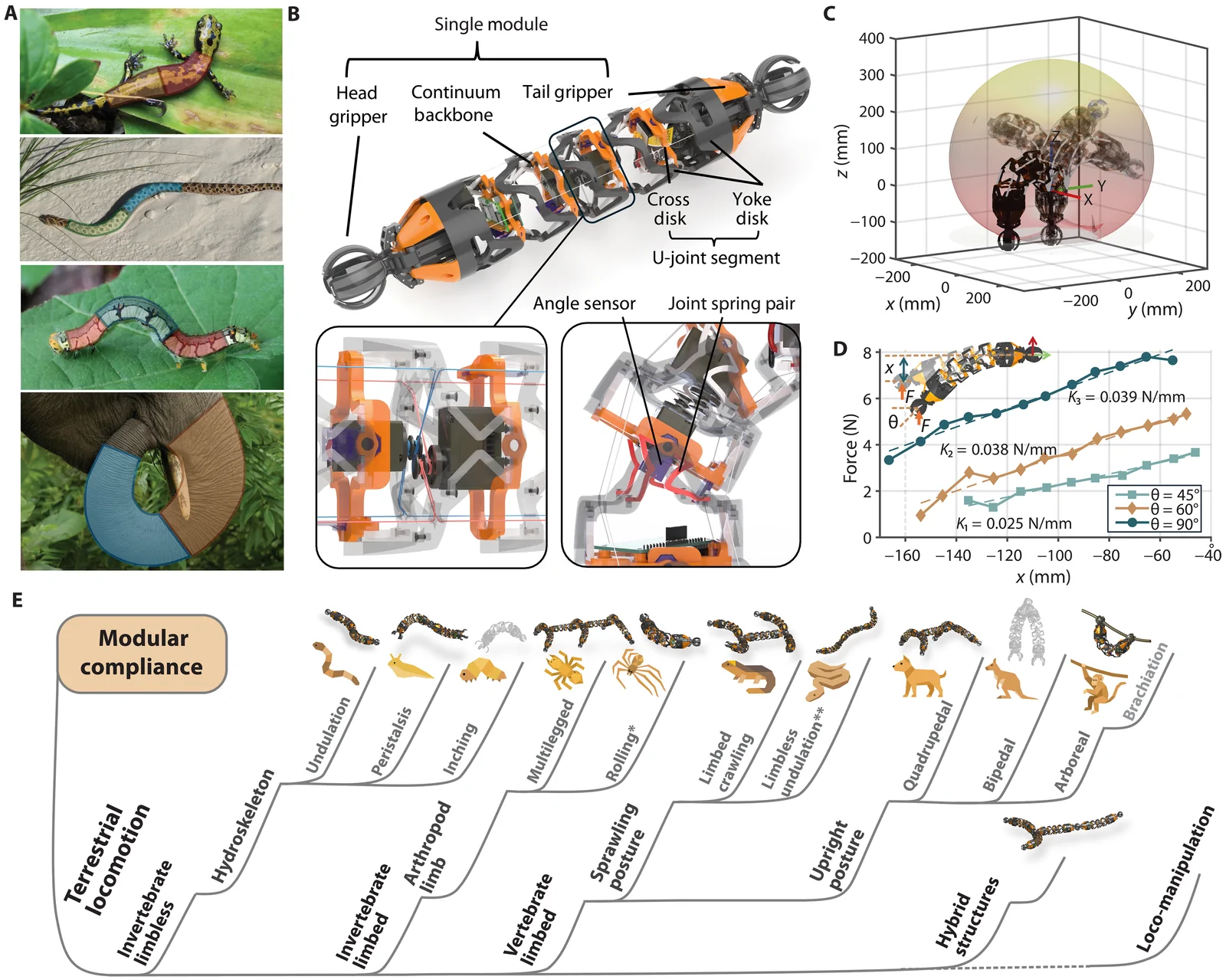
Modular compliance as a unifying principle for cross-species bioinspired locomotion and the design of the modular self-reconfigurable continuum robot system. (**A**) Examples of biological systems that exploit compliant, segment-based structures for versatile locomotion and manipulation. Photographs were adapted from public-domain or Creative Commons Zero images (*73*–*76*). (**B**) Design overview of a single modular self-reconfigurable continuum robot (MSRCR) module, where the symmetrical tendons are driven by centrally mounted motors, and the compliant spring joint pair links the neighboring joint segments. (**C** and **D**) Workspace and end-point stiffness characterization of a single module. (**E**) Conceptual evolutionary diversification tree illustrating the diversification of terrestrial locomotion strategies. Representative MSRCR morphologies are mapped onto each node to construct a library of morphology templates corresponding to distinct locomotion primitives. Gray figures indicate gaits that are not achievable by MSRCR. *Rolling locomotion is exemplified by the golden wheel spider (*Carparachne aureoflava*) (*41*). **Limbless undulation is grouped under vertebrate limbed locomotion, reflecting regressive evolution within reptiles, where snakes evolved from tetrapod ancestors through secondary limb loss while retaining axial undulatory control. Animal icons were created by the authors using basic polygon vector shapes in Adobe Illustrator.

As a biological counterpart to the motion strategies pursued in modular robotic systems, animals offer a compelling solution: Modular compliance emerges as a fundamental organizational strategy across diverse lineages (*16*), enabling agile and adaptive locomotion and manipulation (Fig. 1A). In muscular hydrostats such as the elephant trunk, repeated deformable segments capable of local bending and extension generate continuous bending (*17*). This compliance enables the arm to form stable grasping postures around irregular objects. Worms such as caterpillars rely on highly compliant body segments to anchor, pull, and conform to complex substrates, allowing them to climb, burrow, and navigate cluttered environments with minimal sensing or control. Similarly, in vertebrates such as the salamander and the snake, serially repeated vertebrae and compliant intervertebral joints create a flexible axial skeleton that distributes loads, absorbs perturbations, and generates traveling waves for efficient propulsion across diverse terrains (*18*). Across these systems, compliance does more than permitting deformation: It passively mediates environmental interaction, stabilizes contact with uneven or unpredictable surfaces, enables energy dissipation, and reduces control demands by letting the body reshape itself in response to external forces. These systems demonstrate how nature constructs complex behaviors by stacking compliant bending modules that integrate actuation, sensing, and structure into serial chains.

This compliant, segment-based modularity in nature has motivated the development of modular soft and continuum robotic systems to emulate these principles. Recent work has demonstrated a broad design space built from modular soft actuators, assembling them into manipulators (*19*–*21*) or locomotion systems (*22*–*25*). These soft modules replace traditional rigid links and joints to generate compliant deformation for safe interactions, but their fixed arrangement typically confines them to a single function or application, limiting their ability to perform dexterous loco-manipulation. Beyond fixed assemblies, reconfigurable soft robots can achieve diverse functionalities through different arrangements of modular units (*26*). For example, quadrupedal (*27*), tetrahedral (*28*), and snakelike (*29*) locomotions were enabled using the same pneumatic muscle module. However, these units are not designed for independent motion, requiring customized connectors that increase mechanical complexity and manual assembly effort. On the other hand, recent research has also advanced the development of actively reconfigurable soft robots capable of switching among a few discrete morphologies to traverse varied environments (*30*, *31*), but their nonmodular architectures inherently limit the number of achievable configurations. Although these efforts demonstrate capable motion and emphasize the importance of biomimetic design, they typically mimic a single species model, leading to robots that capture only one locomotion or manipulation strategy. In contrast, over evolutionary time, animal lineages have diverged into morphologies that support different behaviors, each tuned to their ecological niche, while organizing a shared principle of modular compliance in distinct ways. Replicating this diversity with the modular soft robots remains challenging due to design constraints inherited from rigid modular systems: (i) The lack of high–functional-density compliant modules restricts the behavioral versatility, as many soft systems still rely on geometrically simple elements (*32*–*35*) or limited bending primitives (*25*, *36*); (ii) the absence of a unified, self-contained connector design constrains module assembly, restricting the behavioral diversity. Therefore, a central question emerges: How should compliance be designed and embedded into the modular unit, and how should the compliant modules be reorganized, such that the assembled system can exploit a broad spectrum of animal-inspired behaviors?

Addressing this question requires not only appropriate hardware primitives but also a paired self-reconfiguration planning framework that enables systematic transitions across morphologies within a single platform. In conventional rigid modular systems, reconfiguration is typically handled using graph-based planning frameworks that jointly resolve correspondence matching, reachability, collision avoidance, and stability within a rapidly growing search space (*1*, *2*, *9*). This challenge is further amplified in soft systems, as compliance induces deformation-dependent constraints, and more versatile connectors may expand connection states from discrete to continuous, complicating feasible configurations and transition paths. As a result, a planning framework that explicitly accounts for compliant deformation, flexible connectivity, and discrete attachment actions remains largely unaddressed.

Inspired by evolutionary diversification in nature, we introduce the modular self-reconfigurable continuum robot (MSRCR), a hybrid-type MSRR with chain structures and quasi-freeform connectors, which embodies multimodal cross-species biomimetic behaviors. To overcome the mobility and functional-density limitations of traditional rigid modules, each MSRCR unit is built as a tendon-driven continuum backbone that provides intrinsic 2-df compliant bending up to 180°. Complementing this backbone, each module is equipped with a pair of dual-functional docking grippers that enables quasi-freeform attachment while also providing grasping ability. To answer the question of how to organize compliant modules, we developed a comprehensive morphology and gait library, spanning rolling, crawling, sprawling, snakelike undulation, and brachiation. This library aligns with the locomotion modes represented in our evolutionary diversification tree, linking each morphology to a corresponding animal template. To enable self-reconfiguration between different morphologies, we introduce a unified representation that captures both geometric and topological abstractions of MSRCR configurations, together with a self-reconfiguration planner that exploits this structure to generate smooth and physically consistent transitions between morphologies. Last, we demonstrate the capability of the MSRCR to achieve active self-reconfiguration and versatile loco-manipulation through modular compliance and cross-species biomimetic design. An overview of the robot system is presented in movie S1.

## RESULTS

We first clarify the terminology used throughout the paper. A configuration denotes the complete state of an MSRCR, including both its connectivity and its full geometric realization, i.e., the grasping angles at all docking interfaces, the bending state of each module, and the global pose of the system in Cartesian space. The topology of a configuration encodes which pairs of modules are connected. The morphology extends the topology by assigning specific connection angles between modules while not specifying the bending of individual modules. The shape further extends the morphology by incorporating instantaneous bending states of all the modules while not specifying the absolute position and orientation of the whole system in the world. Rigorous mathematical definitions are given later.

### Design of the MSRCR system as a building block for cross-species bioinspiration

We demonstrate a module-level design that abstracts key traits of biological compliant bodies into a unified robotic building block. Each module integrates three functional components: a tendon driven continuum backbone, a gripper pair with dual function of both grasping and docking, and onboard sensing and electronics (Fig. 1B). The backbone consists of five serial universal joint segments, each composed of a yoke disk and a cross disk. Two orthogonal tendon pairs routed through all segments generate planar bending about two independent axes, providing 2-df bidirectional bending. Adjacent U-joint segments are mechanically coupled by compliant V-shaped spring pairs positioned between their yoke and cross disks (Fig. 1B). During bending, one spring arm compresses while the other elongates, producing a restoring torque that passively stabilizes the backbone and returns it toward its neutral posture.

Conventional continuum robots typically require large proximal actuation modules where all motors, electronics, and power distribution remain fixed at the base, limiting untethered mobility and deployment (*37*). To overcome these limitations, we proposed a center-coupled tendon actuation scheme in which the two motors are mounted on a central yoke disk within each module to drive the compliant backbone. Each motor drives one pair of antagonistic tendons. Pulling one tendon while simultaneously lengthening its counterpart generates bending in one direction. The module’s passive stiffness is determined by the joint spring pair and can be tuned by changing its geometry. During actuation, the motor holds the tendon lengths, which helps resist deformation around the bent shape. The tendon on each side is divided into symmetric paths that terminate at opposite ends of the backbone, allowing one motor to pull both halves of the module simultaneously (Fig. 1B). Tendon slack was reduced by initial pretension during assembly, and the tendon terminations at the end disk were designed to allow manual pretension adjustment after repeated operation. Angle sensors at each joint measure the local bending angles and reconstruct the full bending profile. The hollow universal-joint disks house the motors, wiring, and electronics, enabling a compact, self-contained module without external actuators or tethered power (figs. S1 and S2). This untethered design enables a free mobile continuum module, which is essential for field deployment and on-site reconfiguration, and represents a clear advance over fixed-base continuum robots (*38*). It also differs from many tethered soft robots, such as shape memory alloy (SMA)–driven systems with slow thermal cycles (*39*) or pneumatic platforms that rely on bulky external pressure regulation hardware (*40*).

The 2-df bending of a single MSRCR module generates a smooth hemispherical workspace under a constant curvature assumption–based kinematics formulation, reaching about 180° (Fig. 1C). Rigid modular robots produce segmented and directionally biased reach through stacked joints, while the MSRCR’s compliant universal joint chain generates curvature directly, giving each unit a broader and more isotropic workspace. We also measured the effective bending stiffness of each module under tip loading (Fig. 1D). In the test setup, the module is clamped at one end to represent its attachment to the upstream body segment, while the backbone bends downward under gravity and is actuated into the different bending angles (θ = 45°, 60°, and 90°) during ground contact. We applied an upward force at the free end, representing the ground reaction force during thrust, and recorded the corresponding displacement Δx. The resulting force-displacement curves yielded effective stiffness values of $K_{1}=0.025$ N mm^−1^, $K_{2}=0.038$ N mm^−1^, and $K_{3}=0.039$ N mm^−1^. At θ = 90°, the module sustained ∼7 to 8 N while remaining in the downward-bending regime, indicating that it can transmit sufficient thrust without being pushed back to the neutral posture or inverted to the opposite direction. Smaller bending angles exhibited lower peak force, reflecting the trade-off between compliance and load- bearing. The results show that the module achieves an intermediate stiffness regime that is stiff enough to transmit reliable ground reaction forces for locomotion while remaining compliant enough to absorb perturbation and conform to terrain.

To contextualize the role of modular compliance in shaping terrestrial locomotion, we then organized representative biological and robotic morphologies into an evolutionary diversification tree of locomotion (Fig. 1E). Rather than following a rigorous phylogenetic topology, this tree traces the progressive elaboration of locomotor strategies, progressing from soft-bodied deformation-based motion to articulated-limb–driven gaits and ultimately to hybrid structures capable of both locomotion and manipulation.

At the tree’s base lies the hydroskeletal lineage, where invertebrate limbless organisms such as worms, nematodes, and caterpillars use distributed muscular hydrostats to produce body-deformation–based gaits, including undulation, peristalsis, and inching. These forms embody the simplest expression of modular compliance: Movement emerges entirely from the sequential deformation of continuous, soft segments. Arthropods build upon this principle by modularizing compliance into repeated jointed limbs, enabling coordinated multilegged gaits and even rolling (*41*) with greater speed and terrain adaptability. Vertebrate evolution introduces further diversification (*42*). Sprawling limbed animals combine limb-driven crawling with trunk flexibility, while some lineages secondarily lose their limbs and return to undulatory propulsion (*43*, *44*). Upright postures in mammals and archosaurs decouple limb and trunk mechanics, supporting efficient quadrupedal and bipedal locomotion, as well as specialized strategies such as brachiation (*45*). Beyond natural evolutionary pathways, hybrid structures emerge in robotics by recombining biological templates. Robotic systems can mix limb and body compliance, introduce reconfigurable morphologies, or repurpose modules for both support and manipulation. This leads to loco-manipulation behaviors where segments that produce movement also generate grasping or interaction forces, extending the functionality beyond what is typically possible in single-species biological systems. Overall, this framework reflects a continuous transformation from soft-bodied, segmental organisms to articulated, functionally diverse systems. Although the proposed robotic modules do not actively change length or volume to fully mimic invertebrate behaviors, the framework guides how we arrange robotic modules into a library of morphology templates that map directly to distinct locomotion primitives, providing a systematic link between biological diversity and reconfigurable robotic behaviors.

### Design and characterization of the quasi-freeform omnidirectional docking gripper

**Fig. 2.**
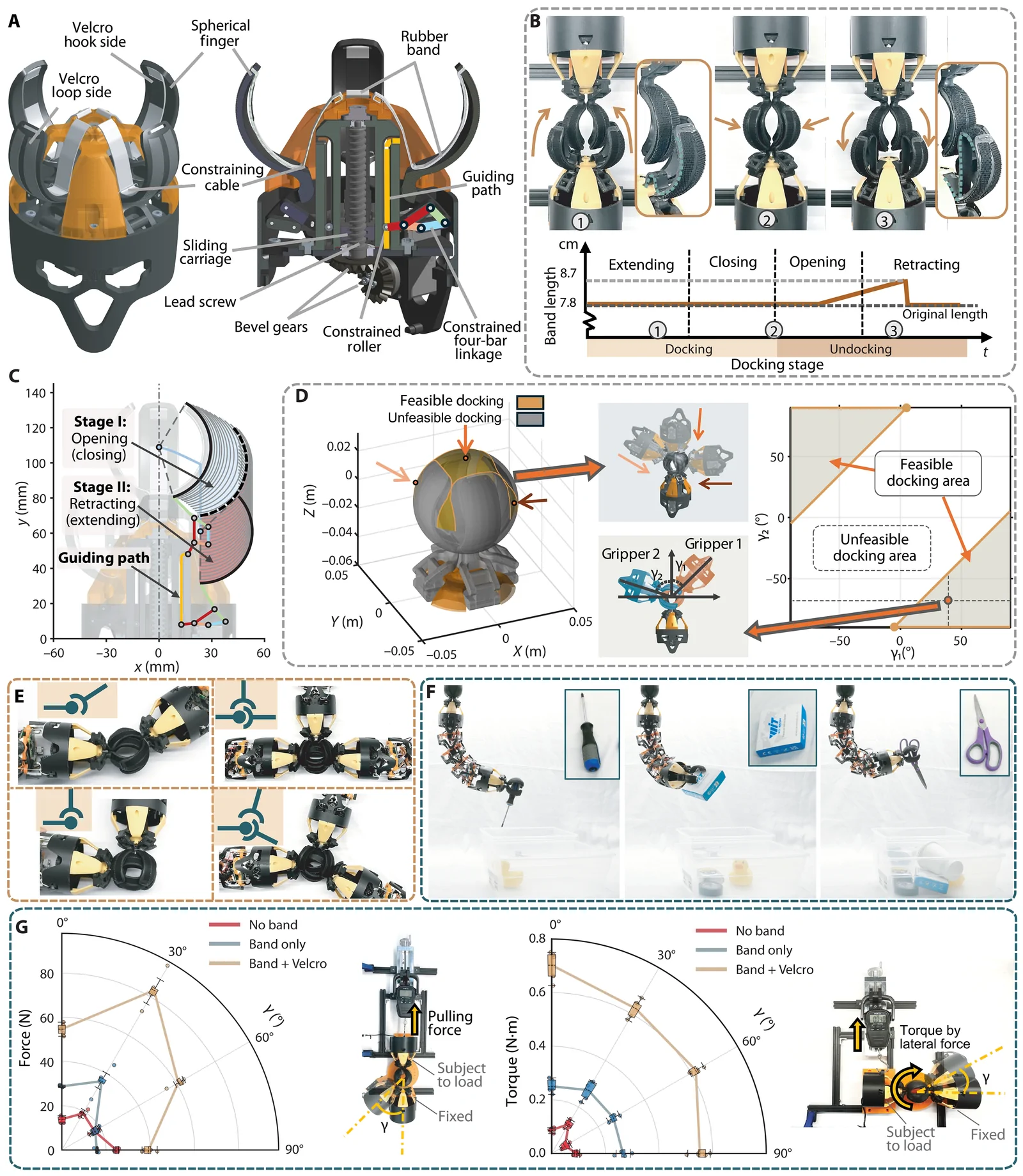
Design and characterization of the omnidirectional docking gripper. (**A**) Design of the gripper structure. (**B**) Two-phase single-input mechanism enabling docking and undocking. The rubber band will be elongated during the undocking process. (**C**) Shell motion sequence of the two phases during the docking and undocking. (**D**) Feasible docking ranges for one-to-one and multigripper engagement. (**E**) Grasping-as-docking demonstration with multidirectional approaching and attachment. (**F**) Docking gripper manipulating daily objects (screw driver, box, and scissors) with various shapes. (**G**) Measured force and torque for docking stability analysis (data sample number = 5).

To enable a freeform docking mechanism for omnidirectional connection between modules, we then extend the grasping-as-docking (*46*) concept that unifies manipulation and reconfiguration under a single mechanical principle. Each module is equipped with a spherical docking gripper capable of both grasping another module and being grasped by another, eliminating the need for distinct male-female connectors (Fig. 2A). Closing the gripper transforms its open structure into a compact spherical surface. Another gripper can then encompass and grasp this spherical connector from a continuous feasible set of approach angles, and once closed, it likewise presents a spherical surface for subsequent attachments. This process can be repeated to allow up to four grippers to dock at the same node, subject to the collision-free angular constraints analyzed below. Once engaged, the relative angle between grippers is fixed. Changing the docking angle requires disengaging and reapproaching from a different angle. This mechanism thus realizes a quasi-freeform, omnidirectional docking architecture, which is defined as allowing arbitrary connection sequences but with fixed relative orientation upon engagement.

The docking gripper is divided into four rigid shells that together form a segmented spherical enclosure around a central hub. To enhance contact compliance and attachment strength, we equipped each shell with a rubber-band lining on its inner surface and a Velcro-based adhesive interface. The hook side of the Velcro is embedded on the rubber band, while the loop side is attached to the outer surface of each shell. When two grippers close around each other, these complementary surfaces interlock, forming a soft yet strong mechanical bond that converts normal pressure into tangential holding strength. The rubber band distributes the contact load evenly and introduces elastic pretension that helps the interface self-reengage after small disturbances.

The gripper motion is governed by a two-phase single-input mechanism, geometrically programmed into a constraint-guided four-bar linkage. The four-bar linkage includes a guided roller that slides along a predefined curved path, coordinating the motion of all shells to maintain symmetry. The actuator’s translation is geometrically divided into two motion phases (Fig. 2, B and C). In phase I (opening/closing), the four-bar linkage rotates, causing the shells to pivot outward or inward, while the guided roller moves approximately parallel to the base. This coordinated motion produces the enveloping grasp necessary for docking another gripper. In phase II (retracting/extending), once the linkage reaches its transition point, all four-bar linkages begin translating collectively upward or downward along the guiding path without further shell rotation. This motion pulls the rubber bands downward, producing a controlled peeling motion that separates the Velcro hook-loop interface during undocking. During this process, the band gradually elongates as the shells retract, providing increased force for peeling.

The feasible docking range is governed by the collision-free closure geometry of the physical grippers (Fig. 2D). For the spatial one-to-one docking case, the gripper supports docking over a continuous range of admissible orientations, with the feasible region partitioned by the shell geometry. A larger feasible region appears near the top of the gripper, where docking directions are less constrained by the shells. For the planar one-to-one docking that occurs in the cross-sectional plane passing through the shell gaps, the feasible docking range is from −95° to +95°, determined by collision-free shell closure (fig. S3). For planar multidocking, the same collision-based analysis yields a bounded feasible region in the $\left(\gamma_{1},\gamma_{2}\right)$ orientation space, where adjacent grippers must be separated by more than 85° to avoid shell interference and allow full closure. These limits are set by the current gripper geometry and can be adjusted in future designs by modifying the shell profile, gap width, or opening clearance. Notably, the docking process tolerates moderate misalignment because the open-shell geometry allows the grasped gripper to be passively guided into alignment when the shells close. Demonstrations of two- and three-gripper docking are shown in Fig. 2E and movie S1.

Beyond grasping for docking, general manipulation ability was assessed using a set of everyday items spanning soft objects, rigid tools, slender shapes, irregular geometries, and small consumer items (representative objects shown in Fig. 2F; full demonstration in fig. S5 and movie S2). Under a single approach, close, lift, and place sequence, the gripper consistently formed stable grasps and transported each item without slip, demonstrating reliable handling across wide shape and material variations. The pick-and-place kinematic control was achieved by teleoperation as detailed in Supplementary Text.

The grasping and docking stability were quantified by measuring the resisting force and torque required to initiate slippage between two docked grippers (Fig. 2G). One gripper was rigidly fixed, while the other was subjected to a gradually increasing pulling or twisting load at various closure angles γ. The peak value before slip was recorded as the holding capacity. Three surface configurations were compared: no band, band only, and band + Velcro. The results reveal an enhancement of both holding force and resisting torque with each modification. Without the band, contact occurred mainly at discrete shell edges, yielding low friction and early slip. Adding the compliant rubber band improved contact conformity and pressure distribution, approximately doubling the holding strength. Incorporating the Velcro layer further boosted stability, achieving up to 60 N of resisting force and 0.6 N·m of torque at γ = 90°. The rubber band and Velcro combination effectively converts normal preload into shear-resistant adhesion through microhook interlocking, while the elastic backing enables progressive load sharing and smooth detachment rather than abrupt failure. Additional tests further show that the Velcro-enhanced docking interface remains functional under contamination of water, sand, grass, and leaf debris, retaining more than 30 N of pulling force even when leaf debris partially blocks shell contact (fig. S13).

### Morphology and gait library for locomotion

**Fig. 3.**
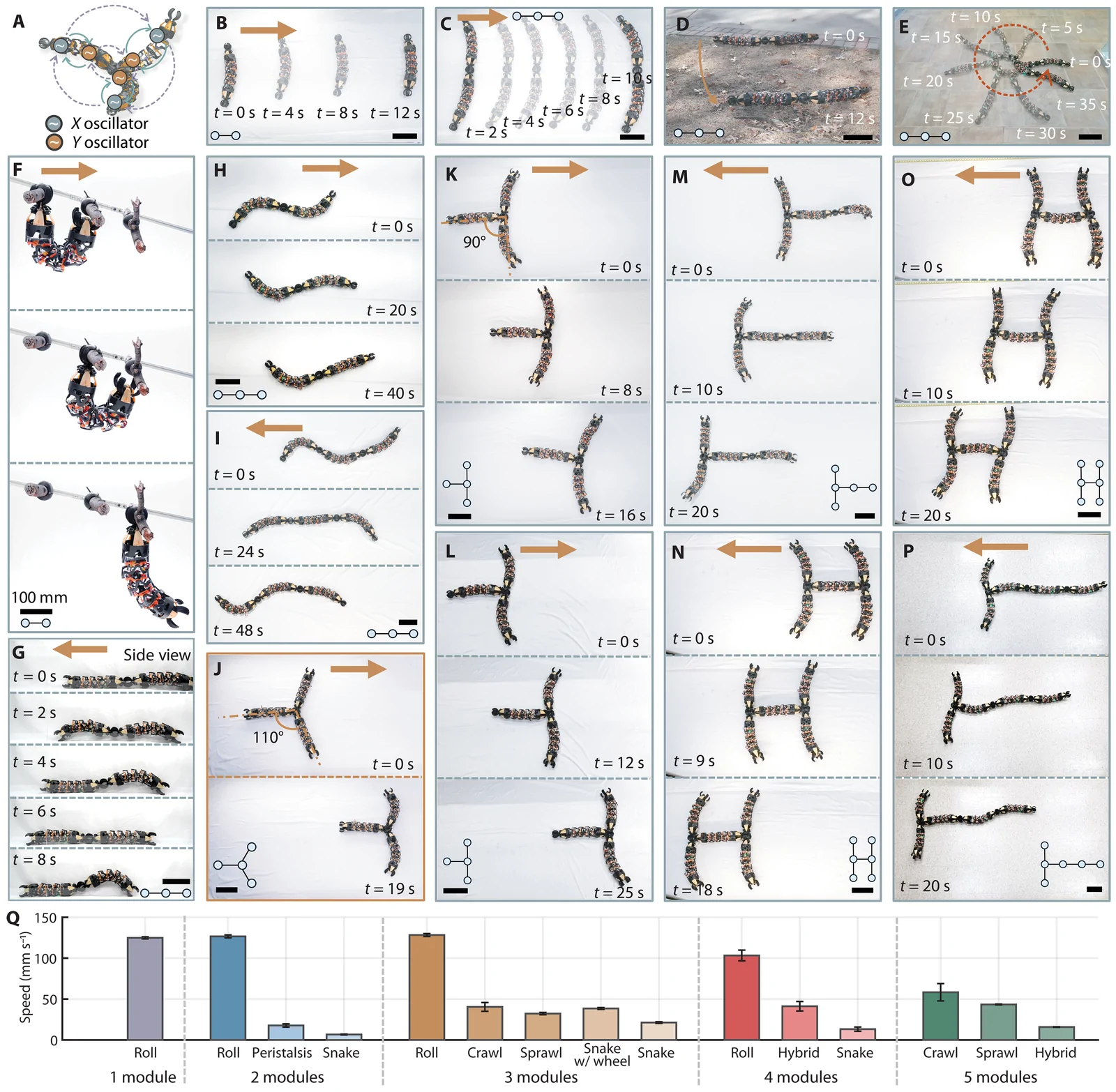
Morphology and gait library of the MSRCR. (**A**) Unified CPG coordination for rhythmic module actuation. (**B** to **D**) Single and two-module rolling, including outdoor locomotion. (**E**) Two-module rolling-based pivot turning. (**F**) Single module brachiation. (**G**) Two-module peristalsis gait. (**H** and **I**) Two- and three-module snakelike undulation. (**J** and **K**) Three-module crawling. They share the same connection graph (topology) but differ in geometric arrangement (morphology). (**L**) Three-module sprawling. (**M**) Four-module crawling hybrid with tail peristalsis. (**N** and **O**) Five-module crawling and sprawling gaits. (**P**) Five-module sprawling hybrid with tail undulation. (**Q**) Average locomotion speed across representative morphologies and gaits. Error bars indicate the SD across three repeated trials (N=3). Scale bars, 100 mm (F) and 200 mm (all other panels unless otherwise noted).

Building on the module-level actuation and docking primitives, we next develop a morphology-conditioned gait library that links how modules are assembled to how they move. The library is organized by a set of reusable bioinspired morphology templates and intermodule coordination templates. This structure aligns with the evolutionary diversification tree in Fig. 1E: As the robot grows from a single segment to longer bodies and then to branched forms, it transitions from rolling-dominated transport to wave-based locomotion and multicontact, limb-like gaits. Inspired by biological central pattern generators (CPGs) (*47*–*49*), which produce coordinated rhythmic motion underlying locomotion, we adopt a coupled phase oscillator formulation. Each continuum module is governed by a coupled phase oscillator model to generate rhythmic motion, where coupling specifies how oscillator phases influence each other (Fig. 3A and fig. S6). Within a module, the *X* and *Y* oscillators are bidirectionally coupled to lock their relative phase and enforce a stable local bending pattern. Between modules, coupling is directed and arranged as a closed cycle around the connected modules. This ring structure enforces cyclic phase consistency and prevents open-end phase drift. By changing the intermodule coupling topology and phase biases, the same CPG structure generates diverse gaits across different module counts and morphologies.

Figure 3, fig. S7, and movies S3 to S7 summarize representative gaits and their corresponding speeds. Here, we only focus on the planar MSRCRs that remain entirely on the ground, for the purpose of simplifying stability and contact constraints during self-reconfiguration (see Discussion). In this work, gaits are categorized primarily by limb contact sequencing and interlimb coordination. As shown in fig. S7A, motions with synchronous bilateral limb pushes are referred to as crawling. In contrast, sprawling gaits use alternating limb support with staggered propulsion, resulting in an alternating-support contact sequence while the body remains low and ground conforming. Thus, crawling and sprawling share a similar posture but differ in coordination: Crawling generates propulsion through simultaneous bilateral strokes, whereas sprawling relies on alternating unilateral push phases. This shift in limb coordination resembles the transition from crawl-like to sprawl-like locomotion observed across biological systems (*50*).

The single-module morphology supports rolling and brachiation gaits. The rolling gait produces continuous forward rolling by cycling tendon-driven bending along the backbone, resulting in a speed of ∼125 mm·s^−1^. A brachiation motion is teleoperated to demonstrate the ability of a single module to swing between branches, highlighting its capability for manipulation-as-locomotion. As the system scales to two serially connected modules, coordinated deformation across segments enables propulsion modes that rely on distributed contact rather than pure rolling. While rolling speed remains largely unchanged, indicating that added compliance does not hinder rolling efficiency, undulatory and peristaltic gaits emerge with lower speeds but increased environmental compatibility. The snake gait combines constant upper bending and periodic lateral undulation, pushing the body forward at 6.7 mm·s^−1^. Peristalsis gait allows the robot to move in a narrowed vertical channel at 17.4 mm·s^−1^. In addition to indoor forward motion, outdoor motion and turning motion are also demonstrated in Fig. 3E and fig. S7. These results highlight a trade-off between speed and environmental adaptability.

Three-module morphologies expand both serial and branched connections, supporting rolling, snake, crawling, and sprawling gaits. The emergence of crawling (40.4 mm·s^−1^) and sprawling (32.2 mm·s^−1^) gait coincides with a transition from body-dominated propulsion to contact-sequenced limb support. The snake gait also combines constant upper bending and periodic lateral undulation but achieves a higher speed (21.26 mm·s^−1^) than in the two-module case, reflecting increased lateral excursion and improved force transmission along a longer body. Even without passive wheels such as in (*22*, *51*), the imposed upper bending reshapes the body-ground contact distribution, aligning temporal friction patterns with the phase of lateral undulation to generate directional propulsion (*52*). Passive wheels can be readily attached to the module as an accessory, introducing nonhomogeneous friction that enables faster locomotion (38.5 mm·s^−1^) (fig. S7, C and D). Four-module morphologies introduce hybrid behaviors, combining two-arm crawling with tail-driven peristalsis to enable multicontact propulsion at 41.2 mm s^−1^. Five-module morphologies further enrich the gait repertoire, introducing four-limb crawling, sprawling, and hybrid sprawl-snake gait. The distributed ground contact and coordinated deformation provide more robust locomotion.

Although rolling remains the most speed- and energy-efficient locomotion mode in our system, such mechanisms are rare in biological organisms. As morphology evolves from simple serial chains to more complex branched configurations, the locomotion repertoire expands beyond rolling to include coordination-rich gaits that rely on distributed contact and phased force generation. This transition mirrors biological locomotion, where increasing body complexity favors gaits that trade peak efficiency for greater stability, maneuverability, and environmental adaptability.

#### Morphology variation with the same topology

The gait library further reveals that effective locomotion can be specified at the level of topology rather than a single geometric realization. In simulation, a three-module crawler with the same connection but varying arm-to-tail connection angles exhibits markedly different locomotion under identical CPG settings (fig. S8). Compared with the 90° arm-to-tail angle configuration, forward displacement decreases by 23.4% at 100°, 57.1% at 110°, and 77.9% at 120° despite identical CPG coordination. This performance gap can be largely eliminated by modifying only a static initial bias that shifts the neutral bending configuration of each module. By centering the oscillatory swing plane around a direction approximately perpendicular to the body axis, forward displacement across morphologies converges, reducing the error relative to the 90° case to within 1.3% for 100°, 0.5% for 110°, and 4.3% for 120% in simulation (fig. S8). Experimental results in Fig. 3 (J and K) validate this trend, showing that configurations sharing the same topology execute stable crawling with comparable performance despite substantial geometric differences. The results indicate that topology-conditioned coordination patterns can generalize across a family of morphologies through minimal, interpretable bias adjustments rather than controller retuning.

### Representation of MSRCR configurations

To enable reconfiguration between morphologies in the MSRCR gait library, we first introduce a unified representation for configurations that jointly encodes topological connectivity and geometric shape. Unlike rigid modular robots with discrete connections and fixed geometry, MSRCR configurations arise from continuous connection angles and ordered grasping relations enabled by omnidirectional grippers. As a result, identical connectivity can correspond to distinct geometric shapes, making topology-only representations used for rigid modular systems (*15*) insufficient for reasoning self-reconfiguration here.

#### A unified geometric-topological representation of MSRCR configurations

**Fig. 4.**
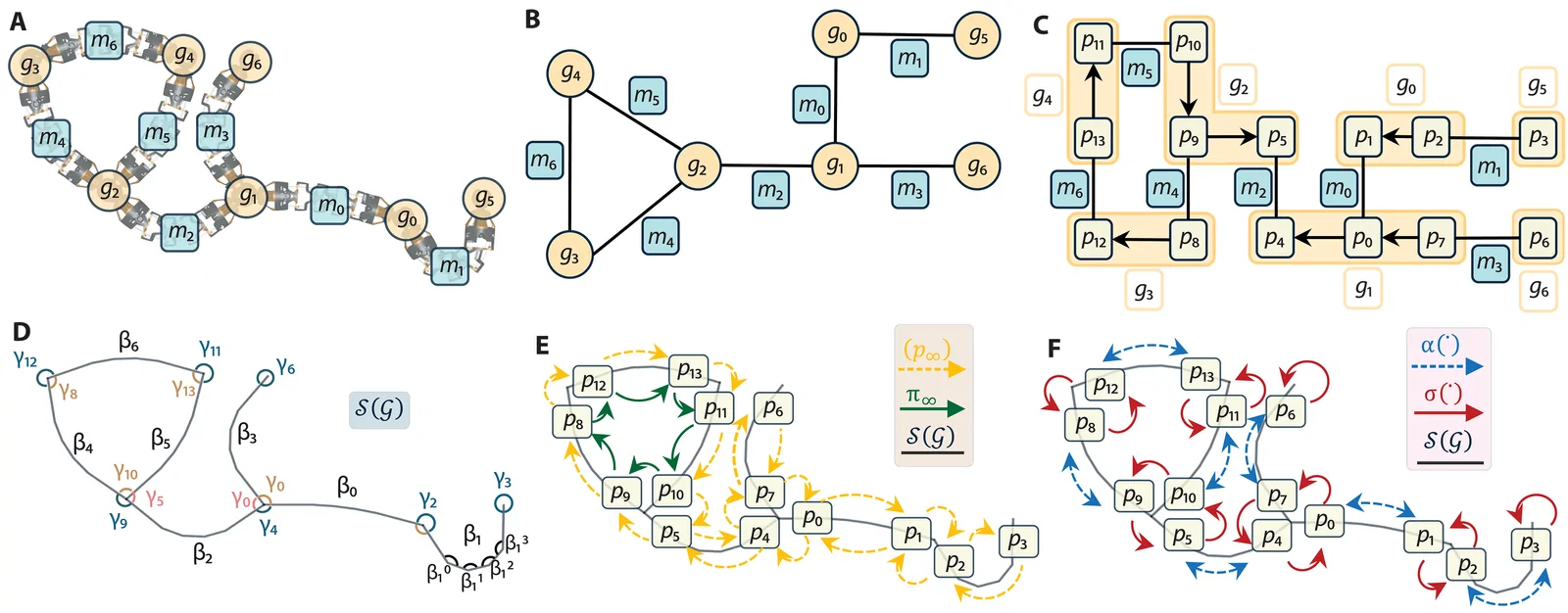
MSRCR configuration representations. (**A**) Example planar configuration with seven modules and seven grips; each grip g is either a chain of grippers connected through pairwise grasping relations ($g_{0},g_{1},g_{2},g_{3},\;\text{and}\;g_{4}$) or a single gripper that does not participate in any grasp ($g_{5}$ and $g_{6}$). (**B**) Module-level topology represented by the undirected graph G. (**C**) Gripper-level connectivity structure represented by the mixed graph $G_{M}$ with undirected edges for module links and directed edges for grasping relations. (**D** to **F**) The shape S of a configuration is given by a planar embedding of G, whose edges are drawn as equiangular polylines. The bending angle β of a module is defined as the angle between its incident U-joint segments, and the grasping angle γ is the angle between neighboring grippers within a grip. Interpreting each gripper $p_{i}$ as a dart (half-edge) yields a rotation system R that encodes the cyclic ordering σ and head-tail gripper pairing α and an outer-face boundary permutation $\pi_{\infty }$ obtained by traversing the outer boundary of the embedding clockwise.

A planar MSRCR configuration is determined by the gripper docking relations, the associated relative docking angles, and the individual module bending angles on an orientable, flat, and effectively unbounded ground plane, ${\mathbb{R}}^{2}$. To capture the resulting combination of discrete connectivity and continuous geometry, we present a unified geometric-topological representation of MSRCR configurations. An example of a seven-module configuration is shown in Fig. 4. A complete configuration C is represented as

C={M,PS,G,B,A,π∞} (1)

First, the set $\left\{M,P_{S},G\right\}$ encodes the topology G and the connectivity structure $G_{M}$ of a configuration. When the grippers dock, they form a grip with nested grasping order. For an MSRCR with $N_{m}$ modules and $N_{g}$ grips (Fig. 4A), G and $G_{M}$ are encoded by sets of modules M, grippers P, grips G, and a partial order $\leq_{S}$ denoting grasping relations

$$\left\{\begin{array}{cc} M={\left\{m_{i}\right\}}_{i=0}^{N_{m}-1}, & m_{i}\;\text{denotes the module}\;i\\ P={\left\{p_{i}\right\}}_{i=0}^{2N_{m}-1}, & p_{2i}\;\text{or}\;p_{2i+1}\;\text{denotes the head or tail}\;\;\text{gripper of}\;m_{i}\\ P_{S}=\left(P,\leq_{S}\right), & p_{i}\leq_{S}\;p_{j}\Leftrightarrow p_{i}\;\text{is enclosed within the grasp of}\;p_{j}\\ G={\left\{g_{i}\right\}}_{i=0}^{N_{g}-1}, & \forall i,g_{i}\;\text{is}\;a\;\text{ground}\;\text{set}\;\text{of}\;a\;\text{maximal chains in}\;P_{S} \end{array}\right.$$ (2)

From this description, G and $G_{M}$ are then constructed as two planar graphs. The topology G is an undirected graph where grips are vertices and modules are edges (Fig. 4B). The connectivity structure $G_{M}$ is a mixed graph where grippers are vertices, modules are undirected edges, and grasping relations are directed edges (Fig. 4C).

Second, the set $\left\{B,A,\pi_{\infty }\right\}$ parameterizes the shape embedding S of a configuration. The shape embedding S is defined as a planar graph embedding $S:\left(G|B,A,\pi_{\infty }\right)\to {\mathbb{R}}^{2}$, and the shape of a configuration is thus S(G) (Fig. 4D). This embedding specifies how G is realized as a non–self-intersecting geometric structure on a plane, with each edge of G mapped as an equiangular polyline consistent with robot bending. Accordingly, this embedding S is parameterized by the set of bending angles B, the set of grasping angles Γ, the rotation system R, and the permutation $\pi_{\infty }$ of grippers on outer face boundary (Fig. 4, E and F)

$$\left\{\begin{array}{cc} B={\left[\beta_{0},\beta_{1},\ldots ,\beta_{N_{m}-1}\right]}^{T}, & \beta_{i}\;\text{is the bending angle of}\;m_{i}\;\text{from head to tail}\\ R=\left(P,\sigma ,\alpha \right), & R\;\text{is the combinatorial}\;\text{map}\;\text{of}\;S\left(G\right)\\ A=\left(R,\Gamma \right), & \Gamma ={\left[\gamma_{0},\gamma_{1},\ldots ,\gamma_{2N_{g}-1}\right]}^{T}\text{is grasping angle vector}\\ \pi_{\infty }:P_{\infty }\to P_{\infty }, & \pi_{\infty }\left(p_{i}\right)\;\text{is the successor of}\;p_{i}\;\text{in}\;\left(p_{\infty }\right);p_{i}\in P_{\infty } \end{array}\right.$$ (3)

Here, ∞ is the point at infinity. For a shape S(G), clockwise traversing the boundary of its outer face produces a sequence of encountered grippers, where repeated visits to the same gripper is allowed. This sequence is denoted by $\left(p_{\infty }\right)$ (yellow dashed arrows in Fig. 4E). Removing all duplicate grippers in $\left(p_{\infty }\right)$ yields a subset $P_{\infty }$, and the permutation $\pi_{\infty }$ on this subset maps a gripper to its successor in the sequence $\left(p_{\infty }\right)$. Counterclockwise traversing grippers participating in the same grips yields the cyclic order permutation σ (red solid arrows in Fig. 4F), and mapping each gripper $p_{i}$ to its complementary gripper on the same module yields the involution α (blue dashed arrows in Fig. 4F). Then, by treating all grippers as darts, a combinatorial map R=(P,σ,α) representing the rotation system of a configuration is formally defined (*53*). Each grasping angle $\gamma_{i}$ is then defined as the signed angle from $p_{i}$ to its counterclockwise successor $\sigma \left(p_{i}\right)$. By combining R with Γ, we further define the angle system of a configuration as A=(R,Γ). Last, with this representation in place, for a configuration C, its topology G and shape S(G) are fully defined.

#### Shape and kinematic equivalence classes of MSRCR configurations

We now formalize two equivalence relations, shape equivalence ${∼}_{s}$ and kinematic equivalence ${∼}_{k}$, which serve as core planning primitives across motion- and task-level reasoning. First, two configurations $C_{0}$ and $C_{1}$ are shape equivalent, denoted $C_{0}{∼}_{s}C_{1}$, if there exists a planar rigid-body transformation T∈SE(2) such that their graph embeddings will coincide, i.e., $T\left(S_{0}\left(G_{0}\right)\right)=S_{1}\left(G_{1}\right)$. Shape equivalence thus serves as the goal-reaching criterion for motion-level planning. Second, two configurations $C_{0}$ and $C_{1}$ are kinematically equivalent, denoted $C_{0}{∼}_{k}C_{1}$, if there exists a deformation connects $C_{0}$ to a configuration $C_{0}^{\prime }$ that is shape equivalent to $C_{1}$. For a configuration $C_{0}$ with bending angles $B_{0}$, a deformation is defined by a bending-angle trajectory $\tau :\left[0,1\right]\to {\mathbb{R}}^{N_{m}}$ with $\tau \left(0\right)=B_{0}$, producing a time-varying configuration C(t). The deformation is valid if it remains self-collision free and preserves the angle system A(t)=A(0) for all t∈[0,1]. In another word, deformation is through continuous bending alone and preserving both G and $G_{S}$ without grasping or release. All configurations that are kinematically equivalent to $C_{1}$ form the equivalence class ${\left[C_{1}\right]}_{k}$. This equivalence class serves as the goal set for task-level planning.

To evaluate kinematic equivalence without explicit motion planning, we relax the requirement of a physically realizable bending-angle trajectory τ to the existence of an angle-system–preserving isotopy. This relaxation yields a necessary condition for kinematic equivalence. We prove that two configurations admit such an isotopy if and only if they share the same angle system A and the same outer face boundary permutation $\pi_{\infty }$, i.e., the same morphology, under an appropriate correspondence between their gripper sets (see Supplementary Text for the proof). Although only necessary, this condition captures rich structural information and, in particular, establishes the angle system A as the central invariant for kinematic equivalence evaluation. We then use a graph neural network (GNN) to encode A for evaluating the equivalence.

### Self-reconfiguration between MSRCR configurations

To jointly handle continuous deformation, continuous connection angles, and discrete grasping or releasing actions, we formulate MSRCR self-reconfiguration as a task and motion planning problem. Building on this formulation, we develop and benchmark the task planning component on a procedurally generated configuration dataset. A key assumption in our formulation is that reconfiguration occurs only through connected structures, that is, individual modules are not allowed to move freely after detachment. This choice is motivated by the fact that, unlike many rigid modular robots with independent locomotion capability, a single soft continuum module in our system mainly supports local bending and cannot arbitrarily reposition itself in the plane. Maintaining connectivity therefore allows multiple modules to coordinate their deformations to realize reconfiguration while eliminating the need for global external pose tracking for all the modules. Another key constraint is that, once a grasp is established, the connection angle remains fixed, as determined by the docking gripper design.

#### *Problem formulation*, *state-space structure, and task-motion decomposition*

We define MSRCR self-reconfiguration planning as finding a sequence of collision-free actions (deformations, grasps, and releases), which transforms an initial configuration $C_{0}$ into $C_{0}^{\prime }$ such that $C_{0}^{\prime }$ is shape equivalent to a target $C_{1}$ under some correspondence $f_{c}:P_{0}\to P_{1}$. In other words, the objective of planning is not to exactly reproduce $C_{1}$ but to reach any configuration that is shape equivalent to $C_{1}$ under an appropriate correspondence mapping between grippers. Formal definition of correspondence is provided in Materials and Methods.

**Fig. 5.**
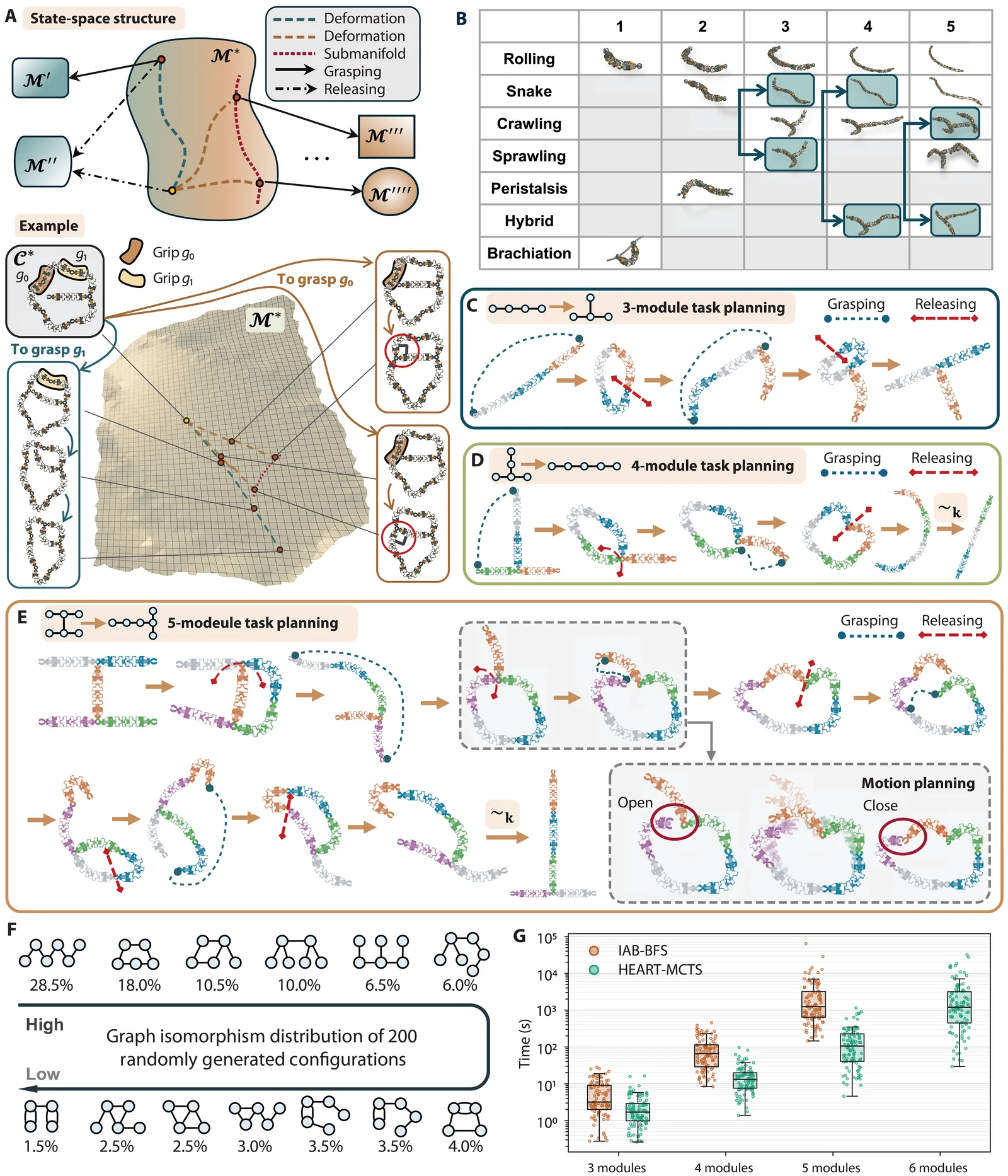
MSRCR self-reconfiguration planning. (**A**) State-space structure of MSRCRs: a locally infinite graph with constraint manifolds as vertices and grasping and releasing actions as edges. Deformations are continuous trajectories within a manifold that bring a gripper and a grip into overlap to enable grasping. Varying the grasping angle induces a submanifold connected to infinitely many neighboring vertices. Example six-module deformation for grasp preparation: three continuous trajectories on the constraint manifold constructed by sampling, visualized via projecting into ${\mathbb{R}}^{3}$. The longest trajectory targets grip $g_{0}$; the other two target grip $g_{1}$ with different grasping angles and lie on the same submanifold. (**B**) Summary of morphology and gait library for self-reconfiguration. Arrows indicate the example in-library reconfiguration sequences shown in (C) to (E). (**C** to **E**) Example task and motion planning output for self-reconfiguration among three-, four-, and five-module configurations in the library. (**F**) Sampling distribution over isomorphism classes of configuration graph representations G for $N_{m}=5$; simpler classes are sampled more frequently due to larger feasible domains of grasping and bending angles. (**G**) Task planning benchmarks on 400 procedurally generated start-goal pairs ($N_{m}\in \left\{3,4,5,6\right\}$, 100 pairs each), showing substantially reduced planning time from iterative auxiliary-budget breadth-first search (IAB-BFS) to Monte Carlo tree search constructed on a hierarchical expansion-as-rollout tree (HEART-MCTS).

Prior approaches establish configuration correspondences via graph isomorphisms (*9*, *54*–*57*). For MSRCRs, this is insufficient: Topology alone does not determine geometry, and graph-only matching often yields poor correspondence hypotheses. Another challenge arises from the continuous range of feasible grasping angles, which renders the action space effectively continuous. To make planning tractable, we therefore first characterize the structure of the state space induced by three action primitives: deformation, grasp, and release. For a given configuration $C^{*}$, we found that its kinematically equivalent class under identity correspondence forms a deformation manifold $M^{*}\subset {\mathbb{R}}^{N_{m}}$ (see Methods and Materials for formal definition). By construction, any curve $c:\left[0,1\right]\to M^{*}$ specifies a collision-free deformation that preserves the topology G and the connectivity structure $G_{M}$. Discrete grasping and releasing actions, in contrast, edit the graphs G and $G_{M}$ and trigger transitions between manifolds. Grasping actions are feasible only on submanifolds of $M^{*}$ where a gripper overlaps its target grip, while releasing actions are pure graph edits without modifying bending angles (Fig. 5A). Under this formulation, the MSRCR state space structure is represented as a locally infinite graph whose nodes are deformation manifolds and whose edges correspond to grasping and releasing actions. Figure 5A and movie S8 illustrate a six-module example, where distinct curves on a sampled deformation manifold drive the same initial configuration to different grasp-preparatory states that enable different grasping actions.

The planning problem is thus twofold. (i) task planning: search over the intermanifold transitions (grasp and release actions) to reconstruct the target’s angle system and outerface boundary permutation, i.e., reach the deformation manifold of $C_{0}^{\prime }$. (ii) motion planning: deform the configuration intramanifold to (a) bring a gripper and a grip into overlap for grasping or (b) match the target shape; we solve this continuous subproblem on the manifold using a sampling-based method.

#### Task planning via correspondence-based discretization and search

A unique discretization strategy tailored to the task planning problem is introduced. To address the locally infinite state-space graph due to continuous grasping angles, we discretize the action set using a “correspondence as discretization” scheme. This scheme partitions grasp actions into two finite classes: (i) constructive actions, whose grasping angle matches one in the target configuration and thereby establishes partial gripper correspondence, and (ii) auxiliary actions, whose grasping angles do not build correspondence but may be needed for feasibility. As a result, the planner simultaneously resolves grasping-angle discretization and angle correspondence formation within a unified planning process.

With this discretization, standard graph search algorithms can traverse the state-space graph across constraint manifolds toward a target manifold where all grasping angles of the target configuration are reconstructed. However, the lack of suitable admissible heuristic prevents the direct use of algorithms such as A*. Instead, we use a single-player Monte Carlo tree search (MCTS) framework for task planning. In the search tree, nodes are categorized by the action that generates them, namely, constructive, auxiliary, or releasing. Constructive nodes are further partitioned by the target grasping angle they instantiate. On the basis of this structure, we introduce a hierarchical node-grouping scheme (*58*) with a dedicated first-play urgency (FPU) design at each level (*59*). Through this hierarchical formulation, the correspondence-matching problem is reformulated as a correspondence-construction sequence decision problem and further as a grouped node selection priority problem that is well suited to be solved by MCTS. Last, because enumerating all feasible actions is computationally expensive and no explicit simulation model exists, we adopt an “expansion as simulation” strategy that evaluates nodes directly via their feasible next actions, thereby accelerating the search. We term this framework *HEART-MCTS*, an MCTS constructed on a hierarchical expansion-as-rollout tree. An example search tree expanded according to these rules is provided in fig. S9.

Enabled by HEART-MCTS, reconfiguration within the morphology and gait library is demonstrated in Fig. 5B, enabling transitions between distinct bioinspired functions. Reconfiguration between configurations outside the predefined library is shown in fig. S11, illustrating the planner’s ability to generalize beyond enumerated morphologies. Scalability of the planning framework is further demonstrated for systems of up to seven modules in movie S9.

To systematically benchmark the performance of proposed task planning algorithms, we develop a procedural generation framework for planar MSRCR configurations. In this framework, the mixed graph representation $G_{M}$ of an MSRCR configuration is first sampled using the configuration model (*60*). Cyclic orders and concrete geometric parameters are then assigned to obtain the final configuration C. As shown in Fig. 5C, the degree sequence used in the configuration model is sampled with a bias toward simpler configurations, reflecting their larger feasible domain for assigning geometric information, specifying grasping and bending angles. Using this framework, 400 pairs of configurations are procedurally generated to benchmark task planning, on which HEART-MCTS and the baseline method iterative auxiliary-budget breadth-first search (IAB-BFS; see baseline setup in Materials and Methods) are evaluated. As reported in Fig. 5D, HEART-MCTS outperforms IAB-BFS in planning efficiency, reducing average planning time by 59.5, 82.0, and 94.9% for three-, four-, and five-module cases. IAB-BFS is evaluated only on configurations with no more than five modules, as it becomes impractical to run on more complex instances. In terms of optimality, we measure task-plan length by the number of grasping actions because only grasping actions require preceding motion planning to bring grippers into overlap. Across 300 test cases with $N_{m}\in \left\{3,4,5\right\}$, HEART-MCTS attains an optimality rate of 97%, producing task plans with no more grasping actions than those generated by IAB-BFS.

### Hardware demonstration of reconfiguration and loco-manipulation

**Fig. 6.**
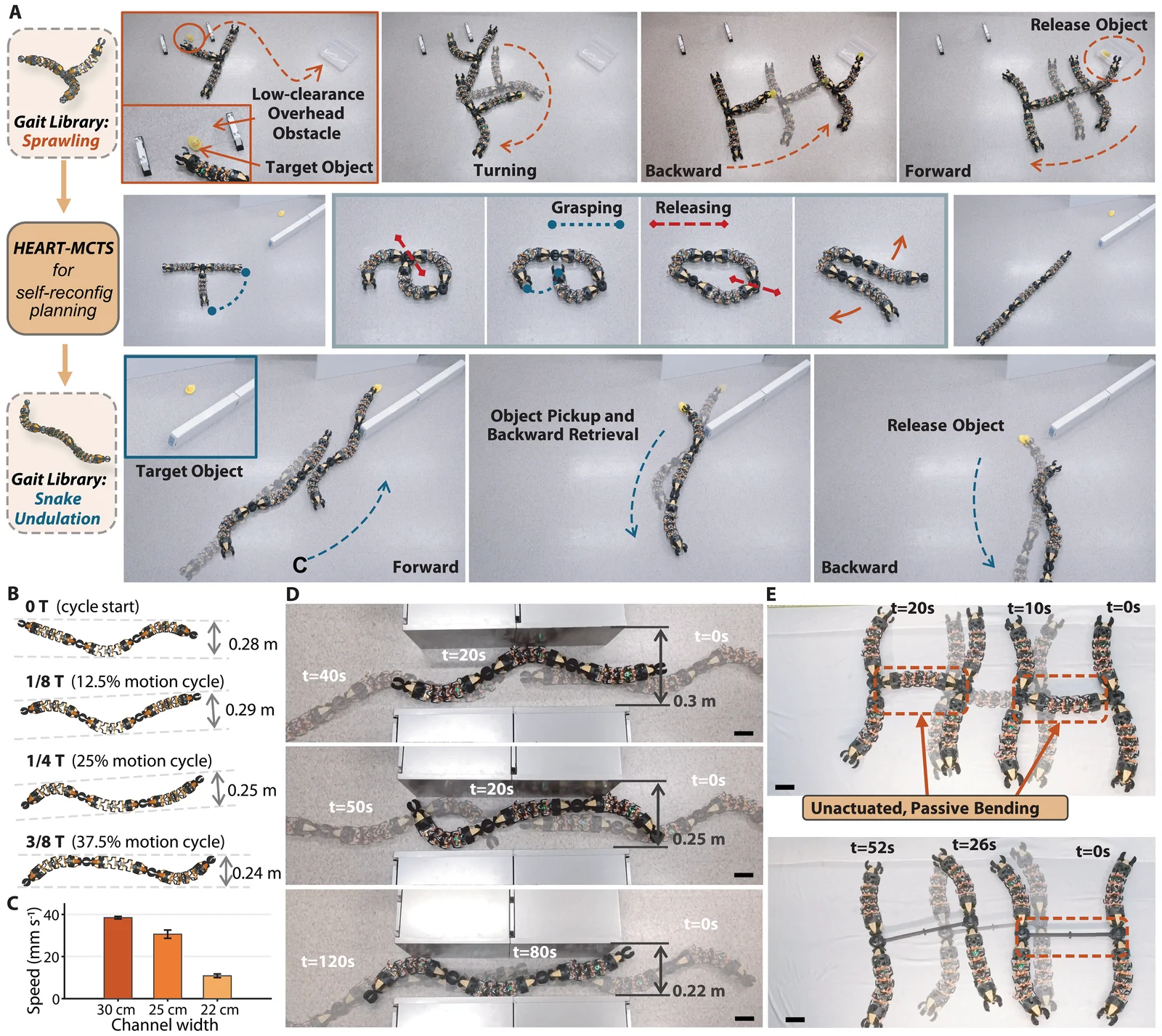
Hardware demonstrations of self-reconfiguration and loco-manipulation. (**A**) Locomotion-reconfiguration-locomotion sequence in which a three-module crawler reconfigures into a three-module snake to enter a narrow channel and reach a target object. (**B**) Time-lapse snapshots of one motion cycle during snakelike undulation, showing periodic body deformation, lateral wave propagation, and the corresponding lateral width of the gait. (**C**) Locomotion speed of the snakelike gait in channels with different widths. Error bars indicate the SD over three trials. (**D**) External compliance during snakelike locomotion through confined channels with widths of 30, 25, and 22 cm. (**E**) Internal compliance in a multimodule morphology, where an unactuated segment passively bends during crawling. Scale bars, 100 mm both in (D) and (E).

We demonstrate integrated reconfiguration and loco-manipulation behaviors on the physical robot using gaits selected from the gait library and reconfiguration plans generated by the proposed framework (Fig. 6A and movie S10). In Fig. 6A, a three-module sprawling morphology is selected for its suitability for downward reaching and upward lifting while maintaining efficient locomotion. The robot uses its distal tail module for grasping under low-clearance constraints and placing objects into a high container. The two-arm morphology generates efficient forward, backward, and turning motions under different tail states. After a self-reconfiguration sequence planned by HEART-MCTS, the three-module crawler transits to a snake through gripper releasing and grasping to access a narrowed channel. This self-reconfiguration process is executed on the basis of the offline planning results. Then, a snake-undulation gait enables the robot to enter the confined channel, grasp the object, and retrieve it through backward locomotion. The sequence of loco-manipulation highlights the ability to coordinate turning, bidirectional locomotion, and manipulation within a single morphology. Together, these hardware demonstrations show that adapting morphology and gait to task and environmental constraints enables robust loco-manipulation on a single untethered system.

#### Modular compliance for external and internal adaptation

We investigate the functional role of compliance in the MSRCR system by examining its effects on both external environment interaction and internal body coordination (Fig. 6, C to F, and movie S11). Compliance emerges as a unifying mechanism that enables robust locomotion in confined environments and effective force transmission across partially unactuated morphologies. In confined snakelike locomotion, the robot traverses channels with widths smaller than the nominal lateral undulation amplitude of the body (Fig. 6, C and D). As the channel width decreases from 30 to 22 cm, the forward speed decreases because of increased body-environment contact and constrained lateral motion. Nevertheless, the robot continues to translate through the channel, as the compliant backbone and intermodule joints passively deform to accommodate the channel boundaries, enabling locomotion despite insufficient kinematic clearance.

Internal compliance is highlighted in a four-limb sprawling configuration containing an unactuated, unpowered body module (Fig. 6F and movie S11). This passive body segment bends in response to forces generated by neighboring actuated limbs, redistributing contact forces and synchronizing limb-ground interactions across the body. A rigidized counterpart with identical morphology constrains interlimb motion, resulting in less aligned propulsion directions and increased foot slippage during stance. Consequently, the compliant configuration achieves a markedly higher forward speed (43.36 versus 15.8 mm·s^−1^). This mechanism mirrors the functional role of the vertebrate spine, where compliant axial structures coordinate limb forces to enable efficient whole-body propulsion (*61*). These results show that, in this tested MSRCR morphology, a passive compliant body segment can improve locomotion performance by mediating force transmission between actuated limbs.

## DISCUSSION

In this work, we presented a modular self-reconfigurable continuum robot that integrates compliant module design, quasi-freeform connectivity, and self-reconfiguration planning to support cross-species, multimodal loco-manipulation within a single platform. Inspired by evolutionary principles, the system embeds high–functional-density modules, pairs them with omnidirectional docking, and organizes them through a morphology-conditioned gait library to enable diverse locomotion strategies. Beyond hardware, we introduced a unified geometric-topological representation and a corresponding planning framework that allows the robot to systematically transition between configurations. Together, these contributions address two fundamental scientific questions: How compliance should be designed to maximize functional density at the module level, and how compliant modules should be organized and coordinated to achieve scalable morphology transitions and behavioral diversification.

The MSRCR module design combines a tendon-driven continuum backbone with omnidirectional docking grippers to prioritize adaptability and reconfigurability. A natural trade-off is reduced force transmission efficiency and localized deformation due to compliance, which limits speed and load capacity. Therefore, the current system is better suited for tasks that benefit from compliant contact and morphological adaptation, such as confined-space inspection rather than heavy payload transport or debris removal. Although stiffness variation (*62*) could improve load-bearing capability, incorporating it into a lightweight, self-contained, untethered modular system would introduce additional complexity. In the current design, stiffness can instead be adjusted in a modular manner by replacing the joint spring pair with varying geometry. Building on the capabilities demonstrated here, future work will extend MSRCR toward real field deployment in unstructured environments and other potential applications discussed below. This will require more reliable docking under misalignment and contamination, as well as morphology-selection strategies that adapt configurations and gaits to environmental constraints. Heterogeneous modules with varied length, stiffness, or functional specialization may further enable richer behaviors, stronger load bearing, and more animallike adaptability.

These future developments would support broader application scenarios where morphological adaptation must be combined with continuous structural integration. In confined-space inspection and search-and-rescue in collapsed environments, MSRCR could use different locomotion modes to traverse narrow gaps, irregular terrain, and uncertain contact. In underwater intervention and on-orbit assembly or servicing, connected reconfiguration could enable transitions between manipulation modes, from localized dexterous interaction to extended reach or cooperative grasping, while keeping the robot mechanically integrated and anchored. This continuity is particularly useful in environments where detached modules may drift under currents or in microgravity, making recovery, repositioning, and coordinated task execution difficult. Together, these scenarios highlight the potential of MSRCR as a reconfigurable continuum platform for adaptive locomotion and manipulation in complex environments.

In this work, we restrict the gait library and self-reconfiguration planning to planar configurations. Extending reconfiguration to fully nonplanar settings would require ensuring mechanical stability at every instant, including continuous verification of contact conditions, load support, and feasible docking angles, leading to a much more complex constraint space beyond the scope of this study. Notably, the proposed docking grippers already support spatial connections, providing a natural foundation for future extensions toward three-dimensional (3D) self-reconfiguration. However, beyond mechanical considerations, such an extension would also require a different spatial geometric-topological representation, as the current formulation relies on planar embedding constructs. While the underlying task-planning principles (e.g., correspondence-as-discretization, expansion-as-simulation, and hierarchical-node-grouping) and motion planning framework could in principle be extended to 3D, their realization would depend on the availability of such a representation.

Comparing MSRCR with representative modular soft robotic systems across tether dependence, reconfigurability, manipulation capability, module count, locomotion type, speed (table S3), and cost of transport (CoT) (table S4) reveals several key distinctions (*22*, *24*–*26*, *29*, *32*, *34*–*36*, *63*–*68*). Most existing systems exhibit only a subset of these attributes, while MSRCR is fully untethered and self-reconfigurable, supports manipulation, and realizes multiple locomotion modes. Its rolling gait achieves both competitive body-length–normalized speed and low CoT, whereas crawling and snakelike gaits show higher CoT because they involve intermittent contact, body dragging, or lateral sliding. These trade-offs should be interpreted in the context of functional objectives: MSRCR uses a chain-type morphology for mobile loco-manipulation and shape adaptation, while several modular soft robots use lattice-type architecture designed mainly for constructive assembly, load bearing, or shape forming (*35*, *65*). Moreover, the reported locomotion speeds for MSRCR do not reflect the mechanical limits of the structure. For safety and reliability, motor velocities were intentionally capped at ∼80% of the allowable limit during experiments. Unlike thermally or material-limited actuation mechanisms such as SMA-based systems, the tendon-driven architecture is not inherently speed limited by the structure and can readily achieve higher speeds with more powerful actuators.

The proposed HEART-MCTS is a heuristic, budget-limited task planner, whose coverage depends on the action discretization, search budget, and exploration parameters. The underlying motion planner, Atlas-RRT*, provides probabilistic completeness under its standard assumptions, and the overall framework can be extended to 3D by defining a corresponding 3D representation, action set, and collision model. In addition, the planning framework is not specific to MSRCR but is generally applicable to arbitrary planar chain-type mechanisms with rigid connections, enabling computation of self-collision–free configuration paths. Although explicit motion planning for kinematic reachability verification is avoided by relaxing reachability checks to a sequence of suboptimization problems that identify self-collision–free intermediate configurations (see the “Identifying feasible releasing and grasping actions” in Materials and Methods), this procedure remains computationally expensive and continues to suffer from the curse of dimensionality, as illustrated in Fig. 5D. To mitigate this limitation, a promising direction is to build upon the graph angle system (GAS)–GNN used as the angle-system encoder (see the “Task planning with HEART-MCTS” section in Materials and Methods) and develop a learned model that captures the full geometric information of configurations, enabling direct prediction of kinematic reachability between configurations without explicitly solving suboptimization problems.

## MATERIALS AND METHODS

### Manufacturing and design specifications

Each module’s backbone, including the yoke and cross disks, is 3D printed in polylactic acid (PLA). The V-shaped joint spring pairs are fabricated from thermoplastic polyurethane (TPU 95A), with detailed geometry shown in fig. S1. The backbone incorporates four U joints, each capable of bending more than 45°, allowing up to 180° total bending. Stiffness and backbone length can be customized by replacing the V-shaped spring pairs or adjusting the number of U joints. Cyclic bending tests showed that the TPU V-shaped spring pair maintained functional stiffness and 2-df bending performance after 5000 tendon-driven cycles within the locomotion-relevant bending range, with no visible cracks, tearing, or delamination (fig. S13). The backbone’s actuation tendon used nonelastic braided fish line (KastKing), which provides high tensile strength of up to 80 pounds with negligible deformation. Tendon shortening and lengthening are driven by 3D-printed pulleys with a 6-mm diameter mounted on the servo motor shafts. Each pulley has two layers that route antagonistic tendon pairs in opposite directions to enable bidirectional shortening and lengthening. As for the docking gripper, all components are printed using tough resin (ELEGOO Inc.) to ensure high toughness. With the grippers fully open, their outer edges align with the module’s outer circumference and become tangent to the ground plane, providing a stable resting surface. The gripper shells were lined with a commercially available elastic rubber band (AIWOQI #94, thickness: 1.5 mm), which provides a compliant backing layer for conformal closure, while the Velcro interface provides the primary load-bearing contact. The detailed design specifications are shown in fig. S1 and table S1.

### Electronics

The robot’s main computation runs on a Raspberry Pi Zero 2 W, which serves as the central processor for sensing, actuation, and communication. A custom printed circuit board (PCB) mounts directly onto the Pi’s GPIO header and provides the required power regulation and interface circuitry (fig. S2B). The board includes a power conditioning module that steps the 12-V battery input down to clean 5- and 3.3-V rails using a high-efficiency buck converter, followed by a low-noise linear regulator. Eight rotary angle sensors (CJMCU-103) are embedded along the robot backbone and wired in a chained configuration, with all sensor outputs routed to an analog multiplexer on the PCB (fig. S2C). The multiplexer sequentially selects each channel and feeds it into a 16-bit analog-to-digital converter (ADC). The ADC communicates with the Raspberry Pi through the serial peripheral interface (SPI) bus, allowing the Pi to acquire all eight angles through a single high-resolution conversion pathway. A nine-axis inertial measurement unit [(IMU) WT901, WitMotion] is connected directly to the Pi through the shared inter-integrated circuit (I2C) bus and provides real-time inertial feedback for state estimation. The regulated 5-V rail powers the four motors, including two for compliant backbone actuation and two for end-gripper actuation (DYNAMIXEL XC330-M288-T, ROBOTIS), which are commanded from the Raspberry Pi through a USB-tty interface.

### Control architecture and communication pipeline

Each module operates as a networked mechatronic unit with onboard sensing, low-level actuation, and a lightweight interface (fig. S2). The control program listens to the command from the personal computer (PC) through a transmission control protocol (TCP) client, which contains the desired motor velocity for the four DYNAMIXEL motors. It then reads the position and current of each motor, along with the eight angle sensors for shape sensing, and the IMU data for base orientation identification. At each cycle, the module transmits a compact sensor packet to the PC, containing a feedback signal of bending angles, IMU values, and per motor position and current.

A master control program on the host PC manages communication with all modules through a multithreaded TCP server. The server identifies each module by its internet protocol (IP) address and assigns a dedicated handler to manage bidirectional communication. Each handler receives feedback data from the module, displays them in the interface, and sends velocity or gripper commands back to the module. In autonomous locomotion mode, a CPG synthesizes sinusoidal oscillator signals for a selected gait and module count, converts them into tendon-specific motor velocities, and updates a global velocity vector at 20 Hz. In the manual control mode, manual commands generated from the graphical user interface (GUI) use the same communication pathway, allowing the user to operate motors and grippers for testing or teleoperation. Both the autonomous mode and the manual mode are integrated into a control GUI as shown in fig. S4.

### Simulation environment

We implemented a physics-based simulation of the MSRCR system in MuJoCo (*69*) for evaluating locomotion gait parameters and reconfiguration planning results. Each tendon in the continuum module was modeled as a MuJoCo tendon element, with actuation specified in either length or velocity space to match the hardware control abstraction. Compliant universal joints were modeled by assigning stiffness to the revolute joints. Contact interactions between the robot, ground, and docking grippers were handled using MuJoCo’s compliant contact model, while docking states were encoded as kinematic constraints between spherical connectors. Locomotion performance was evaluated by executing coupled CPG trajectories and measuring forward displacement and gait feasibility over fixed horizons, and planned reconfiguration sequences were replayed to verify kinematic feasibility and collision-free execution.

### Unified CPG controller for multimorphology gaits

#### Network formulation

To generate rhythmic actuation across different robot morphologies, we use a unified CPG network that encodes both spatial and temporal coordination. The same controller structure supports rolling, snakelike undulation, crawling, and quadruped walking by adjusting only the oscillator connectivity and the phase biases. Each continuum module contains two phase oscillators that govern bending in the orthogonal X and Y directions. The network evolves according to a Kuramoto type formulation

$${\dot{\theta }}_{i}=\omega_{i}+\sum_{j\in 𝒩\left(i\right)}k_{\mathrm{ij}}\;\text{sin}\left(\theta_{j}-\theta_{i}-\phi_{\mathrm{ij}}\right)$$ (4)

where $\omega_{i}$ is the intrinsic frequency, $k_{\mathrm{ij}}$ is the coupling strength, $\phi_{\mathrm{ij}}$ is the desired phase bias, and N(i) is the set of neighbors of oscillator i.

Equation 4 synchronizes the oscillator network into a stable phase-locked pattern. Within each module, the phase relation between the local X- and Y-bending oscillators determines the module-level bending pattern: Single-axis or synchronized commands generate planar bending, such as forward/backward or upward/downward bending, whereas a phase-shifted pair generates a rotating bending direction. Across modules, the phase biases define the coordination pattern between modules. A spatial phase gradient produces traveling waves, as used in snakelike locomotion, while symmetric or matched phase biases produce standing-wave, in-phase, or alternating support patterns for crawling and walking-like gaits. Thus, the same CPG network can represent multiple gait families by changing the within-module and between-module phase structure.

#### Oscillator output mapping

Each CPG oscillator generates a sinusoid with fixed amplitude and evolving phase. For bending axis b∈{X,Y} of module i, the commanded bending displacement in this axis is

$$x_{i,b}\left(t\right)=x_{i,b}^{0}+A_{i,b}\text{sin}\theta_{i,b}\left(t\right)$$ (5)

where $x_{i,b}^{0}$ is a static offset and $A_{i,b}$ is the oscillation amplitude. Differentiating yields the desired velocity for that axis

$${\dot{x}}_{i,b}\left(t\right)=A_{i,b}\omega_{i,b}\text{cos}\theta_{i,b}\left(t\right)$$ (6)

These velocities $\left({\dot{x}}_{i,X},{\dot{x}}_{i,Y}\right)$ constitute the rhythmic bending command in the local *X*/*Y*- bending frame. Because the tendons of each module are routed at a geometric offset $\psi_{i}$ relative to the *X*/*Y* axes, the oscillator outputs are rotated into the tendon coordinate frame

$$\left[\begin{array}{c} {\dot{l}}_{i,1}\\ {\dot{l}}_{i,2} \end{array}\right]=R\left(\psi_{i}\right)\left[\begin{array}{c} {\dot{x}}_{i,X}\\ {\dot{x}}_{i,Y} \end{array}\right],R\left(\psi_{i}\right)=\left[\begin{array}{cc} \text{cos}\psi_{i} & \text{sin}\psi_{i}\\ -\text{sin}\psi_{i} & \text{cos}\psi_{i} \end{array}\right]$$ (7)

The resulting tendon velocities ${\dot{l}}_{i,1},{\dot{l}}_{i,2}$ are then converted to raw motor velocity commands by ${\dot{q}}_{i,k}=\frac{{\dot{l}}_{i,k}}{s_{i,k}}$, where $s_{i,k}$ is the transmission ratio indicating how much tendon length change is produced per unit motor rotation. These motor velocity commands ${\dot{q}}_{i,k}$ are sent directly to the actuators. Then, the controller is lastly implemented and runs at 100 Hz on the host PC. At each step, Eq. 4 is integrated using explicit Euler

$$\theta_{i}\left(t+\Delta t\right)=\theta_{i}\left(t\right)+\left[\omega_{i}+\sum_{j}k_{\mathrm{ij}}\text{sin}\left(\theta_{j}-\theta_{i}-\phi_{\mathrm{ij}}\right)\right]\Delta t$$ (8)

The tendon signals are then computed using Eqs. 5 to 7, and the tendon velocities are sent to the actuators.

#### CPG parameter tuning

For each morphology and gait, we first selected the phase biases from the predefined animal-inspired gait template. With the phase structure fixed, we tuned the oscillation amplitudes in simulation and hardware tests to improve locomotion speed while keeping the tendon speed within motor limits and avoiding unstable contact. This manually designed gait parameter aims to preserve clear correspondence between morphology, gait, and biological inspiration while treating numerical optimization as a tool for future template-constrained parameter refinement. The detailed CPG parameters for all the different morphologies and gaits are shown in table S2.

### MSRCR self-reconfiguration planning algorithm

We now present the formal problem definition and algorithmic details for MSRCR self-reconfiguration planning.

#### Definitions of correspondence and deformation manifold

Some correspondence $f_{c}:P_{0}\to P_{1}$ is required during self-reconfiguration planning. This correspondence $f_{c}$ is a bijection established between grippers in the initial and target configurations while preserving pair-wise relation between head and tail, i.e., $f_{c}\circ \alpha =\alpha \circ f_{c}$. Under this correspondence, using the rigid transform T from the definition of shape equivalence, the graph embeddings coincide at the gripper level such that $T\left[S_{0}^{\prime }\left(p_{0}\right)\right]=S_{1}\left[f_{c}\left(p_{0}\right)\right]$, for all $p_{0}\in P_{0}$. Specifically, we denote that $C_{0}^{\prime }$ and $C_{1}$ are shape equivalent under $f_{c}$, written $C_{0}^{\prime }{∼}_{s|f_{c}}C_{1}$. Moreover, all configurations that are kinematically equivalent to $C_{0}^{\prime }$ are regarded as kinematically equivalent to $C_{1}$ under $f_{c}$, forming the equivalence class ${\left[C_{1}\right]}_{k|f_{c}}$.

For a given configuration $C^{*}$, its kinematically equivalent class ${\left[C^{*}\right]}_{k|I}$ under identity correspondence forms a constraint manifold $M^{*}\subset {\mathbb{R}}^{N_{m}}$, parameterized by the corresponding bending-angle vectors. We name this constraint manifold the deformation manifold of a configuration. Under identity correspondence, each gripper maps to itself during deformation. Consequently, all configurations on $M^{*}$ are reachable to each other via continuous deformation and share the same topological structures $G^{*}$, $G_{M}^{*}$, angle system $A^{*}$, and outerface boundary permutation $\pi_{\infty }^{*}$. Thus, each point $x\in M^{*}$ specifies a configuration, and each curve $c:\left[0,1\right]\to M^{*}$ specifies a deformation.

#### Formal problem definition

Building on the problem formulation and state-space structure introduced in Results, we now present the precise operational definition of the MSRCR self-reconfiguration planning problem and the action representations used by the proposed algorithms. Given an initial configuration $C_{0}$ and a target configuration $C_{1}$, the self-reconfiguration planning problem seeks a sequence of valid actions

$$A=\left(a_{0},a_{1},\ldots ,a_{T}\right)$$ (9)

such that applying all actions in A to $C_{0}$ yields a configuration $C_{0}^{\prime }$ that is shape equivalent to the target, i.e., $C_{0}^{\prime }{∼}_{s}C_{1}$. Each action $a_{t}$ is classified as one of three types, releasing, deforming, or grasping, and is represented with arguments appropriate to its effect on the configuration.

A releasing action, written as $a^{r}\left(p\right)$, specifies that gripper p releases its current grasp, thereby removing the grasping relation $q\;\leq_{S}\;p$ for some gripper q. A deforming action, denoted $a^{d}\left(\tau \right)$, applies a valid bending-angle trajectory τ on the constraint manifold of the present configuration, representing a continuous deformation process. A grasping action, expressed as $a^{g}\left(\pi ,s,\gamma \right)$, is defined by a path $\pi =\left(p_{i_{0}},p_{i_{1}},\ldots ,p_{i_{k}}\right)$ in the mixed graph $G_{M}$, an orientation indicator s∈{−1,1}, and a grasping angle γ. Executing this action causes gripper $p_{i_{0}}$ to grasp $p_{i_{k}}$ with grasping angle γ, converting the path π into a cycle whose ordering is counterclockwise when s=1 and clockwise when s=−1.

Deforming actions are required only in two cases: first, as a grasping-enabling deformation to continuously deform the configuration so that two grippers overlap in preparation for an immediate grasping action later in the sequence; and second, as the final deformation to deform the configuration so that it becomes shape equivalent to the target configuration, in which case the deforming action must be the terminal action $a_{T}$. Recall that the MSRCR state space is modeled as a locally infinite graph with constraint manifolds as vertices and grasping and releasing actions as edges, and the deforming actions correspond to motion within each manifold. The self-reconfiguration planning problem is therefore decomposed into two parts: task planning, which finds a sequence of releasing and grasping actions that brings the system to a configuration kinematically equivalent to the target, and motion planning, which computes the bending-angle trajectories for both grasping-enabling deformations and realizing the final deformation.

#### Identifying feasible releasing and grasping actions

We now describe how feasible actions are identified for a configuration C. A releasing action $a^{r}\left(p\right)$ is valid only if two conditions are satisfied. First, the releasing gripper p must be the outermost gripper in its grip, meaning it is not grasped by any other gripper; equivalently, there exists no q∈P such that $p\leq_{S}\;q$. Second, removing the grasping relation at p must not disconnect the configuration, i.e., the corresponding directed grasping edge must not be a bridge in the mixed graph $G_{M}$. In practice, we perform a depth-first search (DFS) to identify all such outermost grippers whose release would not break connectivity, thereby producing the set of feasible releasing actions.

Feasible grasping actions, on the other hand, are more difficult to identify, because a rigorous check would require motion planning to confirm the existence of a preceding deforming action with a valid bending-angle trajectory τ that brings the relevant grippers into overlap before establishing the corresponding grasping relation. Exhaustively performing such checks to enumerate all feasible grasping actions during task planning is computationally impractical. Instead, in practice, we first perform a BFS on $G_{M}$ to enumerate candidate paths π, with the orientation s∈{−1,1} and grasping angle γ drawn from the discretized angle set. For each candidate grasping action $a^{g}\left(\pi ,s,\gamma \right)$, we then solve an optimization problem over the bending angles B, which returns either a self-collision–free, gripper-overlapping configuration with bending angles τ(1) that enables the grasp or failure. In this way, the problem is reduced from searching for a full trajectory τ via motion planning to checking the existence of a single feasible end configuration τ(1), which is substantially faster. This condition, however, is necessary but not sufficient to guarantee the feasibility of the grasping action. Therefore, we adopt a lazy verification strategy in which full motion planning is performed after task planning has produced a candidate sequence of grasping and releasing actions.

The above optimization problem, subject to single-module forward kinematics and self-collision–free constraints, is the most computationally intensive component of the overall planning framework. In practice, we accelerate this step by parallel execution across multiple central processing unit (CPU) cores, evaluating candidate optimization problems independently.

#### Task planning baseline setup

As a baseline, we implement a BFS planner under the proposed correspondence-as-discretization scheme. To prevent redundant exploration through auxiliary actions in standard BFS, we introduce an auxiliary-budget $\eta_{\mathrm{AB}}$, which limits the number of such actions permitted on any search branch. When $\eta_{\mathrm{AB}}=0$, auxiliary actions are disabled, and only constructive actions are considered. In practice, we perform BFS repeatedly while iteratively increasing $\eta_{\mathrm{AB}}$, analogous to iterative deepening DFS. This approach is referred to as IAB-BFS.

#### Task planning with HEART-MCTS

Task planning for MSRCR self-reconfiguration seeks a sequence of releasing and grasping actions that transform an initial configuration into a configuration that is kinematically equivalent to the target. Given the set of feasible actions at each search node, we expand the node by enumerating all admissible successor actions and apply a single-player MCTS procedure over the resulting action tree. To exploit the distinct roles of constructive grasping, auxiliary grasping, and releasing actions, we organize the node expansion hierarchically. At the first level, child nodes are grouped by action type (constructive, auxiliary, or releasing). At the second level, constructive-action children are further partitioned according to their target grasps under the correspondence-as-discretization scheme. This hierarchical grouping allows the search policy to first allocate exploration among action types and, when selecting a constructive action, among the target grasps to be realized next. By assigning dedicated FPU values to different groups at each level, we flexibly regulate the exploration-exploitation balance at both levels of the search. A schematic overview of the planning algorithm is provided in fig. S10.

In addition to the hierarchical tree structure, HEART-MCTS adopts an expansion-as-simulation strategy. Conventional rollout simulation is inappropriate in our setting: Simulating action sequences to termination is computationally expensive, and node evaluation is further hindered by the absence of an admissible distance estimator between an intermediate configuration and the target. We therefore replaced rollouts with direct node evaluation based on one-step expansion. Specifically, we characterize a node by the group types of its reachable child nodes (constructive, auxiliary, or releasing), using this information as a proxy for evaluating the node’s promise in progressing toward the target. In parallel, we quantify task-level progress by the number of target grasping angles established along the path to the node. The node value Q is then computed as a weighted sum of the promise score and the progress score and is used as the exploitation term in the upper confidence bound criterion for node selection in MCTS. HEART-MCTS terminates when the task planner has instantiated all target grasping angles, i.e., when the constructive-action sequence has reconstructed the complete target angle system.

To prevent redundant exploration, we suppress revisits by identifying previously encountered search states at the level of constraint manifolds (kinematic-equivalence classes). Because exact kinematic-equivalence testing is expensive, we use the necessary structural criterion introduced above and use the angle system of a configuration as a practical identifier for its equivalence class. To implement this efficiently, we develop GAS-GNN, a GNN designed to encode the GAS of an MSRCR configuration into a compact representation. GAS-GNN uses a sequence-sensitive recurrent neural network (RNN) for both neighborhood aggregation and global pooling, allowing it to capture the cyclic-order information that defines the rotation system together with grasping angles as geometric attributes, thus producing an index-invariant encoding. During search, configurations that map to the same angle-system encoding are treated as duplicates and are not reexpanded, thereby enforcing revisit prevention throughout HEART-MCTS

#### Motion planning on identified constraint manifolds

Motion planning for MSRCR self-reconfiguration is performed after task planning, once an action sequence A has been determined. As discussed above, the bending-angle vector B corresponding to configurations within the same kinematic equivalence class forms a constraint manifold. We identify this manifold by enforcing two classes of constraints: (i) angle-system consistency and (ii) self-collision avoidance. Self-collision avoidance is handled using polygonal colliders that approximate module segments, together with collision checks during manifold evaluation.

Enforcing angle-system consistency requires maintaining loop closure constraints induced by cycles in the configuration graph G. For each initial configuration, we first compute a minimum cycle basis using Johnson’s algorithm (*70*). This cycle basis is then updated after each grasping or releasing action in the action sequence A, ensuring that the active constraints remain consistent with the evolving graph topology. For a configuration whose graph contains $N_{c}$ independent cycles, we impose $3N_{c}$ scalar constraints by requiring each cycle to close from tail to head in planar pose. These constraints are constructed by composing per-module kinematics along a directed walk (clockwise or counterclockwise) around each cycle. The traversal order is determined from the known embedding information of the configuration, including the outerface boundary permutation $\pi_{\infty }$ and the rotation system R; in practice, we recover the corresponding cycle traversal order via BFS on this combinatorial structure.

Once all loop-closure constraint equations are defined, the constraint manifold is locally characterized through their associated Jacobians. We then apply Atlas-RRT* (*71*, *72*), a sampling-based anytime planner, to compute a continuous trajectory on the manifold connecting the start and end configurations. Atlas-RRT* first searches for an initial feasible path and then continues to refine the path if additional computation time is available. In our experiments, we used a 20-s post-feasibility refinement cap under a uniform stopping condition. This value was chosen to standardize solution refinement rather than to reflect the time required for finding feasibility; in feasibility-only benchmarks, initial feasible paths were found in 1.2 ± 0.7 and 1.5 ± 1.2 s for five- and seven-module systems, measured more than 99 and 97 randomly generated motion planning pairs, respectively.

### Result reproduction for self-reconfiguration planning

The code necessary to reproduce the self-reconfiguration planning results here has been deposited in the Zenodo database https://doi.org/10.5281/zenodo.20215519 and https://github.com/JanusMaple/CMRRP. All evaluation experiments were conducted on a computer with a 24-core Intel Core Ultra 9 285K CPU and 64 gigabytes of RAM.

## Supplementary Materials

### The PDF file includes:

Supplementary Text Figs. S1 to S13 Tables S1 to S4 Legends for movies S1 to S12 References
