# Supplementary material for: Evolutionary diversification via modular compliance for self-reconfigurable continuum robots
Source: Sci Adv. 2026 Aug 19;12(34):eaeg9191. doi: 10.1126/sciadv.aeg9191 (PMC13488886; doi:10.1126/sciadv.aeg9191)
Supplement: Supplementary file 1 [file sciadv.aeg9191_sm.pdf]

Science Advances
AAAS

Supplementary Materials for

Evolutionary diversification via modular compliance for self-reconfigurable continuum robots

Yilin Cai *et al.*

Corresponding author: Yue Chen, yue.chen@bme.gatech.edu

Sci. Adv. **12**, eaeg9191 (2026)
DOI: 10.1126/sciadv.aeg9191

The PDF file includes:

Supplementary Text
Figs. S1 to S13
Tables S1 to S4
Legends for movies S1 to S12
References

Other Supplementary Material for this manuscript includes the following:

Movies S1 to S12

Supplementary Text

Teleoperation control

Manual teleoperation was implemented as configuration-space bending control of a selected module. The selected module has two bending degrees of freedom, corresponding to bending along its local x- and y-axes. In the GUI, the arrow buttons send single-axis velocity commands for pure bending along the local x or y direction. In the pick-and-place demonstration, a joystick was used to provide a continuous two-dimensional input vector $\mathbf{u} = [u_x, u_y]^T$, which specifies the desired bending direction.

To avoid unintended motion caused by joystick noise, a dead zone was applied to the input. The joystick vector was mapped to the commanded bending velocity as

$$\mathbf{v}_b = \begin{cases} v_0 \dfrac{\mathbf{u}}{\| \mathbf{u} \|}, & \| \mathbf{u} \| > \epsilon, \\ \mathbf{0}, & \| \mathbf{u} \| \leq \epsilon, \end{cases}$$

where $\mathbf{v}_b = [\dot{\theta}_x, \dot{\theta}_y]^T$ is the commanded bending velocity, v_0 is the fixed bending-speed magnitude, and ϵ is the joystick dead-zone threshold. Thus, once the joystick input exceeds the threshold, the joystick direction determines the bending direction, while the module bends at a fixed speed.

Because the tendon routing is rotated by $45°$ relative to the module x- and y-bending axes, the desired bending velocity was converted to tendon-pair motor commands using a fixed rotation mapping,

$$\mathbf{v}_m = R(45°)\mathbf{v}_b, \quad R(45°) = \begin{bmatrix} \cos 45° & -\sin 45° \\ \sin 45° & \cos 45° \end{bmatrix}$$

where $\mathbf{v}_m = [v_{m1}, v_{m2}]^T$ represents the velocity commands sent to the two tendon-pair motors. This mapping allows the operator to command intuitive bending directions while the software converts them into the corresponding motor velocities. In the reported pick-and-place experiment, one module was selected and controlled at a time, which was sufficient for the demonstrated manipulation task. For multi-module manipulation, the same interface can be extended to end-effector control using forward or inverse kinematics, so coordinated module motions can be generated automatically from desired task-space commands.

Durability Test of the Joint Spring Pair

To evaluate the durability of the TPU springs under repetitive bending, we conducted a cyclic bending test using the same tendon-driven actuation setup as used in the robot module. The motor was commanded to repeatedly pull and release the tendon, causing the TPU spring to bend back and forth over the tested angular range. After 1000 and 5000 bending cycles, the spring was

removed from the cyclic test and characterized using the same static loading procedure as the initial condition. The experimental setup and results are shown in Fig. S12.

For each condition, three repeated loading trials were performed. Known weights were applied to the tendon-driven structure, and the corresponding bending angle was measured. The applied force is F and the bending moment was estimated as

$$M = Fl\cos\left(\frac{\theta}{2}\right),$$

where $l = 29.49$ mm is the effective moment arm and θ is the measured bending angle. The moment-angle response was nonlinear, so the data were fitted using a quadratic model,

$$M(\theta) = a\theta^2 + b\theta + c.$$

The effective bending stiffness was then computed from the derivative of the fitted moment-angle curve,

$$K(\theta) = \frac{dM}{d\theta} = 2a\theta + b.$$

The moment-angle response of the TPU joint spring pair was nonlinear across all tested conditions, and the quadratic model consistently captured the response better than the linear model. The initial response was fitted with $R^2 = 0.946$ using the quadratic model, compared with $R^2 = 0.921$ for the linear model. After cyclic loading, the quadratic fit further improved to $R^2 = 0.984$ after 1000 cycles and $R^2 = 0.992$ after 5000 cycles, indicating that the spring retained a smooth and repeatable nonlinear response after repeated bending.

The effective bending stiffness showed an initial settling behavior after cyclic loading, especially at small bending angles. At $10°$, the stiffness decreased from 0.299 N·m·rad^{-1} initially to 0.112 N·m·rad^{-1} after 1000 cycles and 0.123 N·m·rad^{-1} after 5000 cycles. At larger bending angles, the stiffness remained comparable to the initial condition. For example, at $35°$, the stiffness changed from 0.837 N·m·rad^{-1} initially to 0.931 N·m·rad^{-1} after 1000 cycles and 0.885 N·m·rad^{-1} after 5000 cycles. These results suggest that most mechanical settling occurs during early cycling, after which the spring response remains stable. Visual inspection after 5000 cycles showed no cracks, tearing, delamination, or permanent structural damage of the TPU spring. Therefore, although the moment-angle response exhibited limited cycle-induced settling, the spring pair maintained its nonlinear bending behavior without visible material failure or catastrophic stiffness loss.

In the robot, bending is generated by commanded tendon displacement rather than by a prescribed external load. Therefore, moderate changes in passive stiffness mainly affect the required tendon force or motor load, while the quasi-static bending angle remains governed by the tendon length command. The 5000-cycle test therefore provides an extended durability validation within the bending range required for the demonstrated locomotion gaits. The TPU joint spring pair maintained functional stiffness and structural integrity under repeated bending to approximately $\pm 30°$, which covers the typical deformation range during locomotion. Although the ultimate

fatigue life was not reached, the results indicate reliable performance within the locomotion-relevant loading range tested here.

Contamination Tolerance of the Velcro-Based Docking Interface

To evaluate the effect of environmental contamination on the Velcro-assisted docking interface, we measured the pulling force sustained by two docked grippers under five surface conditions: clean, water, grass, sand, and leaf debris (Fig. S13). Water, grass, and sand were applied directly to the gripper/Velcro contact surface. Leaf debris was placed between the two gripper shells, blocking direct shell-shell contact and representing a more severe case in which the gripper shell contact is partially disabled.

For each condition, two grippers were docked at a relative angle of 0°, and the connected pair was pulled using a force gauge until slip. The peak pulling force was recorded over five trials. The clean interface sustained a pulling force of 54.7 ± 2.7 N. The contaminated interfaces retained measurable holding force in all tested cases: water, 56.1 ± 2.8 N; grass, 49.6 ± 4.6 N; sand, 44.1 ± 3.7 N; and leaf debris, 32.4 ± 4.6 N. These values correspond to approximately 102.5%, 90.7%, 80.6%, and 59.1% of the clean condition, respectively.

These results show that the Velcro-assisted docking interface remains functional under the tested contaminated conditions. Water had little effect on the holding force, while grass and sand caused moderate reductions, likely by reducing the effective Velcro engagement area. Leaf debris caused the largest reduction because it physically blocked contact between the gripper shells. Nevertheless, even in this severe blockage condition, the docked interface retained a pulling force above 30 N. This indicates that surface contamination reduces the available holding force but does not immediately compromise the mechanical docking capability under the tested conditions.

CoT Comparison and Discussion

To quantify locomotion energy efficiency, we calculated the dimensionless cost of transport (CoT) for each gait as

$$\mathrm{CoT} = \frac{E}{mgd}$$

where E is the total electrical energy consumed during locomotion, m is the robot mass, g is the gravitational acceleration, and d is the traveled distance. Equivalently, when using average electrical power $\bar{P}$ and average locomotion speed v,

$$\mathrm{CoT} = \frac{\bar{P}}{mgv}$$

For MSRCR, the average electrical power was estimated from motor-side power consumption. During each gait, we measured the current of the active tendon motors and used the constant supply voltage V to compute the instantaneous system input power as

$$P(t)=\sum_{i=1}^{N_m} V\, I_i(t),$$

where N_m is the number of active tendon motors and $I_i(t)$ is the current of the i-th motor. The average electrical power was then computed over the recording interval as

$$\bar{P}=\frac{1}{T}\int_0^T P(t)dt=\frac{1}{T}\int_0^T \sum_{i=1}^{N_m} V\, I_i(t)dt.$$

In discrete implementation, this was approximated as

$$\bar{P}\approx\frac{1}{T}\sum_{k=1}^{N}\sum_{i=1}^{N_m} V I_i(t_k)\Delta t.$$

The CoT comparison in Table S4 shows that MSRCR achieves low transport costs among recent soft modular and reconfigurable robotic systems while retaining multiple functions. Within MSRCR, rolling is the most energy-efficient gait among the measured modes, with CoT values of 1.20 for the single-module robot and 1.17 for the three-module robot. Crawling and snake-like locomotion have higher CoT values of 3.39 and 9.54, respectively. In the three-module crawling gait, only two modules act as arms while the third has basically zero energy input. Snake-like locomotion has the highest CoT, mainly due to lateral sliding and low forward displacement per cycle. Compared with recent soft modular systems (Table S4), MSRCR shows substantially lower CoT.

However, CoT comparisons should be interpreted with caution because different studies define and measure energy input differently. Some systems use electrical input power, while others normalize cable actuation energy, elastic energy changes, or power-to-weight ratios. Therefore, Table S4 is intended to provide a contextual comparison among recent soft modular and reconfigurable robots, rather than a strictly normalized benchmark across all robotic platforms.

Angle-System-Preserving Isotopy for Kinematic Equivalence Verification

As stated in the main text, we regard any continuous deformation of an MSRCR configuration as a special angle-system-preserving isotopy. Consider a deformation that transfers $\mathcal{C}_0=\left(M_0,P_0,G_0,\leq_{S_0},B_0,A_0,\pi_\infty^{(0)}\right)$ to $\mathcal{C}_1=\left(M_1,P_1,G_1,\leq_{S_1},B_1,A_1,\pi_\infty^{(1)}\right)$, driven by a continuous bending angle trajectory $\tau:[0,1]\to\mathbb{R}^{N_m}$, where $N_m=|M_0|=|M_1|$, $\tau(0)=B_0$ and $\tau(1)=B_1$. Because bending angle variations do not create or break gripper connections, the underlying graph

representation of the configuration remains unchanged; that is, $\mathcal{G}^* = \mathcal{G}_0 = \mathcal{G}_1$. A trajectory τ is called valid if every intermediate configuration $\mathcal{C}_t$ at time $t \in [0,1]$ satisfies the following two conditions: the shape map $\mathcal{S}_t: \mathcal{G}^* \to \mathbb{R}^2$ is an embedding for all $t \in [0,1]$, ensuring self-collision avoidance; and the angle system remains unchanged, $A_t \equiv A_0$, reflecting the locked gripper connections. As the configuration shape varies continuously from $\mathcal{S}_0$ to $\mathcal{S}_t$, the motion induces, for each t, a homeomorphism $h_t: \mathcal{S}_0(\mathcal{G}^*) \to \mathcal{S}_t(\mathcal{G}^*)$ such that $h_t \circ \mathcal{S}_0 = \mathcal{S}_t$. The family $\{h_t\}_{t\in[0,1]}$ therefore defines an isotopy $H: [0,1] \times \mathcal{S}_0(\mathcal{G}^*) \to \mathbb{R}^2$, where $H(t,x) = h_t(x)$ with $H(0,\cdot) = id_{\mathcal{S}_0(\mathcal{G}^*)}$. Thus, a deformation of an MSRCR configuration is precisely an isotopy that preserves its angle system, i.e., an angle–system–preserving isotopy.

Remark. *Since the embedded configuration shape $\mathcal{S}_0(\mathcal{G}^*)$ is a planar continuum, the isotopy H defined above can be extended to an ambient isotopy of the plane by the isotopy extension theorem for planar continua (77). This ambient extension is not used in our framework but situates our construction within classical planar topology.*

We now show that an angle–system–preserving isotopy exists between two configurations $\mathcal{C}_0$ and $\mathcal{C}_1$ if and only if their angle systems coincide, and their outer face boundary permutations agree, up to correspondence. Specifically, there exists an angle-system–preserving isotopy between $\mathcal{C}_0$ and $\mathcal{C}_1$ if and only if there is a correspondence $f_c: P_0 \to P_1$ between their grippers such that

$$f_c(A_0) = A_1, \qquad f_c \circ \pi_\infty^{(0)} = \pi_\infty^{(1)}$$

Here, the action of f_c on an angle system A is simply a relabeling of all grippers according to f_c. In practice, equality of angle systems is evaluated using the index-invariant GAS-GNN encoding described in the main text, which obviates the need to explicitly enumerate correspondences. Without loss of generality, we therefore omit further explicit references to correspondences in the remainder of this section and assume $A_0 = A_1$ and $\pi_\infty^{(0)} = \pi_\infty^{(1)}$.

The **necessity** of the above claim is immediate. An angle-system–preserving isotopy, by definition, keeps every local grasping angle fixed throughout the deformation and therefore cannot change the discrete angle system; thus $A_0 = A_1$. Likewise, because the embedding evolves continuously through embeddings of the same graph in the plane, the isotopy cannot modify the cyclic ordering of edges around the unbounded face. Consequently, the outer-face boundary permutation must remain invariant, giving $\pi_\infty^{(0)} = \pi_\infty^{(1)}$.

We now turn to the **sufficiency** direction. Consider two configurations $\mathcal{C}_0$ and $\mathcal{C}_1$ sharing the same angle system and outer face boundary permutation, i.e.,

$$A_0 = A_1, \qquad \pi_\infty^{(0)} = \pi_\infty^{(1)}$$

Equality of angle systems implies equality of rotation systems, and therefore the underlying graphs $\mathcal{G}_0$ and $\mathcal{G}_1$ are combinatorially isomorphic; we write $\mathcal{G} = \mathcal{G}_0 \backsimeq \mathcal{G}_1$. Recall that the shapes $\mathcal{S}_0$ and $\mathcal{S}_1$ of two configurations are graph embeddings with an orientable, flat and infinite plane $\mathbb{R}^2$ as their codomain. One-point compactification of this plane identifies $\mathbb{R}^2 \cup \{\infty\}$ with the sphere $\mathbb{S}^2$, so each configuration induces an embedding

$$\mathcal{S}_0': \mathcal{G} \to \mathbb{S}^2, \qquad \mathcal{S}_1': \mathcal{G} \to \mathbb{S}^2.$$

Because the two embeddings have the same rotation system, the Heffter-Edmonds-Ringel Rotation Principle *(78)* guarantees the existence of orientation preserving homeomorphism

$$h: \mathbb{S}^2 \to \mathbb{S}^2 \text{ with } h \circ \mathcal{S}_0' = \mathcal{S}_1'$$

Since every orientation-preserving homeomorphism of $\mathbb{S}^2$ is isotopic to the identity *(79)*, there exists an ambient isotopy

$$\widetilde{H}: [0,1] \times \mathbb{S}^2 \to \mathbb{S}^2 \text{ with } \widetilde{H}(0,\cdot) = id_{\mathbb{S}^2}, \widetilde{H}(1,\cdot) = h$$

For each vertex $v \in \mathcal{G}$, let

$$\zeta_v(t) = \widetilde{H}\big(t, \mathcal{S}_0'(v)\big)$$

denote the trajectory of the embedded vertex under $\widetilde{H}$. Choose a closed disk $D_v^{(0)} \subset \mathbb{S}^2$ around $\mathcal{S}_0'(v)$ that contains the vertex and stubs of all incident edges. Because each incident edge begins as an equiangular polyline segment, the local geometry at v consists of a rigid star whose inter-stub angles are precisely those recorded in the angle system A_0. Since $A_0 = A_1$, there exists a corresponding disk $D_v^{(1)}$ around $\mathcal{S}_1'(v)$ whose embedded star has the same combinatorial and angular structure, so $D_v^{(0)}$ and $D_v^{(1)}$ are congruent. We now treat each disk as a rigid body.

Let $F_v: [0,1] \times D_v^{(0)} \to \mathbb{S}^2$ be the prescribed rigid motion transporting $D_v^{(0)}$ to $D_v^{(1)}$ by following $\zeta_v(t)$ and applying a rotation $\rho_v(t)$ within the tangent frame to align the stars at $t = 0$ and $t = 1$. Let

$$D^{(0)} = \bigcup_{v \in \mathcal{G}} D_v^{(0)}, \qquad D^{(1)} = \bigcup_{v \in \mathcal{G}} D_v^{(1)}$$

be the union of all vertex disks. Because the vertex trajectories $\tau_v(t)$ are disjoint for all t, as they arise from the ambient isotopy $\widetilde{H}$, we may choose the radius of all disks sufficiently small so that the combined map

$$F: [0,1] \times D^{(0)} \to \mathbb{S}^2 \text{ s.t. } \ F(t,x) = \Big\{F_v(t,x), x \in D_v^{(0)}, v \in \mathcal{G}\Big\}$$

is an isotopy of the submanifold D. Since D is a compact 2-manifold with boundary, the Isotopy Extension Theorem provides an ambient isotopy

$$\tilde{F}: [0,1] \times \mathbb{S}^2 \to \mathbb{S}^2 \quad \text{ s.t. } \ \tilde{F}(t,x) = F(t,x), x \in D^{(0)}, t \in [0,1]$$

This isotopy rigidly transports each vertex disk to its corresponding target disk while preserving all inter-stub angles, i.e., $\tilde{F}(1, D^0) = D^{(1)}$. Define a new graph embedding

$$\mathcal{S}_F' = \tilde{F}(1,\cdot) \circ \mathcal{S}_0'$$

where $\mathcal{S}_F'$ and $\mathcal{S}_1'$ coincide within the disk union $D^{(1)}$, i.e. $\mathcal{S}_F'(\mathcal{G}) \cap D^{(1)} = \mathcal{S}_1'(\mathcal{G}) \cap D^{(1)}$, and they induce the same rotation system. Let

$$\Sigma \coloneqq \mathbb{S}^2 \backslash \mathrm{int}\left(D^{(1)}\right)$$

be a "punctured" sphere, which is a compact surface with boundary $\partial\Sigma$. Define

$$\mathcal{A}_F \coloneqq \mathcal{S}_F'(\mathcal{G}) \cap \Sigma, \qquad \mathcal{A}_1 \coloneqq \mathcal{S}_1'(\mathcal{G}) \cap \Sigma$$

as two finite collections of pairwise disjoint properly embedded arcs in Σ. We then construct refined graph embedding $\mathcal{S}_F''(\mathcal{G})$ and $\mathcal{S}_1''(\mathcal{G})$ on Σ by treating each intersection point with $\partial D^{(1)}$ as a vertex, each boundary segment between consecutive intersection points as an edge, and each arc in $\mathcal{A}_F$ or $\mathcal{A}_1$ as an edge. Since $\mathcal{S}_F'$ and $\mathcal{S}_1'$ agree on the vertex disks and share the same angle system, the induced rotation systems of $\mathcal{S}_F''$ and $\mathcal{S}_1''$ are identical. Applying Heffter-Edmonds-Ringel Rotation Principle again yields a boundary-fixing homeomorphism

$$\psi: \Sigma \to \Sigma, \qquad \mathcal{S}_1'' = \psi \circ \mathcal{S}_F'', \qquad \psi|_{\partial\Sigma} = \mathrm{id}|_{\partial\Sigma}$$

Because both refined embeddings are cellular on Σ, i.e., $\Sigma\backslash\mathcal{S}_F''(\mathcal{G})$ and $\Sigma\backslash\mathcal{S}_1''(\mathcal{G})$ are disjoint unions of disk faces, Σ deformation-retracts onto each refined graph embedding *(80)*. Since the two refined graph embeddings represent the same abstract graph, they are homotopy equivalent to one-dimensional CW complexes. Because Σ deformation-retracts onto each refined graph embedding, this equivalence implies that the homeomorphism ψ is homotopic to the identity relative to $\partial\Sigma$. By the Baer-Epstein theorem *(81)*, ψ is therefore isotopic to the identity relative to the boundary, yielding an ambient isotopy

$$\tilde{J}: [0,1] \times \Sigma \to \Sigma, \qquad \tilde{J}(1,\cdot) = \psi, \qquad \tilde{J}(t, \partial\Sigma) = \partial\Sigma \text{ for all } t$$

This isotopy can be extended to an ambient isotopy of the entire sphere $\mathbb{S}^2$ by defining

$$\widetilde{K}: [0,1] \times \mathbb{S}^2 \to \mathbb{S}^2, \qquad \widetilde{K}(t,x) = x \text{ for all } x \in D^{(1)}, \qquad \widetilde{K}(t,x) = \tilde{J}(t,x) \text{ for all } x \in \Sigma$$

which fixes the disk union $D^{(1)}$ pointwise while acting as $\tilde{J}$ on Σ. By construction, $\widetilde{K}$ preserves all vertex disks and carries the graph embedding $\mathcal{S}_F'$ to $\mathcal{S}_1'$. Hence, concatenating the isotopies $\tilde{F}$ and $\widetilde{K}$ yields an ambient isotopy

$$\tilde{I}: [0,1] \times \mathbb{S}^2 \to \mathbb{S}^2 \text{ s.t. } \tilde{I}(t,\cdot) = \begin{cases} \tilde{F}(2t,\cdot) & , t \in [0,0.5] \\ \widetilde{K}(2t-1,\cdot), & t \in [0.5,1] \end{cases}$$

that $\tilde{I}$ preserves angle system and transforms the embedding $\mathcal{S}_0'$ into $\mathcal{S}_1'$. Restricting $\tilde{I}$ to the embedded graph yields an isotopy of graph embeddings

$$I: [0,1] \times \mathcal{S}_0'(\mathcal{G}) \to \mathbb{S}^2, \qquad I \circ \mathcal{S}_0' = \mathcal{S}_1'$$

Since the outer-face boundary permutations of the two graph embeddings coincide, i.e., $\pi_\infty^{(0)} = \pi_\infty^{(1)}$, the one-point compactification identifies the point at infinity ∞ with corresponding faces of the two spherical embeddings. Consequently, ∞ may be chosen to remain disjoint from the graph

throughout the isotopy I. Removing this point restricts the isotopy of graph embeddings on $\mathbb{S}^2$ to an isotopy of planar embeddings in $\mathbb{R}^2$.

We have thus shown that an angle-system–preserving isotopy between the shapes, i.e., the graph embeddings, of two configurations exists if and only if they share the same angle system and the same outer-face boundary permutation, namely $A_0 = A_1$ and $\pi_\infty^{(0)} = \pi_\infty^{(1)}$. This result justifies our choice of including both the angle system and the outer face boundary permutation in the configuration representation: together, they encode the essential topological and geometric information of a configuration and provide a rigorous and effective criterion for determining kinematic equivalence between configurations. Moreover, this framework applies more broadly to the kinematic analysis of all planar modular mechanisms with rigid connections.

Remark. *It is well known that planar graph embeddings can be represented combinatorially by their rotation system together with the outer face boundary permutation (82). The result proved above may be viewed as a geometric extension of this classical representation: by incorporating the angle system, we augment the purely combinatorial description with local geometric information and relate directly to continuous deformations arising in motion planning.*

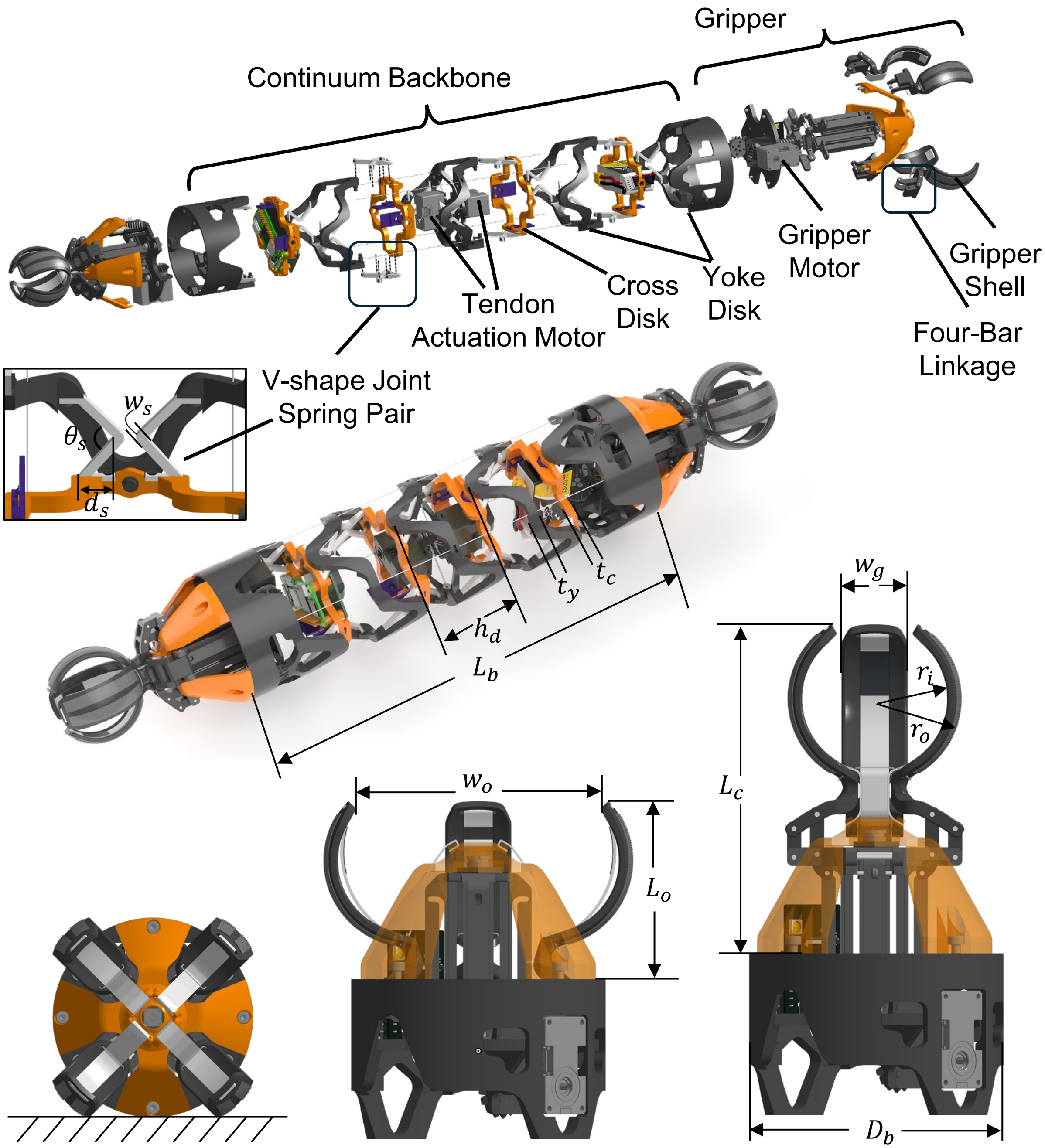

Fig. S1. Design Specifications of a single module.

The bottom left figure shows that when the grippers fully open, their outer edges align with the module's outer circumference and become tangent to the ground plane, providing a stable resting surface.

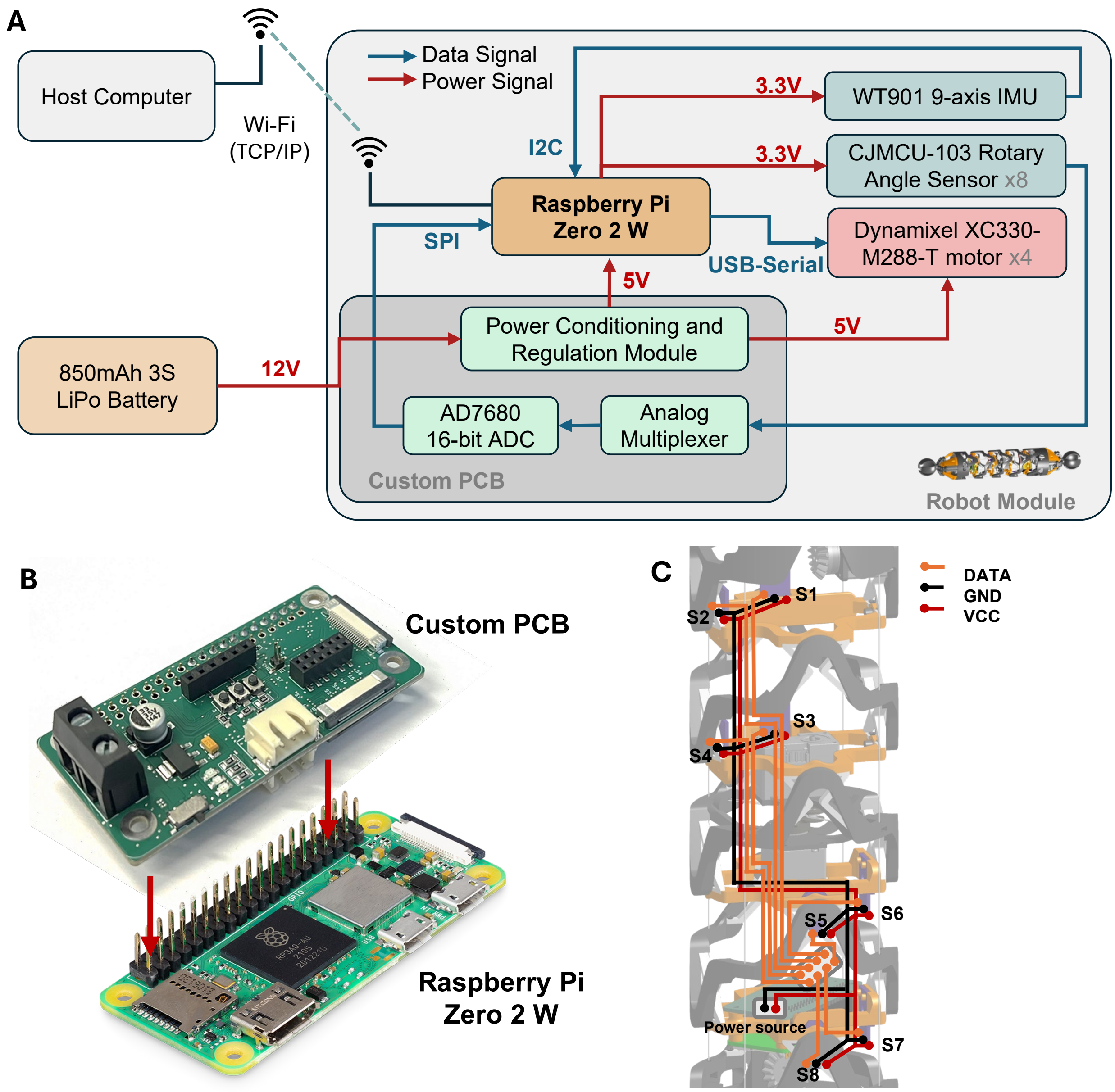

Fig. S2. Electronic system.

(**A**) Electronics diagram of the MSRCR system, including power electronics, central controller, motors and sensors. (**B**) Custom PCB as an extension board for Raspberry Pi Zero 2 W. (**C**) Signal and power chain diagram of eight angle sensors.

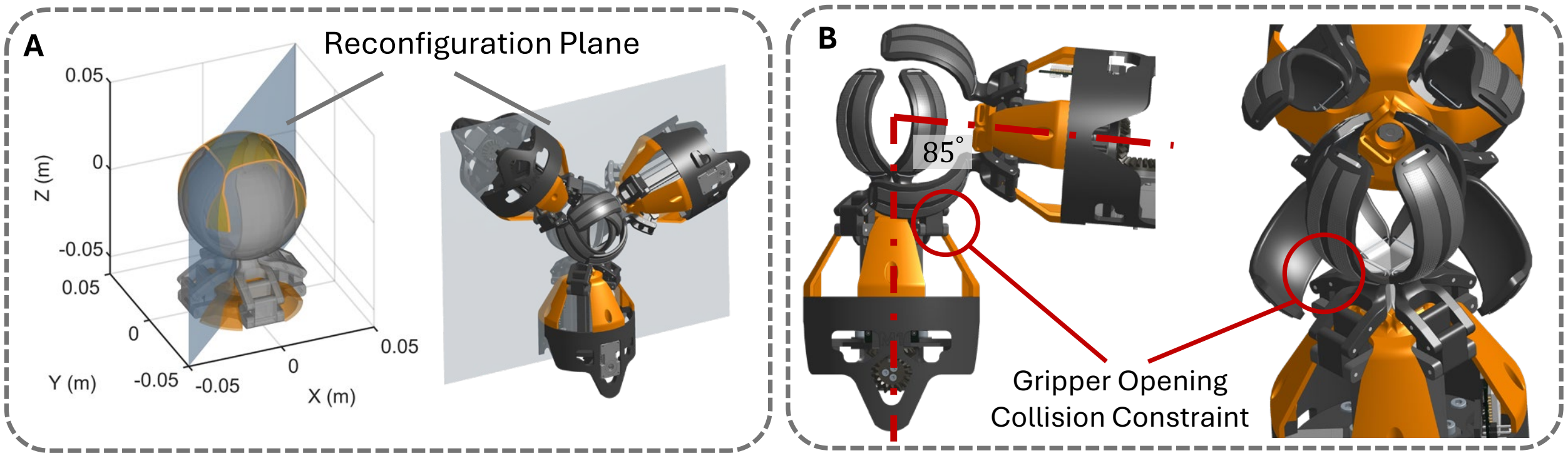

Fig. S3. Planar reconfiguration plane and in-plane docking constraint.

(**A**) Illustration of the planar reconfiguration setting used in the planning problem. During reconfiguration, each module rests on the support surface, and the relevant docking interaction is evaluated in the reconfiguration plane passing through the gripper shell gaps. (**B**) In-plane docking feasibility is determined by the collision-free closure geometry of the grippers. For the current gripper design, adjacent docking directions must be separated by more than approximately 85° to avoid shell interference during gripper closing.

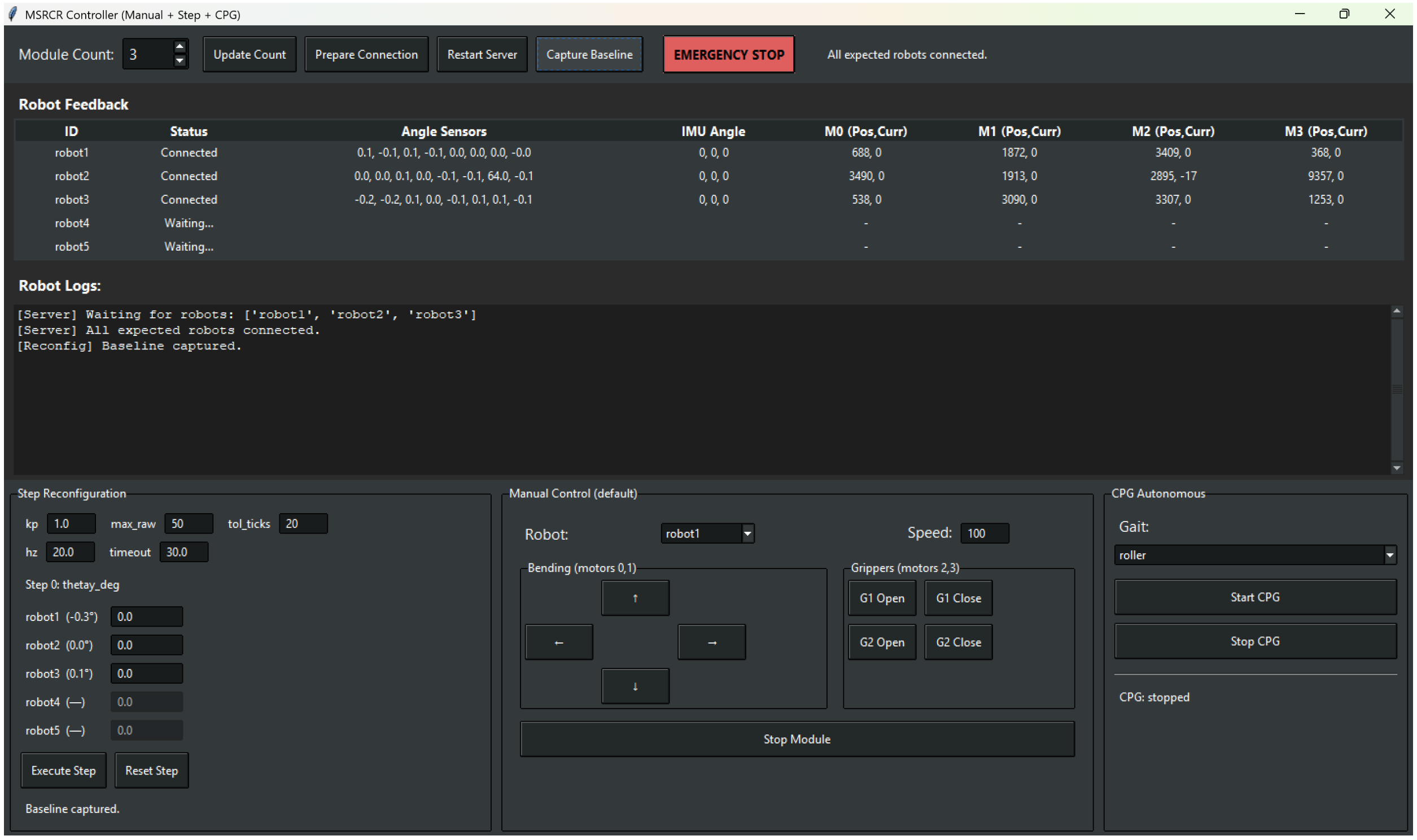

Fig. S4. Graphical user interface for multi-module MSRCR control.

The GUI provides real-time robot feedback, step-wise reconfiguration, manual module control, and autonomous CPG-based locomotion. Connection status, sensor data, and motor states are displayed at the top, with reconfiguration controls on the left, manual actuation in the center, and gait execution on the right.

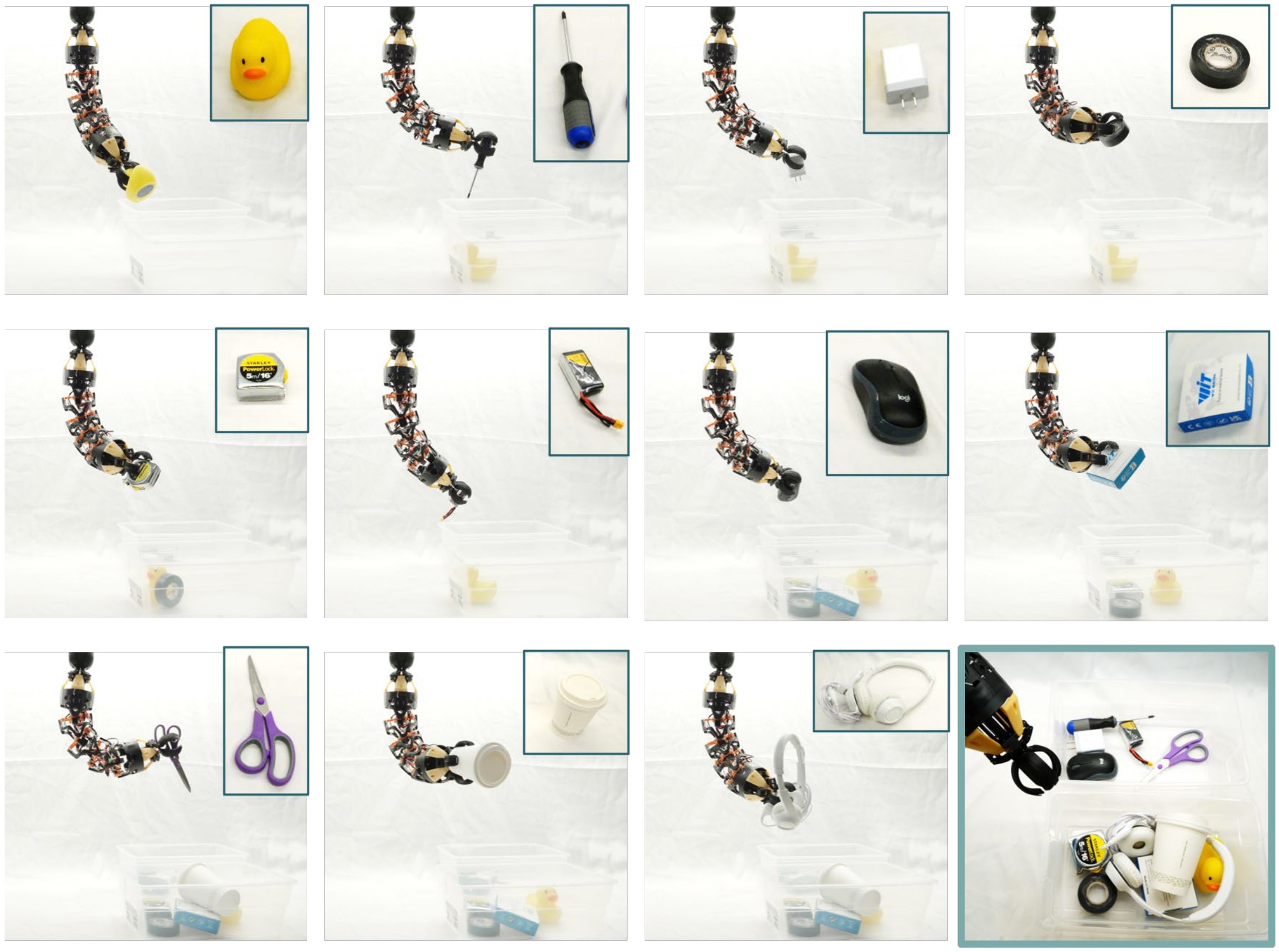

Fig. S5. General object manipulation demonstrations using the docking gripper.

The gripper grasps and lifts a range of everyday objects with varied shapes and materials, including a rubber duck, screwdriver, power adapter, tape roll, tape measure, battery, mouse, box, scissors, cup and headphone set. Each object is grasped and relocated using the same approach–close–lift sequence, illustrating consistent grasp formation across soft, rigid, slender, and irregular geometries.

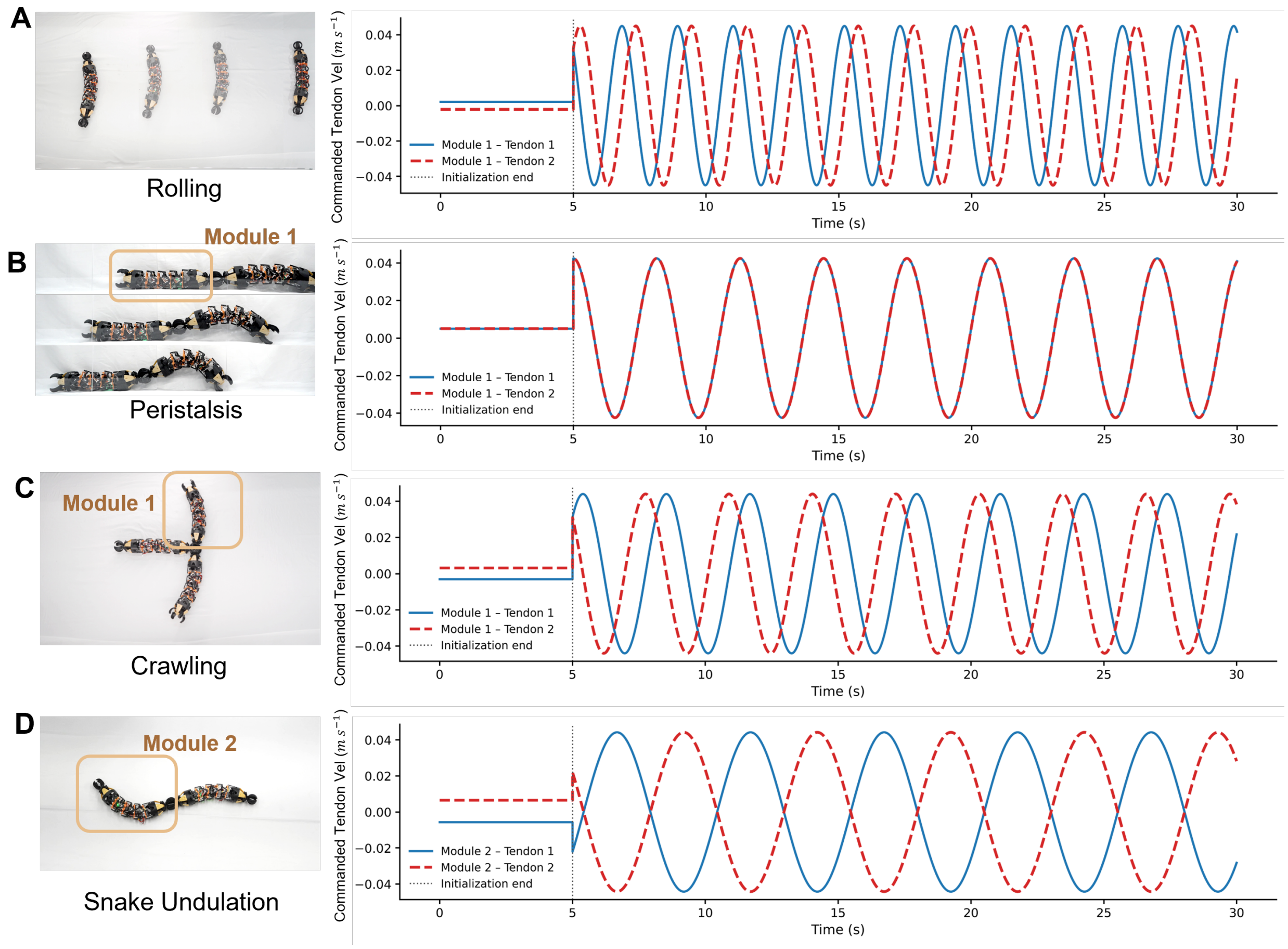

Fig. S6. Representative CPG commanded tendon velocity profiles for distinct locomotion gaits.

Commanded tendon velocity signals for the two antagonistic tendons of the highlighted module in (**A**) rolling gait, (**B**) peristalsis, (**C**) crawling, and (**D**) snake-like undulation. The initialization phase precedes steady-state oscillation. Differences in waveform structure and phase coordination reflect gait-specific CPG coupling patterns under a unified control formulation.

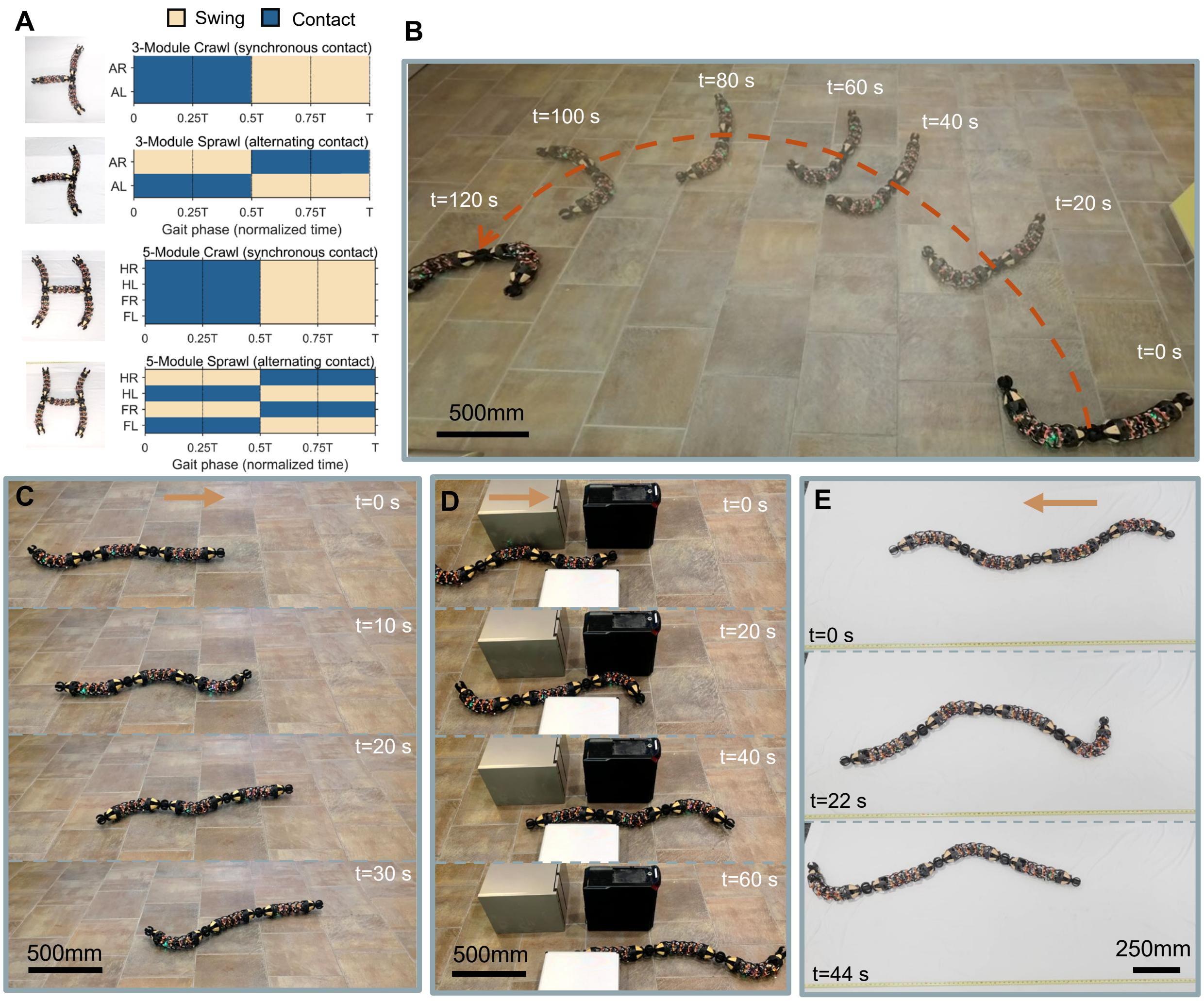

Fig. S7. Extension of the morphology and gait library of the MSRCR.

(**A**) Contact sequence diagrams illustrating crawling and sprawl gaits for three- and five-module configurations. (**B**) Rolling-based arc turning, achieved by imposing different backbone bending angles across two rolling modules, resulting in a biased rolling trajectory. (**C–D**) Snake locomotion with passive wheels, demonstrating forward traversal and obstacle negotiation. (**E**) Four-module snake gait without passive wheels, where coordinated lateral undulation combined with upper bending, similar to the two- and three-module cases, enables effective propulsion through distributed body–ground contact.

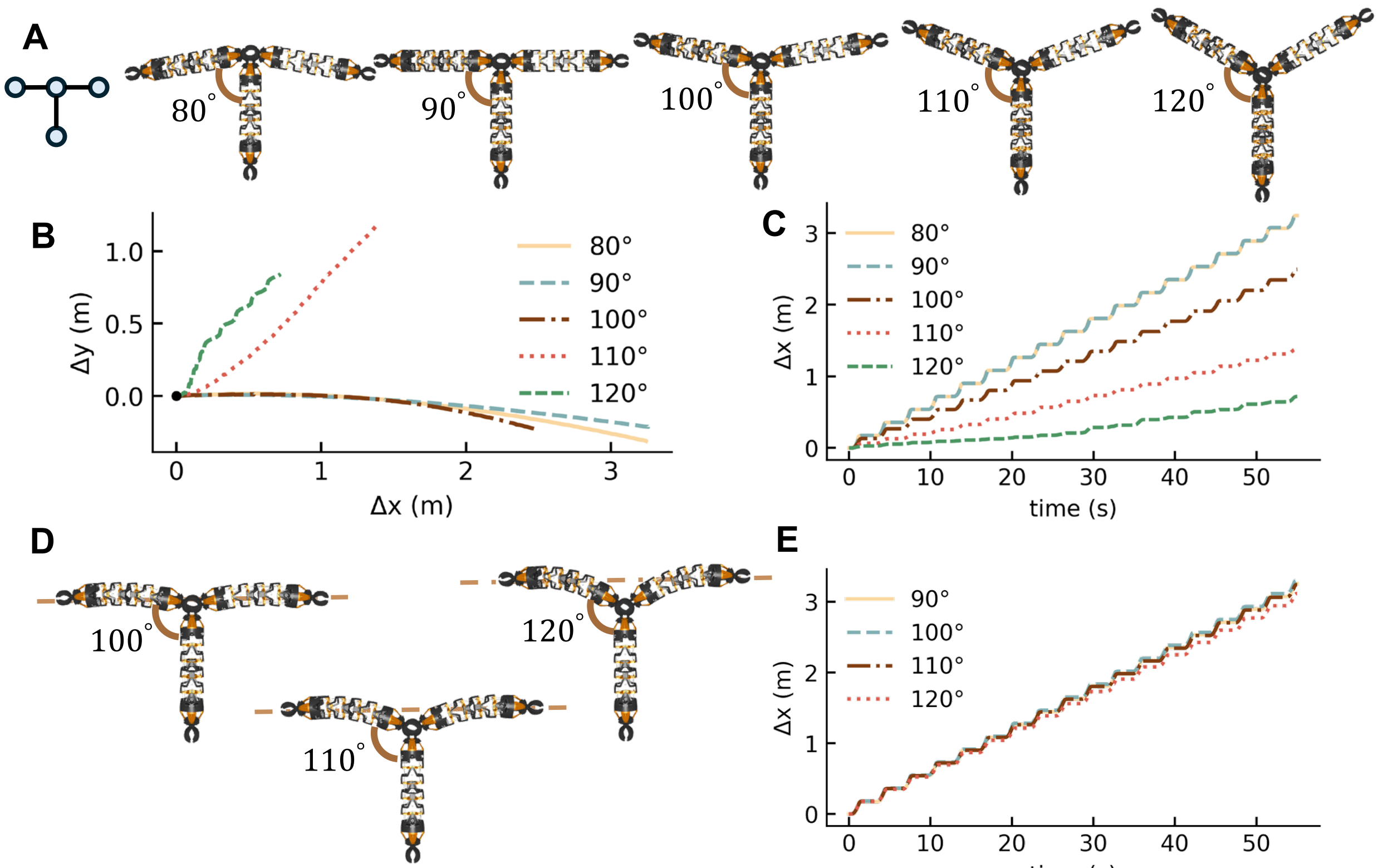

Fig. S8. Morphology-dependent locomotion under a shared CPG controller.

(**A**) A three-module crawler with the same topology but varying inter-module connection angles exhibits markedly different locomotion despite identical CPG parameters. (**B, C**) Trajectories and forward displacement show that the 80° and 90° morphologies achieve similar performance, while the forward motion decreases a lot for 100°, 110°, and 120°, respectively, relative to 90°. (**D, E**) Changing the initial bias of the CPG to shift the neutral bending configuration eliminate the difference.

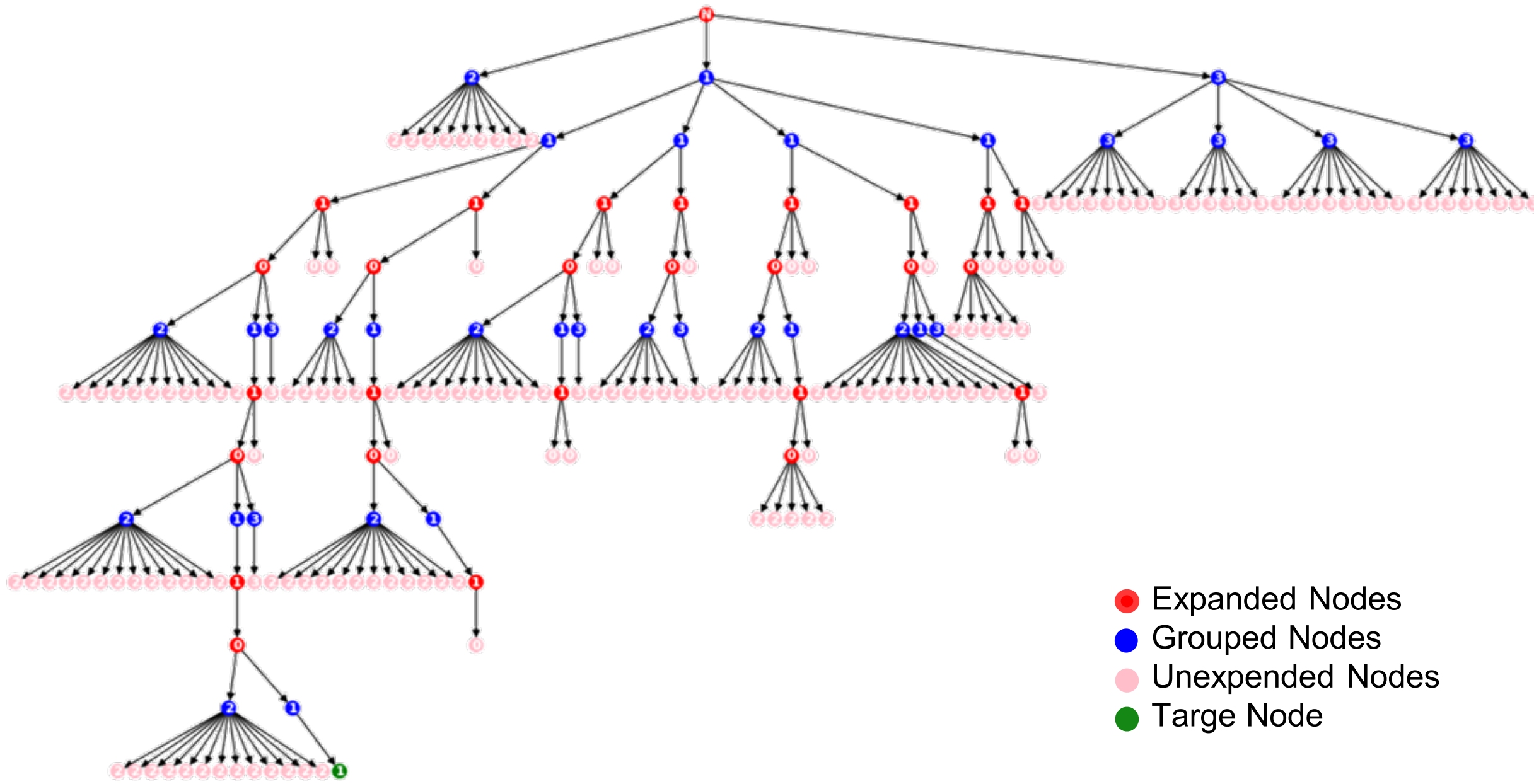

Fig. S9. Search Tree Structure of HEART-MCTS for MSRCR task planning.

The search tree is structured by action type, with nodes corresponding to constructive (1 and 3), auxiliary (2), and releasing (0) actions. Under the expansion-as-simulation strategy, a node is evaluated at the time of expansion; thus, all evaluated nodes correspond exactly to expanded nodes.

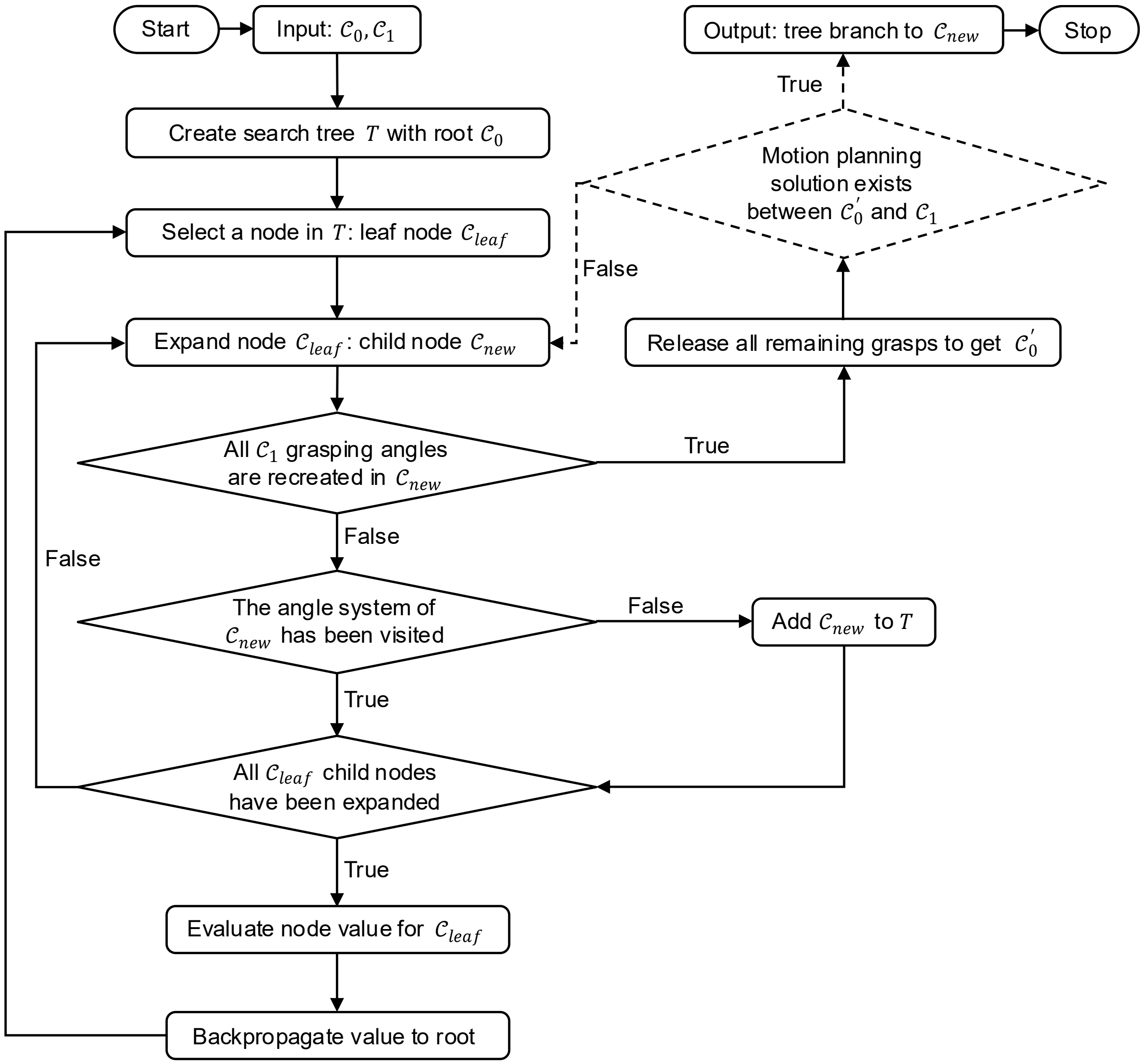

Fig. S10. Flow chart of the MSRCR self-reconfiguration planning algorithm.

Given an initial configuration $\mathcal{C}_0$ and a target configuration $\mathcal{C}_1$, the planner incrementally expands a search tree rooted at $\mathcal{C}_0$ and reconstructs all grasping angles of $\mathcal{C}_1$ through constructive grasping actions. The algorithm follows the standard MCTS cycle of selection, expansion, evaluation (realized via expansion-as-simulation), and backpropagation, with transposition handling implemented by comparing configuration angle systems. The search terminates upon reaching a node kinematically equivalent to $\mathcal{C}_1$. Although reconstructing all target grasping angles does not, in principle, guarantee kinematic equivalence, we empirically observe that this condition always holds in practice; accordingly, the explicit motion-planning verification is omitted during task planning, as indicated by dashed lines.

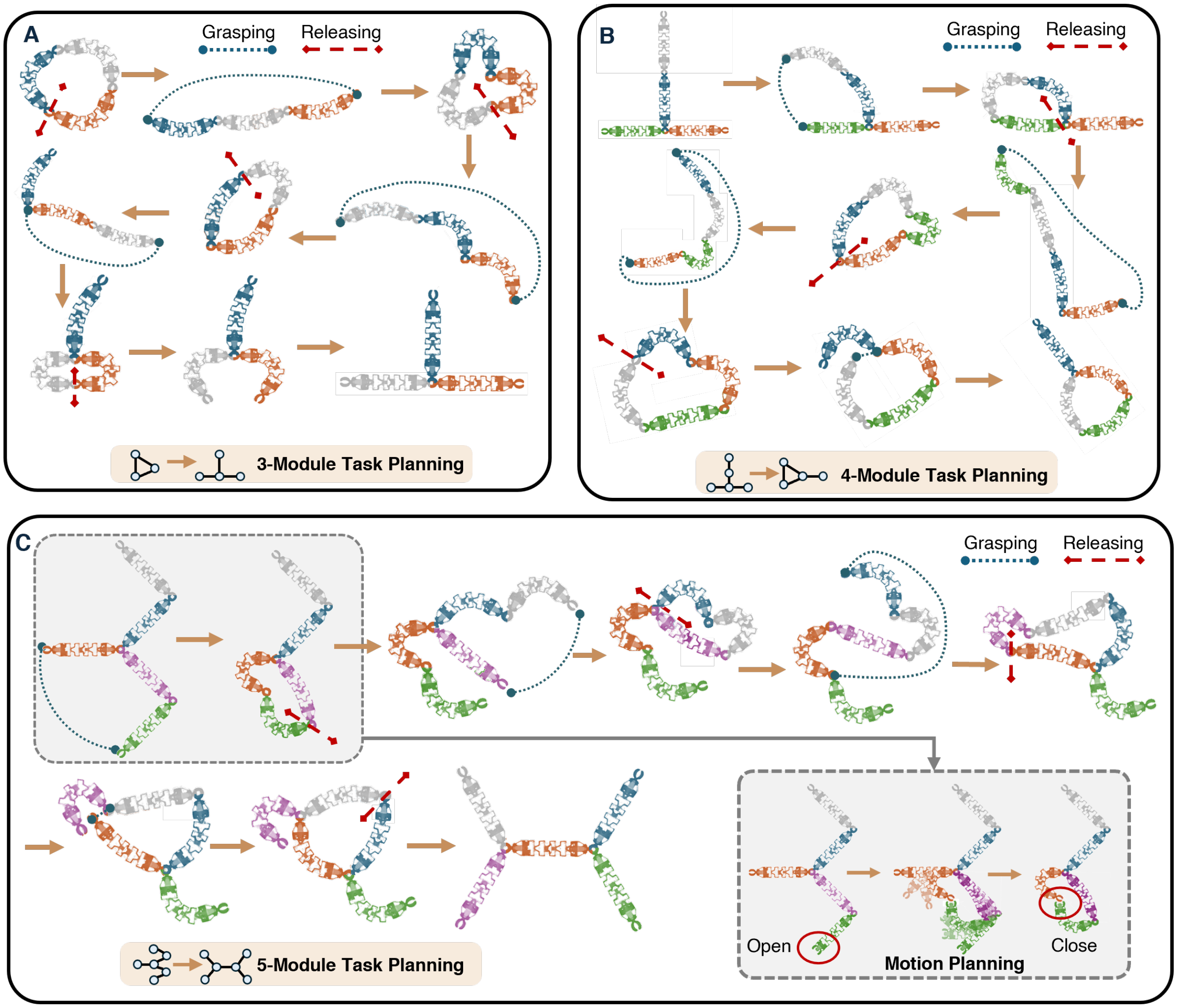

Fig. S11. Example task and motion planning for self-reconfiguration between configurations outside the gait library.

(A–C) Representative planning results for 3-module (A), 4-module (B), and 5-module (C) systems.

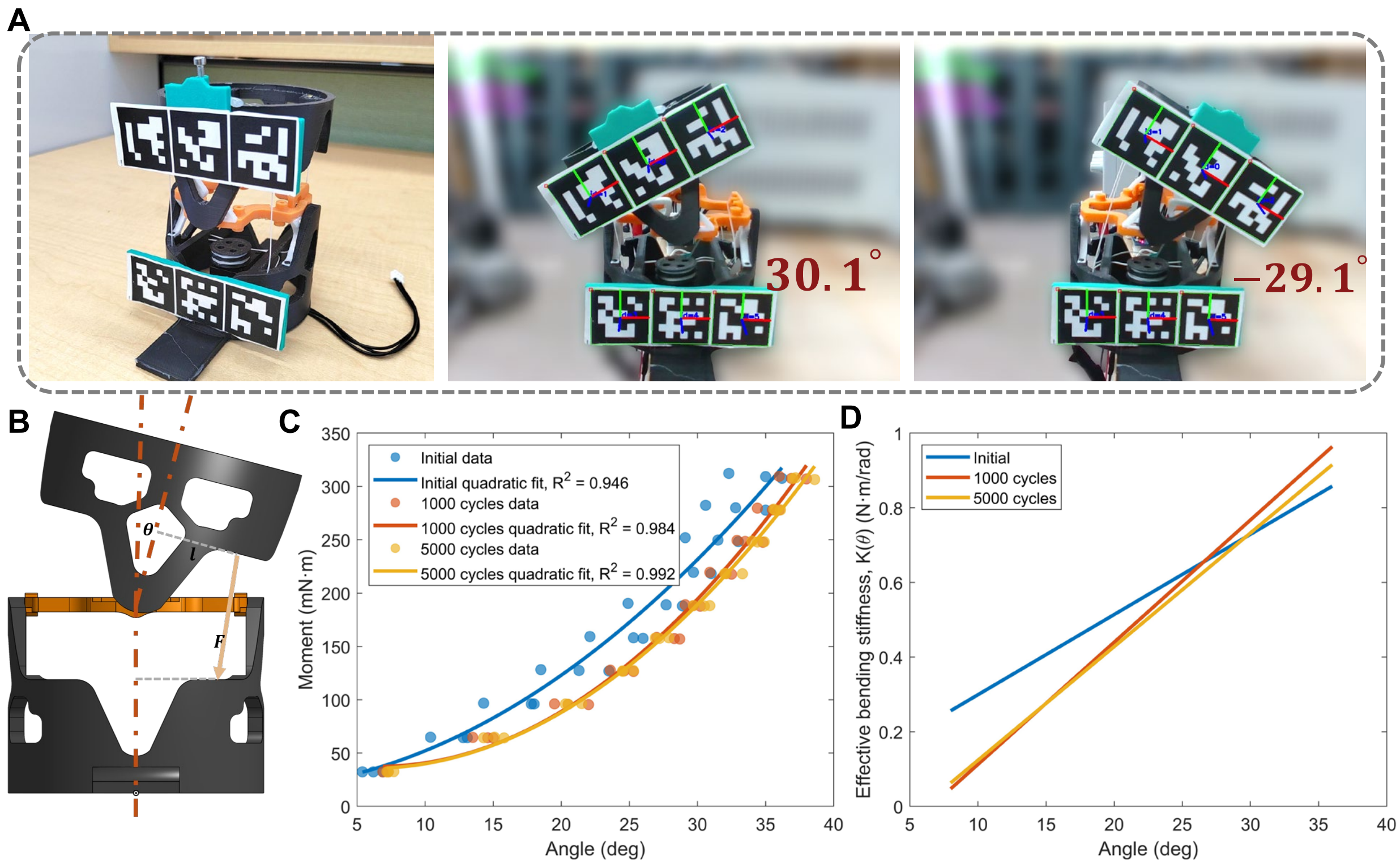

Fig. S12. Durability test of the TPU joint spring pair.

(**A**) Experimental setup for cyclic bending of the joint spring pair using visual marker tracking. Representative bending states show positive and negative bending angles of approximately 30°. (**B**) Schematic of the joint spring pair force loading condition. (**C**) Moment-angle responses before cycling, after 1000 cycles, and after 5000 cycles. Scatter points show measured data, and solid lines show quadratic fits. (**D**) Effective bending stiffness $K(\theta)$ derived from the fitted moment-angle curves. The spring pair shows an initial settling response after cycling, especially at small bending angles, while maintaining comparable stiffness at larger locomotion-relevant bending angles after 5000 cycles.

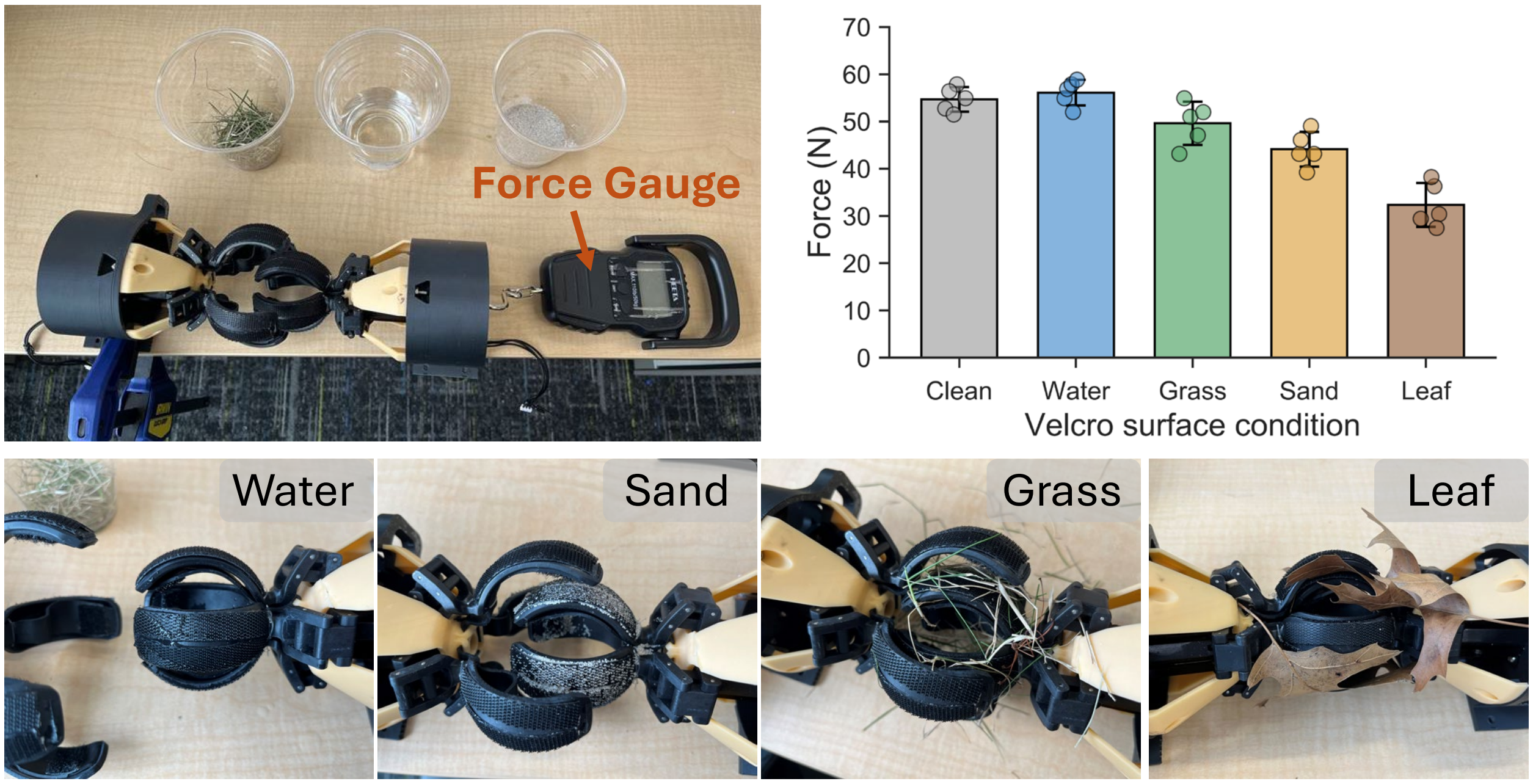

Fig. S13. Velcro-assisted docking performance under surface contamination.

Water, grass, and sand were applied to the gripper/Velcro contact surface, while leaf debris was placed between the two gripper shells to partially block shell-shell contact. Bars show the mean peak pulling force over five trials, error bars indicate standard deviation.

Table S1. Design specifications of each module.

Symbol	Description	Unit	Value
L_b	Length of the continuum backbone	mm	304
h_b	Height of a single segment	mm	58
t_y	Thickness of a yoke disk	mm	6
t_c	Thickness of a cross disk	mm	5
w_g	Width of a gripper shell	mm	23
r_i	Inner radius of the spherical gripper	mm	27.25
r_o	Outer radius of the spherical gripper	mm	30
D_b	Diameter of the continuum backbone	mm	88.4
L_c	Length of a fully closed docking gripper	mm	114.6
L_o	Length of a fully opened docking gripper	mm	62.1
w_o	Opening width of the docking gripper	mm	86.5
w_s	Width of the V-shape spring pair	mm	2.5
d_s	Depth of the V-shape spring pair	mm	10.3
θ_s	Angle of the V-shape spring pair	°	92.6

Table S2. Detailed CPG parameters

For planar gaits (snake undulation, Peristalsis), only the y axis produces bending ($A_x = 0$). The x oscillators remain in the network but contribute no motion, so only the between-module phase offsets on the y axis $\left(\phi_y^{m \to n}\right)$are physically relevant.

Module Number	Morphology Type	Gait Type	Env	ω	Init Bias	Amplitude	Within Module Phase Coupling	Between Module Phase Coupling
1	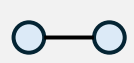	Roll	Real	3.0	/	$A_1 = 0.015$	$\phi_{x\to y}^1 = 0.25, \phi_{y\to x}^1 = -0.25$	/
	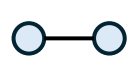	Brachiation	Real		/	/	/	/
2	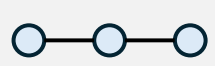	Roll	Real	3.0	/	$A_1 = 0.015$<br>$A_2 = 0.015$	$\phi_{x\to y}^1 = 0.25, \phi_{y\to x}^1 = -0.25$<br>$\phi_{x\to y}^2 = 0.25, \phi_{y\to x}^2 = -0.25$	$\phi_x^{1\to2} = 0, \phi_x^{2\to1} = 0$<br>$\phi_y^{1\to2} = 0, \phi_y^{2\to1} = 0$
	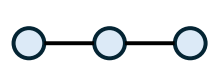	Roll - turn	Real	1.1	/	$A_1 = -0.04$<br>$A_2 = -0.02$	$\phi_{x\to y}^1 = 0.25, \phi_{y\to x}^1 = -0.25$<br>$\phi_{x\to y}^2 = 0.25, \phi_{y\to x}^2 = -0.25$	$\phi_x^{1\to2} = 0, \phi_x^{2\to1} = 0$<br>$\phi_y^{1\to2} = 0, \phi_y^{2\to1} = 0$
	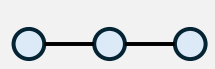	Snake	Real	1.25	$x_{i,y}^0 = 0.005$	$A_{1,x} = A_{2,x} = 0$<br>$A_{1,y} = 0.045$<br>$A_{2,y} = 0.045$	/	$\phi_y^{1\to2} = \frac{2}{3}, \phi_y^{2\to1} = -\frac{2}{3}$
	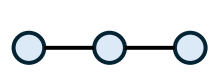	Peristalsis	Real	2	/	$A_{1,x} = 0.03$<br>$A_{2,x} = 0.03$<br>$A_{1,y} = A_{2,y} = 0$	/	$\phi_x^{1\to2} = 0.25, \phi_x^{2\to1} = -0.25$
3	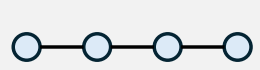	Roll	Real	3.0	/	$A_1 = 0.015$<br>$A_2 = 0.015$<br>$A_3 = 0.015$	$\phi_{x\to y}^1 = 0.25, \phi_{y\to x}^1 = -0.25$<br>$\phi_{x\to y}^2 = 0.25, \phi_{y\to x}^2 = -0.25$<br>$\phi_{x\to y}^3 = 0.25, \phi_{y\to x}^3 = -0.25$	$\phi_x^{1\to2} = 0, \phi_x^{2\to1} = 0$<br>$\phi_x^{2\to3} = 0, \phi_x^{3\to2} = 0$<br>$\phi_x^{3\to1} = 0, \phi_x^{3\to1} = 0$<br>$\phi_y^{1\to2} = 0, \phi_y^{2\to1} = 0$<br>$\phi_y^{2\to3} = 0, \phi_y^{3\to2} = 0$<br>$\phi_y^{3\to1} = 0, \phi_y^{3\to1} = 0$
	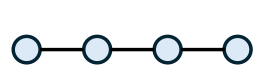	Snake	Real	1.25	$x_{i,y}^0 = 0.005$	$A_{1,x} = A_{2,x} = A_{3,x} = 0$<br>$A_{1,y} = 0.045$<br>$A_{2,y} = 0.045$<br>$A_{3,y} = 0.045$	/	$\phi_y^{1\to2} = -0.83, \phi_y^{2\to1} = 0.83$<br>$\phi_y^{2\to3} = -0.32, \phi_y^{3\to2} = 0.32$<br>$\phi_y^{3\to1} = -0.51, \phi_y^{3\to1} = 0.51$
	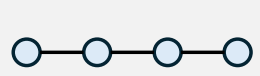	Snake w/ passive wheels	Real	1.5	/	$A_{1,x} = A_{2,x} = A_{3,x} = 0$<br>$A_{1,y} = 0.045$<br>$A_{2,y} = 0.045$<br>$A_{3,y} = 0.045$	/	$\phi_y^{1\to2} = \frac{2}{3}, \phi_y^{2\to1} = -\frac{2}{3}$<br>$\phi_y^{2\to3} = \frac{2}{3}, \phi_y^{3\to2} = -2/3$<br>$\phi_y^{3\to1} = -4/3, \phi_y^{3\to1} = 4/3$
	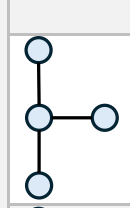	Crawl	Real	2.0	/	$A_1 = 0.02$<br>$A_2 = 0.02$<br>$A_3 = 0$	$\phi_{x\to y}^1 = -0.25, \phi_{y\to x}^1 = 0.25$<br>$\phi_{x\to y}^2 = -0.25, \phi_{y\to x}^2 = 0.25$	$\phi_x^{1\to2} = 0, \phi_x^{2\to1} = 0$<br>$\phi_y^{1\to2} = 0, \phi_y^{2\to1} = 0$
	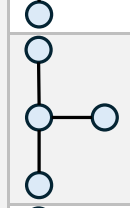	Sprawl	Real	2.0	/	$A_1 = 0.02$<br>$A_2 = -0.02$<br>$A_3 = 0$	$\phi_{x\to y}^1 = -0.25, \phi_{y\to x}^1 = 0.25$<br>$\phi_{x\to y}^2 = -0.25, \phi_{y\to x}^2 = 0.25$	$\phi_x^{1\to2} = 0, \phi_x^{2\to1} = 0$<br>$\phi_y^{1\to2} = 0, \phi_y^{2\to1} = 0$
	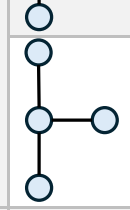	Sprawl - turn	Real	2.0	/	$A_1 = 0.02$<br>$A_2 = -0.01$<br>$A_3 = 0$	$\phi_{x\to y}^1 = -0.25, \phi_{y\to x}^1 = 0.25$<br>$\phi_{x\to y}^2 = -0.25, \phi_{y\to x}^2 = 0.25$	$\phi_x^{1\to2} = 0, \phi_x^{2\to1} = 0$<br>$\phi_y^{1\to2} = 0, \phi_y^{2\to1} = 0$
4	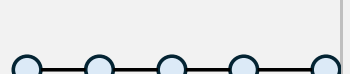	Roll	Real	3.0	/	$A_1 = 0.015$<br>$A_2 = 0.015$<br>$A_3 = 0.015$<br>$A_4 = 0.015$	$\phi_{x\to y}^1 = 0.25, \phi_{y\to x}^1 = -0.25$<br>$\phi_{x\to y}^2 = 0.25, \phi_{y\to x}^2 = -0.25$<br>$\phi_{x\to y}^3 = 0.25, \phi_{y\to x}^3 = -0.25$<br>$\phi_{x\to y}^4 = 0.25, \phi_{y\to x}^4 = -0.25$	$\phi_x^{1\to2} = 0, \phi_x^{2\to1} = 0$<br>$\phi_x^{2\to3} = 0, \phi_x^{3\to2} = 0$<br>$\phi_x^{3\to4} = 0, \phi_x^{4\to3} = 0$<br>$\phi_x^{4\to1} = 0, \phi_x^{1\to4} = 0$<br>$\phi_y^{1\to2} = 0, \phi_y^{2\to1} = 0$<br>$\phi_y^{2\to3} = 0, \phi_y^{3\to2} = 0$<br>$\phi_y^{3\to4} = 0, \phi_y^{4\to3} = 0$<br>$\phi_y^{4\to1} = 0, \phi_y^{1\to4} = 0$

	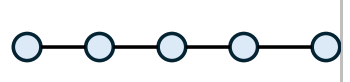	Snake	Real	1.25	$x_{i,y}^{0} = 0.003$	$A_{1,x} = A_{2,x}$<br>$= A_{3,x} = A_{4,x}$<br>$= 0$<br>$A_{1,y} = 0.05$<br>$A_{2,y} = 0.05$<br>$A_{3,y} = 0.05$<br>$A_{4,y} = 0.05$	/	$\phi_y^{1\to 2} = 0.25, \phi_y^{2\to 1} = -0.25$<br>$\phi_y^{2\to 3} = 0.25, \phi_y^{3\to 2} = -0.25$<br>$\phi_y^{3\to 4} = 0.25, \phi_y^{2\to 1} = -0.25$<br>$\phi_y^{4\to 1} = -0.75, \phi_y^{2\to 1} = 0.75$
	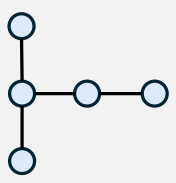	Hybrid<br>(Crawl+Peristalsis)	Real	3.0	/	$A_1 = 0.022$<br>$A_2 = 0.022$<br>$A_3 = 0$<br>$A_{4,x} = 0.02$<br>$A_{4,y} = 0$	$\phi_{x\to y}^{1} = -0.25, \phi_{y\to x}^{1} = 0.25$<br>$\phi_{x\to y}^{2} = -0.25, \phi_{y\to x}^{2} = 0.25$	$\phi_x^{1\to 2} = 0, \phi_x^{2\to 1} = 0$<br>$\phi_x^{2\to 3} = 0.25, \phi_x^{3\to 2} = -0.25$<br>$\phi_x^{3\to 1} = -0.25, \phi_x^{1\to 3} = 0.25$<br>$\phi_y^{1\to 2} = 0, \phi_y^{2\to 1} = 0$
5	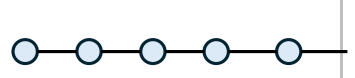	Roll	Sim	3.0	/	$A_1 = 0.015$<br>$A_2 = 0.015$<br>$A_3 = 0.015$<br>$A_4 = 0.015$<br>$A_5 = 0.015$	$\phi_{x\to y}^{1} = 0.25, \phi_{y\to x}^{1} = -0.25$<br>$\phi_{x\to y}^{2} = 0.25, \phi_{y\to x}^{2} = -0.25$<br>$\phi_{x\to y}^{3} = 0.25, \phi_{y\to x}^{3} = -0.25$<br>$\phi_{x\to y}^{4} = 0.25, \phi_{y\to x}^{4} = -0.25$<br>$\phi_{x\to y}^{5} = 0.25, \phi_{y\to x}^{5} = -0.25$	$\phi_x^{1\to 2} = 0, \phi_x^{2\to 1} = 0$<br>$\phi_x^{2\to 3} = 0, \phi_x^{3\to 2} = 0$<br>$\phi_x^{3\to 4} = 0, \phi_x^{4\to 3} = 0$<br>$\phi_x^{4\to 5} = 0, \phi_x^{5\to 4} = 0$<br>$\phi_x^{5\to 1} = 0, \phi_x^{1\to 5} = 0$<br>$\phi_y^{1\to 2} = 0, \phi_y^{2\to 1} = 0$<br>$\phi_y^{2\to 3} = 0, \phi_y^{3\to 2} = 0$<br>$\phi_y^{3\to 4} = 0, \phi_y^{4\to 3} = 0$<br>$\phi_y^{4\to 5} = 0, \phi_y^{5\to 4} = 0$<br>$\phi_y^{5\to 1} = 0, \phi_y^{1\to 5} = 0$
	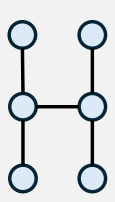	Crawl	Real	2.0	/	$A_1 = 0.022$<br>$A_2 = 0.022$<br>$A_3 = 0.022$<br>$A_4 = 0.022$<br>$A_5 = 0$	$\phi_{x\to y}^{1} = -0.25, \phi_{y\to x}^{1} = 0.25$<br>$\phi_{x\to y}^{2} = -0.25, \phi_{y\to x}^{2} = 0.25$<br>$\phi_{x\to y}^{3} = -0.25, \phi_{y\to x}^{3} = 0.25$<br>$\phi_{x\to y}^{4} = -0.25, \phi_{y\to x}^{4} = 0.25$	$\phi_x^{1\to 2} = 0, \phi_x^{2\to 1} = 0$<br>$\phi_x^{2\to 3} = 0, \phi_x^{3\to 2} = 0$<br>$\phi_x^{3\to 4} = 0, \phi_x^{4\to 3} = 0$<br>$\phi_x^{4\to 1} = 0, \phi_x^{1\to 4} = 0$<br>$\phi_y^{1\to 2} = 0, \phi_y^{2\to 1} = 0$<br>$\phi_y^{2\to 3} = 0, \phi_y^{3\to 2} = 0$<br>$\phi_y^{3\to 4} = 0, \phi_y^{4\to 3} = 0$<br>$\phi_y^{4\to 1} = 0, \phi_y^{1\to 4} = 0$
	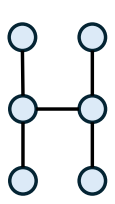	Sprawl	Real	2.0	/	$A_1 = 0.02$<br>$A_2 = 0.02$<br>$A_3 = 0.02$<br>$A_4 = 0.02$<br>$A_5 = 0$	$\phi_{x\to y}^{1} = -0.25, \phi_{y\to x}^{1} = 0.25$<br>$\phi_{x\to y}^{2} = -0.25, \phi_{y\to x}^{2} = 0.25$<br>$\phi_{x\to y}^{3} = -0.25, \phi_{y\to x}^{3} = 0.25$<br>$\phi_{x\to y}^{4} = -0.25, \phi_{y\to x}^{4} = 0.25$	$\phi_x^{1\to 2} = 0.50, \phi_x^{2\to 1} = -0.50$<br>$\phi_x^{2\to 3} = 0.00, \phi_x^{3\to 2} = 0.00$<br>$\phi_x^{3\to 4} = -0.50, \phi_x^{4\to 3} = -0.50$<br>$\phi_x^{4\to 1} = 0.00, \phi_x^{1\to 4} = 0.00$<br>$\phi_y^{1\to 2} = 0.50, \phi_y^{2\to 1} = -0.50$<br>$\phi_y^{2\to 3} = 0.00, \phi_y^{3\to 2} = 0.00$<br>$\phi_y^{3\to 4} = -0.50, \phi_y^{4\to 3} = 0.50$<br>$\phi_y^{4\to 1} = 0.00, \phi_y^{1\to 4} = 0.00$
	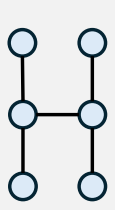	Hybrid<br>(Sprawl + Snake)	Real	1.75	/	$A_1 = 0.02$<br>$A_2 = 0.02$<br>$A_{1,x} = A_{2,x}$<br>$= A_{3,x} = 0$<br>$A_{1,y} = 0.03$<br>$A_{2,y} = 0.03$<br>$A_{3,y} = 0.03$	$\phi_{x\to y}^{1} = -0.25, \phi_{y\to x}^{1} = 0.25$<br>$\phi_{x\to y}^{2} = -0.25, \phi_{y\to x}^{2} = 0.25$	$\phi_x^{1\to 2} = 0.50, \phi_x^{2\to 1} = -0.50$<br>$\phi_y^{1\to 2} = 0.50, \phi_y^{2\to 1} = -0.50$<br>$\phi_y^{2\to 3} = 0.00, \phi_y^{3\to 2} = 0.00$<br>$\phi_y^{3\to 4} = \frac{2}{3}, \phi_y^{4\to 3} = -\frac{2}{3}$<br>$\phi_y^{4\to 5} = \frac{2}{3}, \phi_y^{5\to 4} = -\frac{2}{3}$<br>$\phi_y^{5\to 1} = -\frac{4}{3}, \phi_y^{1\to 5} = \frac{4}{3}$

Table S3. Speed comparison with other soft modular robotic systems.

For chained morphologies, we define the body length as the sum of module lengths along the primary backbone centerline. For branched morphologies, body length is defined as the graph path length between a designated limb pair that dominates support in the executed gait (e.g., left-front to right-front for quadruped-like configurations), yielding a reproducible normalization for speed in body lengths per minute.

Modular Soft Robot	Tethered or Untethered	Reconfigurability	Self-reconfigurability	Manipulation	Module Number	Locomotion Type	Speed (mm/s)	Speed (body length/min)
MSRCR (Ours)	Untethered	Yes	Yes	Yes	1	Rolling	124.80	14.13
					2	Rolling	126.57	7.52
					2	Snake Undulation	6.69	0.40
					2	Peristalsis	17.74	1.05
					3	Rolling	128.23	5.16
					3	Crawling	40.43	2.40
					3	Sprawling	32.24	1.92
					3	Snake	21.26	0.86
					3	Snake w/ wheels	38.49	1.55
					4	Rolling	103.27	3.15
					4	Snake	13.20	0.40
					4	Hybrid	41.22	2.51
					5	Crawling	58.33	3.47
					5	Sprawling	43.37	2.58
					5	Hybrid	37.05	1.49
Tensegrity-blocks (Zhao, et al. 2025) *(35)*	Untethered	Yes	Yes	Yes	1	Walking	3.50	0.40
					2		6.30	0.72
					4		5.70	0.66
StarBlock (Zhao, et al., 2023) *(65)*	Tethered	Yes	Yes	Yes	1	Walking	0.122	0.102
					12	Rolling	1.38	1.15
					19	Quadruped walking	1.16	0.975
Soft Cubes (Vergara et al. 2017) *(34)*	Tethered	Yes	Yes	No	8	Worm-like	1.10	0.375
					8	Cube deformation	0.20	0.3
					8	Two-legged	0.20	0.2
Zou et al. 2018 *(32)*	Tethered	Yes	Only assemble	No	9, 18	Caterpillar-like	5.14	2.0
Zhang et al. 2025 *(63)*	Tethered	Yes	No	No	2	Crawling	2.44	0.80
					2	Wriggling	1.88	0.62
Atia et al. 2022 *(26)*	Tethered	Yes	No	Yes	4	Quadruped walking	2.10	2.14
Meter-scale modular soft robot (Li et al. 2022) *(24)*	Untethered	Yes (with extra connector)	No	Yes	6	Hexapod walking	0.75	0.06
					4	Crawling	3.17	0.12
Limpet II (Sayed et al. 2021) *(64)*	Untethered	Yes	No	No	1,2 or 4	Slip-stick locomotion	35.40	8.50
Arachchige et al. 2021*(29)*	Tethered	Yes (with extra connector)	No	No	3	Rolling	94.13	9.4
					3	Snake undulation	9.23	0.92

Soft robotic blocks (Lee, et al., 2016) *(36)*	Tethered	Yes (with extra connector)	No	No	6	Crawl	1.01	2.3
Kresling origami robot (Wu et al., 2024) *(25)*	Tethered	No	No	No	7	Caterpillar crawling	0.195	0.29
3D Soft Robotic Snake (Wan et al., 2023) *(22)*	Tethered	No	No	No	4	Snake undulation	140.25	0.275

Table S4. Cost of transport comparison across MSRCR gaits and recent soft modular robotic systems.
CoT values are reported for MSRCR and representative soft modular or reconfigurable robotic systems, with emphasis on recent systems from 2023 to 2025.

<table>
<tr><th>Modular Soft Robot</th><th>Tethered or Untethered</th><th>Module Number</th><th>Locomotion Type</th><th>Power / energy source</th><th>CoT Definition</th><th>CoT</th></tr>
<tr><td rowspan="5">MSRCR (Ours)</td><td rowspan="5">Untethered</td><td>1</td><td>Rolling</td><td rowspan="5">Electrical input power</td><td rowspan="5">$P/(mgv)$</td><td>1.20</td></tr>
<tr><td>3</td><td>Rolling</td><td>1.17</td></tr>
<tr><td>3</td><td>Crawling</td><td>3.39</td></tr>
<tr><td>3</td><td>Snake</td><td>9.54</td></tr>
<tr><td>3</td><td>Snake w/ wheels</td><td>5.49</td></tr>
<tr><td rowspan="3">Tensegrity-blocks (Zhao, et al. 2025) *(35)*</td><td rowspan="3">Untethered</td><td>1</td><td rowspan="3">Walking</td><td rowspan="3">Cable actuation power with elastic energy correction</td><td rowspan="3">$\frac{\int_0^{t_f} P(t)dt + U_0 - U_f}{\mu mgd}$</td><td>163</td></tr>
<tr><td>2</td><td>143</td></tr>
<tr><td>4</td><td>178</td></tr>
<tr><td rowspan="3">StarBlock (Zhao, et al., 2023) *(65)*</td><td rowspan="3">Tethered</td><td>1</td><td>Walking</td><td rowspan="3">Not reported</td><td rowspan="3">Not reported</td><td>53200</td></tr>
<tr><td>12</td><td>Rolling</td><td>4782</td></tr>
<tr><td>19</td><td>Quadruped walking</td><td>898</td></tr>
<tr><td>SoftRaft (Zhao, et al., 2026) *(68)*</td><td>Untethered</td><td>1</td><td>Aquatic locomotion</td><td>Electrical input power</td><td>$P/(mgv)$</td><td>6.84</td></tr>
<tr><td rowspan="4">Origami polyhedra-based soft multicellular robot (Hu et al., 2024) *(66)*</td><td rowspan="4">Untethered</td><td rowspan="2">3 cells/ 5 segments</td><td>Peristaltic crawling</td><td rowspan="4">Measured total system energy loss</td><td rowspan="4">$P/v_{measure}$, with P as power-to-weight ratio</td><td>99</td></tr>
<tr><td>Two-anchor crawling</td><td>90</td></tr>
<tr><td rowspan="2">6 cells/ 11 segments</td><td>Peristaltic crawling</td><td>45</td></tr>
<tr><td>Two-anchor crawling</td><td>120</td></tr>
<tr><td>eViper (Cheng, et al., 2023) *(67)*</td><td>Untethered</td><td>2</td><td>Soft undulatory locomotion</td><td>Electrical input power</td><td>$P/(mgv)$</td><td>36.4</td></tr>
</table>

Supplementary Movies

Movie S1. Demonstration of Two- and Three-Gripper Docking and Undocking Processes

Movie S2. Grasping and Manipulation of Diverse Objects Using a Single Module and Docking Gripper

Movie S3. Morphology and Gait Library: 1-Module Rolling and Brachiation

Movie S4. Morphology and Gait Library: 2-Module Rolling, Peristalsis and Snake-like Undulation

Movie S5. Morphology and Gait Library: 3-Module Rolling, Crawling, Sprawling and Snake-like Undulation

Movie S6. Morphology and Gait Library: 4-Module Hybrid Locomotion and Snake-like Undulation

Movie S7. Morphology and Gait Library: 5-Module Sprawling, Crawling and Hybrid Locomotion

Movie S8. Example of Deformation Manifold

Movie S9. Self-reconfiguration Planning: Examples with 3,4,5, and 7 modules

Movie S10. Loco-manipulation and Self-reconfiguration

Movie S11. Demonstration Role of Modular Compliance

Movie S12. Demonstration of GUI Usage

REFERENCES

1. M. Yim, W.-M. Shen, B. Salemi, D. Rus, M. Moll, H. Lipson, K. Eric, G. Chirikjian, Self-reconfigurable robot systems: Challenges and opportunities for the future. *IEEE Robot. Autom. Mag.* **14**, 43–52 (2007).

2. J. Seo, J. Paik, M. Yim, Modular reconfigurable robotics. *Annu. Rev. Control Robot. Auton. Syst.* **2**, 63–88 (2019).

3. J. Daudelin, G. Jing, T. Tosun, M. Yim, H. Kress-Gazit, M. Campbell, An integrated system for perception-driven autonomy with modular robots. *Sci. Robot.* **3**, eaat4983 (2018).

4. D. Zhao, H. Luo, Y. Tu, C. Meng, T. L. Lam, Snail-inspired robotic swarms: A hybrid connector drives collective adaptation in unstructured outdoor environments. *Nat. Commun.* **15**, 3647 (2024).

5. S. Hauser, M. Mutlu, P. A. Léziart, H. Khodr, A. Bernardino, A. J. Ijspeert, Roombots extended: Challenges in the next generation of self-reconfigurable modular robots and their application in adaptive and assistive furniture. *Robot. Auton. Syst.* **127**, 103467 (2020).

6. J. Paulos, N. Eckenstein, T. Tosun, J. Seo, J. Davey, J. Greco, V. Kumar, M. Yim, Automated self-assembly of large maritime structures by a team of robotic boats. *IEEE Trans. Autom. Sci. Eng.* **12**, 958–968 (2015).

7. M. Yim, "Locomotion with unit-modular reconfigurable robot," thesis, Stanford University, Stanford, CA (1995).

8. L. E. Parker, D. Rus, G. S. Sukhatme, "Multiple mobile robot system," in *Springer Handbook of Robotics*, B. Siciliano, O. Khatib, Eds. (Springer International Publishing, 2016), pp. 1335–1384.

9. C. Liu, M. Whitzer, M. Yim, A distributed reconfiguration planning algorithm for modular robots. *IEEE Robot. Autom. Lett.* **4**, 4231–4238 (2019).

10. Z. Yang, S. Zhao, K. Han, J. Qi, N. Zhao, X. Sui, J. Fan, J. Zhao, Y. Zhu, Design of a multi-environmentally adaptable modular self-reconfigurable robot. *IEEE Robot. Autom. Lett.* **9**, 8627–8634 (2024).

11. J. W. Romanishin, K. Gilpin, S. Claici, D. Rus, "3D M-Blocks: Self-reconfiguring robots capable of locomotion via pivoting in three dimensions," in *Proceedings of IEEE International Conference on Robotics and Automation* (*ICRA*) (IEEE, 2015), pp. 1925–1932.

12. Y. Tu, G. Liang, T. L. Lam, "FreeSN: A freeform strut-node structured modular self-reconfigurable robot - Design and implementation," in *Proceedings of IEEE International Conference on Robotics and Automation* (*ICRA*) (IEEE, 2022), pp. 4239–4245.

13. G. Liang, H. Luo, M. Li, H. Qian, T. L. Lam, "FreeBOT: A freeform modular self-reconfigurable robot with arbitrary connection point - Design and implementation," in *Proceedings of IEEE/RSJ International Conference on Intelligent Robots and Systems* (*IROS*) (IEEE, 2020), pp. 6506–6513.

14. P. Swissler, M. Rubenstein, FireAntV3: A modular self-reconfigurable robot toward free-form self-assembly using attach-anywhere continuous docks. *IEEE Robot. Autom. Lett.* **8**, 4911–4918 (2023).

15. G. Liang, D. Wu, Y. Tu, T. L. Lam, Decoding modular reconfigurable robots: A Survey on mechanisms and design. *Int. J. Robot. Res.* **44**, 740–767 (2025).

16. G. P. Wagner, M. Pavlicev, J. M. Cheverud, The road to modularity. *Nat. Rev. Genet.* **8**, 921–931 (2007).

17. P. Dagenais, S. Hensman, V. Haechler, M. C. Milinkovitch, Elephants evolved strategies reducing the biomechanical complexity of their trunk. *Curr. Biol.* **31**, 4727–4737.e4 (2021).

18. Q. Fu, S. W. Gart, T. W. Mitchel, J. S. Kim, G. S. Chirikjian, C. Li, Lateral oscillation and body compliance help snakes and snake robots stably traverse large, smooth obstacles. *Integr. Comp. Biol.* **60**, 171–179 (2020).

19. S. Wu, Q. Ze, J. Dai, N. Udipi, G. H. Paulino, R. Zhao, Stretchable origami robotic arm with omnidirectional bending and twisting. *Proc. Natl. Acad. Sci. U.S.A.* **118**, e2110023118 (2021).

20. S. Kurumaya, B. T. Phillips, K. P. Becker, M. H. Rosen, D. F. Gruber, K. C. Galloway, K. Suzumori, R. J. Wood, A modular soft robotic wrist for underwater manipulation. *Soft Robot.* **5**, 399–409 (2018).

21. S. Abondance, C. B. Teeple, R. J. Wood, A dexterous soft robotic hand for delicate in-hand manipulation. *IEEE Robot. Autom. Lett.* **5**, 5502–5509 (2020).

22. Z. Wan, Y. Sun, Y. Qin, E. H. Skorina, R. Gasoto, M. Luo, J. Fu, C. D. Onal, Design, analysis, and real-time simulation of a 3D soft robotic snake. *Soft Robot.* **10**, 258–268 (2023).

23. Q. Ze, S. Wu, J. Nishikawa, J. Dai, Y. Sun, S. Leanza, C. Zemelka, L. S. Novelino, G. H. Paulino, R. Renee Zhao, Soft robotic origami crawler. *Sci. Adv.* **8**, eabm7834 (2022).

24. S. Li, S. A. Awale, K. E. Bacher, T. J. Buchner, C. Della Santina, R. J. Wood, D. Rus, Scaling up soft robotics: A meter-scale, modular, and reconfigurable soft robotic system. *Soft Robot.* **9**, 324–336 (2022).

25. S. Wu, T. Zhao, Y. Zhu, G. H. Paulino, Modular multi-degree-of-freedom soft origami robots with reprogrammable electrothermal actuation. *Proc. Natl. Acad. Sci. U.S.A.* **121**, e2322625121 (2024).

26. M. G. B. Atia, A. Mohammad, A. Gameros, D. Axinte, I. Wright, Reconfigurable soft robots by building blocks. *Adv. Sci.* **9**, 2203217 (2022).

27. D. D. K. Arachchige, D. M. Perera, S. Mallikarachchi, U. Huzaifa, I. Kanj, I. S. Godage, Soft steps: Exploring quadrupedal locomotion with modular soft robots. *IEEE Access* **11**, 63136–63148 (2023).

28. D. M. Perera, D. D. K. Arachchige, S. Mallikarachchi, T. Ghafoor, I. Kanj, Y. Chen, I. S. Godage, "Teleoperation of soft modular robots: Study on real-time stability and gait control," in *2023 IEEE International Conference on Soft Robotics* (*RoboSoft*) (IEEE, 2023), pp. 1–7.

29. D. D. K. Arachchige, Y. Chen, I. S. Godage, "Soft robotic snake locomotion: Modeling and experimental assessment," in *2021 IEEE 17th International Conference on Automation Science and Engineering* (*CASE*) (IEEE, 2021), pp. 805–810.

30. R. Baines, S. K. Patiballa, J. Booth, L. Ramirez, T. Sipple, A. Garcia, F. Fish, R. Kramer-Bottiglio, Multi-environment robotic transitions through adaptive morphogenesis. *Nature* **610**, 283–289 (2022).

31. D. K. Patel, X. Huang, Y. Luo, M. Mungekar, M. K. Jawed, L. Yao, C. Majidi, Highly dynamic bistable soft actuator for reconfigurable multimodal soft robots. *Adv. Mater. Technol.* **8**, 2201259 (2023).

32. J. Zou, Y. Lin, C. Ji, H. Yang, A reconfigurable omnidirectional soft robot based on caterpillar locomotion. *Soft Robot.* **5**, 164–174 (2018).

33. C. Zhang, P. Zhu, Y. Lin, Z. Jiao, J. Zou, Modular soft robotics: Modular units, connection mechanisms, and applications. *Adv. Intell. Syst.* **2**, 1900166 (2020).

34. A. Vergara, Y. S. Lau, R. F. Mendoza-Garcia, J. C. Zagal, Soft modular robotic cubes: Toward replicating morphogenetic movements of the embryo. *PLOS ONE* **12**, e0169179 (2017).

35. L. Zhao, Y. Jiang, M. Chen, K. Bekris, D. Balkcom, Modular shape-changing tensegrity-blocks enable self-assembling robotic structures. *Nat. Commun.* **16**, 5888 (2025).

36. J. Y. Lee, W. B. Kim, W. Y. Choi, K. J. Cho, Soft robotic blocks: Introducing SoBL, a fast-build modularized design block. *IEEE Robot. Autom. Mag.* **23**, 30–41 (2016).

37. R. M. Grassmann, C. Shentu, T. Hamoda, P. T. Dewi, J. Burgner-Kahrs, Open continuum robotics–one actuation module to create them all. *Front. Robot. AI* **11**, 1272403 (2024).

38. M. Russo, S. M. H. Sadati, X. Dong, A. Mohammad, I. D. Walker, C. Bergeles, K. Xu, D. A. Axinte, Continuum robots: An overview. *Adv. Intell. Syst.* **5**, 2200367 (2023).

39. H. Jin, E. Dong, M. Xu, C. Liu, G. Alici, Y. Jie, Soft and smart modular structures actuated by shape memory alloy (SMA) wires as tentacles of soft robots. *Smart Mater. Struct.* **25**, 085026 (2016).

40. S. Conrad, J. Teichmann, P. Auth, N. Knorr, K. Ulrich, D. Bellin, T. Speck, F. J. Tauber, 3D-printed digital pneumatic logic for the control of soft robotic actuators. *Sci. Robot.* **9**, eadh4060 (2025).

41. A. Western, M. Haghshenas-Jaryani, M. Hassanalian, Golden wheel spider-inspired rolling robots for planetary exploration. *Acta Astronaut.* **204**, 34–48 (2023).

42. T. A. Williams, L. M. Nagy, Developmental modularity and the evolutionary diversification of arthropod limbs. *J. Exp. Zool.* **291**, 241–257 (2001).

43. J. L. Molnar, J. R. Hutchinson, R. Diogo, J. A. Clack, S. E. Pierce, Evolution of forelimb musculoskeletal function across the fish-to-tetrapod transition. *Sci. Adv.* **7**, eabd7457 (2021).

44. J. G. Roscito, K. Sameith, G. Parra, B. E. Langer, A. Petzold, C. Moebius, M. Bickle, M. T. Rodrigues, M. Hiller, Phenotype loss is associated with widespread divergence of the gene regulatory landscape in evolution. *Nat. Commun.* **9**, 4737 (2018).

45. P. J. Bishop, S. E. Pierce, Late acquisition of erect hindlimb posture and function in the forerunners of therian mammals. *Sci. Adv.* **10**, eadr2722 (2024).

46. Y. Cai, H. Xu, Y. Wang, D. Chen, W. Matusik, W. Shou, Y. Chen, Modular self-reconfigurable continuum robot for general purpose loco-manipulation. *IEEE Robot. Autom. Lett.* **10**, 1976–1983 (2025).

47. D. Drotman, S. Jadhav, D. Sharp, C. Chan, M. T. Tolley, Electronics-free pneumatic circuits for controlling soft-legged robots. *Sci. Robot.* **6**, eaay2627 (2021).

48. P. Ramdya, A. J. Ijspeert, The neuromechanics of animal locomotion: From biology to robotics and back. *Sci. Robot.* **8**, eadg0279 (2023).

49. E. Marder, D. Bucher, Central pattern generators and the control of rhythmic movements. *Curr. Biol.* **11**, R986–R996 (2001).

50. A. J. Ijspeert, Amphibious and sprawling locomotion: From biology to robotics and back. *Annu. Rev. Control Robot. Auton. Syst.* **3**, 173–193 (2020).

51. T. Wang, C. Pierce, V. Kojouharov, B. Chong, K. Diaz, H. Lu, D. I. Goldman, Mechanical intelligence simplifies control in terrestrial limbless locomotion. *Sci. Robot.* **8**, eadi2243 (2023).

52. J. Ha, Robotic snake locomotion exploiting body compliance and uniform body tensions. *IEEE Trans. Robot.* **39**, 3875–3887 (2023).

53. J. R. Edmonds Jr., "A combinatorial representation for oriented polyhedral surfaces," thesis, University of Maryland, College Park, MD (1960).

54. F. Hou, W.-M. Shen, "Distributed, dynamic, and autonomous reconfiguration planning for chain-type self-reconfigurable robots," in *Proceedings of IEEE International Conference on Robotics and Automation* (*ICRA*) (IEEE, 2008), pp. 3135–3140.

55. F. Hou, W. M. Shen, Graph-based optimal reconfiguration planning for self-reconfigurable robots. *Robot. Auton. Syst.* **62**, 1047–1059 (2014).

56. H. Luo, S. Member, T. Lun Lam, S. Member, Auto-optimizing connection planning method for chain-type modular self-reconfiguration robots. *IEEE Trans. Robot.* **39**, 1353–1372 (2023).

57. Y. Tu, G. Liang, D. Wu, X. Li, T. L. Lam, Locomotion and self-reconfiguration autonomy for spherical freeform modular robots. *Int. J. Robot. Res.* **45**, 477–500 (2025).

58. J.-T. Saito, M. H. M. Winands, J. W. H. M. Uiterwijk, H. Jaap Van Den Herik, "Grouping nodes for Monte-Carlo tree search," in *Computer Games Workshop* (*CGW*) (Springer, 2007), pp. 276–283.

59. S. Gelly, Y. Wang," Exploration exploitation in Go: UCT for Monte-Carlo Go," *NIPS: Neural Information Processing Systems Conference On-Line Trading of Exploration and Exploitation Workshop*, Canada, 2006.

60. B. Bollobas, A probabilistic proof of an asymptotic formula for the number of labelled regular graphs. *Eur. J. Comb.* **1**, 311–316 (1980).

61. M. H. Dickinson, C. T. Farley, R. J. Full, M. A. R. Koehl, R. Kram, S. Lehman, How animals move: An integrative view. *Science* **288**, 100–106 (2000).

62. D. Bruder, M. A. Graule, C. B. Teeple, R. J. Wood, Increasing the payload capacity of soft robot arms by localized stiffening. *Sci. Robot.* **8**, eadf9001 (2023).

63. Y. Zhang, Y. Li, D. Sui, L. Luan, T. Zheng, Z. Zhang, S. Zhao, F. Zhang, D. Li, Y. Zhu, Design and motion analysis of a soft modular robot for diverse environments. *Soft Robot.* **12**, 662–675 (2025).

64. M. E. Sayed, J. O. Roberts, R. M. McKenzie, S. Aracri, A. Buchoux, A. A. Stokes, Limpet II: A modular, untethered soft robot. *Soft Robot.* **8**, 319–339 (2021).

65. L. Zhao, Y. Wu, W. Yan, W. Zhan, X. Huang, J. Booth, A. Mehta, K. Bekris, R. Kramer-Bottiglio, D. Balkcom, StarBlocks: Soft actuated self-connecting blocks for building deformable lattice structures. *IEEE Robot. Autom. Lett.* **8**, 4521–4528 (2023).

66. F. Hu, C. Zhang, Origami polyhedra-based soft multicellular robots. *Soft Robot.* **11**, 244–259 (2024).

67. H. Cheng, Z. Zheng, P. Kumar, W. Afridi, B. Kim, S. Wagner, N. Verma, J. C. Sturm, M. Chen, "eViper: A scalable platform for untethered modular soft robots," in *Proceedings of IEEE/RSJ International Conference on Intelligent Robots and Systems* (*IROS*) (IEEE, 2023), pp. 604–611.

68. L. Zhao, Y. Jiang, C.-Y. She, A. Q. Li, M. Chen, D. Balkcom, SoftRafts: Floating and adaptive soft modular robots. *npj Robot.* **4**, 8 (2026).

69. E. Todorov, T. Erez, Y. Tassa, "MuJoCo: A physics engine for model-based control," in *Proceedings of IEEE/RSJ International Conference on Intelligent Robots and Systems* (*IROS*) (IEEE, 2012), pp. 5026–5033.

70. D. B. Johnson, Finding all the elementary circuits of a directed graph. *SIAM J. Comput.* **4**, 77–84 (1975).

71. L. Jaillet, J. M. Porta, Path planning under kinematic constraints by rapidly exploring manifolds. *IEEE Trans. Robot.* **29**, 105–117 (2013).

72. L. Jaillet, J. M. Porta, "Asymptotically-optimal path planning on manifolds," in *Robotics: Science and Systems* (Robotics Science and Systems Foundation, 2012), pp. 145–152.

73. Thompsma, Long-toedSalamander.jpg (2005); https://commons.wikimedia.org/wiki/File:Long-toedSalamander.jpg.

74. US National Park Service, Western hognose snake (*Heterodon nasicus*). High resolution image. (614d8699-155d-451f-6761-d3f40052d1ff).JPG (2010); https://commons.wikimedia.org/wiki/File:Western_Hognose_Snake_(Heterodon_nasicus)._High_resolution_image._(614d8699-155d-451f-6761-d3f40052d1ff).JPG.

75. Botswanabugs, *Xanthodes albago* 173076958.jpg (2021); https://commons.wikimedia.org/wiki/File:Xanthodes_albago_173076958.jpg.

76. UdayKiran28, Asiatic-Elephant trunk.jpg (2025); https://commons.wikimedia.org/wiki/File:Asiatic-Elephant_trunk.jpg.

77. L. G. Oversteegen, E. D. Tymchatyn, Extending isotopies of planar continua. *Ann. Math.* **172**, 2105–2133 (2010).

78. B. Mohar, C. Thomassen, *Graphs on Surfaces* (Johns Hopkins Univ. Press, 2001).

79. M. Bhat, N. G. Vlamis, Orientation-preserving homeomorphisms of Euclidean space are commutators. *Proc. Am. Math. Soc. Ser. B* **12**, 38–47 (2025).

80. A. Hatcher, *Algebraic Topology* (Cambridge Univ. Press, 2002).

81. D. B. A. Epstein, Curves on 2-manifolds and isotopies. *Acta Math.* **115**, 83–107 (1966).

82. T. M. Chan, F. Frati, C. Gutwenger, A. Lubiw, P. Mutzel, M. Schaefer, Drawing partially embedded and simultaneously planar graphs. *J. Graph Algorithms Appl.* **19**, 681–706 (2015).
